# Diterpenoid Alkaloids Isolated from *Delphinium brunonianum* and Their Inhibitory Effects on Hepatocytes Lipid Accumulation

**DOI:** 10.3390/molecules27072257

**Published:** 2022-03-30

**Authors:** Huanhuan Ma, Yunxia Ma, Zeren Dawa, Yufeng Yao, Meiqi Wang, Kaihui Zhang, Chenchen Zhu, Fangle Liu, Chaozhan Lin

**Affiliations:** 1 School of Pharmaceutical Sciences, Guangzhou University of Chinese Medicine, Guangzhou 510006, China; 20202110140@stu.gzucm.edu.cn (H.M.); 20191112282@stu.gzucm.edu.cn (Y.M.); 20193112173@stu.gzucm.edu.cn (Y.Y.); wangmeiqi@gzucm.edu.cn (M.W.); 20212110028@stu.gzucm.edu.cn (K.Z.); 2 Institute of Tibetan Medicine, University of Tibetan Medicine, Lasa 850000, China; 3 School of Basic Medical Sciences, Guangzhou University of Chinese Medicine, Guangzhou 510006, China

**Keywords:** *Delphinium brunonianum*, diterpenoid alkaloids, lipid accumulation

## Abstract

This research aimed to excavate compounds with activity reducing hepatocytes lipid accumulation from *Delphinium brunonianum*. Four novel diterpenoid alkaloids, brunodelphinine B–E, were isolated from *D. brunonianum* together with eleven known diterpenoid alkaloids through a phytochemical investigation. Their structures were elucidated by comprehensive spectroscopy methods including HR-ESI-MS, NMR, IR, UV, CD, and single-crystal X-ray diffraction analysis. The inhibitory effects of a total of 15 diterpenoid alkaloids on hepatocytes lipid accumulation were evaluated using 0.5 mM FFA (oleate/palmitate 2:1 ratio) to induce buffalo rat liver (BRL) cells by measuring the levels of triglyceride (TG), total cholesterol (TC), alanine transaminase (ALT), aspartate transaminase (AST), and the staining of oil red O. The results show that five diterpenoid alkaloids—brunodelphinine E (**4**), delbruline (**5**), lycoctonine (**7**), delbrunine (**8**), and sharwuphinine A (**12**)—exhibited significant inhibitory effects on lipid accumulation in a dose-dependent manner and without cytotoxicity. Among them, sharwuphinine A (**12**) displayed the strongest inhibition of hepatocytes lipid accumulation in vitro. Our research increased the understanding on the chemical composition of *D. brunonianum* and provided experimental and theoretical evidence for the active ingredients screened from this herbal medicine in the treatment of the diseases related to lipid accumulation, such as non-alcoholic fatty liver disease and hyperlipidemia.

## 1. Introduction

The metabolic diseases caused by lipid accumulation, such as non-alcoholic fatty liver disease (NAFLD) and obesity, affect an increasing number of people. The global prevalence of NAFLD continues to increase and is now estimated as being up to 25% [1,2]. Despite extensive research on NAFLD, there are still no FDA-approved effective drugs as of now. Although many lipid-lowering drugs (statins), insulin sensitizers (metformin), and antioxidants (vitamin E) have been shown to improve NAFLD, there are still several adverse effects [3]. Therefore, it is of great significance to explore drugs with safety and effective lipid accumulation reducing activity from plants for treatment of the metabolic diseases. 

*Delphinium brunonianum* Royle, belonging to genus *Delphinium* (*Ranunculaceae* family), is a perennial herbaceous plant and mainly distributed at an altitude of 4000–6000 m from Tibet Autonomous Region of China to Nepal and Afghanistan [4]. The dried aerial parts of *D. brunonianum*, as a traditional Chinese aboriginal medicine named “Qiagaobei” in Tibetan, have been widely used for a long time in the treatment of jaundice, influenza, skin itching, and snake bites due to its properties of cooling blood, clearing heat, and detoxification. Traditional Chinese medicines with the above properties are more likely to have anti-inflammatory, antiviral, and antibacterial activity. 

Phytochemical research has presented that alkaloids, flavonoids, and sterols were the predominant composition of *D. brunonianum*. Among them, diterpenoid alkaloids are the characteristic constituents having complex structural features, which mainly include lycoctonine-type C19 and atisine-type C20 diterpenoid alkaloids. The two types of diterpenoid alkaloids are different in terms of skeletal structure. The former, as the main type of diterpenoid alkaloid in *D. brunonianum*, is different from the latter in the number of carbon atoms. Additionally, the latter have an exocyclic double bond on the 16-carbon. Pharmacological studies have proven that diterpenoid alkaloids have many bioactivities with antihypertensive, anti-bacterial, anti-epileptic, diuretic, and anti-inflammatory effects, etc. [5,6,7]. Research showed that several diterpenoid alkaloids may exert anti-inflammatory effects through NF-κB/MAPK and Nrf2/HO-1 [8].

The extract of *D. brunonianum* possesses efficacy in the regulation of metabolic disorders in high fructose-induced rats [9]. Moreover, our previous research exhibited the *D. brunonianum* extract, which is enriched with diterpenoid alkaloids, could alleviate NAFLD by reducing the accumulation of lipids in the liver. So far, fourteen lycoctonine-type diterpenoid alkaloids, three atisine-type diterpenoid, and eleven amide alkaloids have been isolated from *D. brunonianum* [6,10,11,12]. Therefore, information on the chemical composition of diterpenoid alkaloids in *D. brunonianum* is limited. 

**Figure 1:**
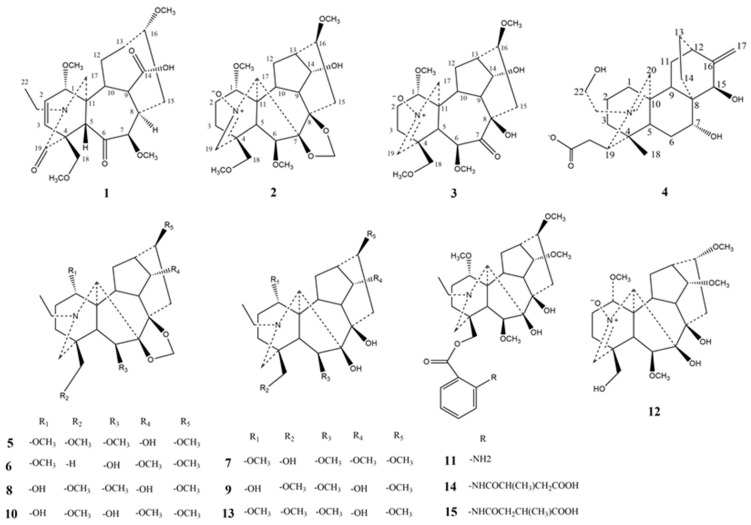
The structures of compounds **1**–**15** isolated from *D. brunonianum*.

To further discover structurally and biologically intriguing active diterpenoid alkaloids in *D. brunonianum*, a phytochemical study of *D. brunonianum* was performed as part of our continuous work, which resulted in the isolation of fifteen diterpenoid alkaloids (as shown in Figure 1), including four novel diterpenoid alkaloids (named brunodelphinine B–E) and eleven known ones. Furthermore, these isolated compounds were evaluated for their inhibitory effects on lipid accumulation in free fatty acid (FFA)-induced BRL cells, aiming to screen a series of entities with potential for development as drugs to treat NAFLD.

## 2. Results and Discussion

### 2.1. Structure Elucidation of Compounds

Compound **1** was obtained as amorphous powder, and it showed a positive reaction tested with Dragendorff’s reagent. Its molecular formula was determined as C_25_H_37_NO_8_ (eight degrees of unsaturation) based on protonated molecular ion at *m*/*z* 480.2592 [M + H]^+^ in the HR-ESI-MS (calculated for C_25_H_38_NO_8_, 480.2597). Its IR spectrum showed absorptions due to the hydroxyl (3435 cm^−1^) and carbonyl (1728, 1671, and 1629 cm^−1^) groups. The ^1^H and ^13^C NMR spectroscopic data (Table 1) of compound **1** exhibited five quaternary carbon signals (*δ*_C_ 40.7, 50.8, 170.2, 174.6, and 202.2), three of which are carbonyl signals (*δ*_C_ 170.2, 174.6, and 202.2), nine methine groups (*δ*_C_ 38.3, 39.4, 45.5, 56.1, 76.88, 77.1, 89.6, 148.8, and 129.6), six methylene groups (*δ*_C_ 21.8, 27.7, 27.9, 42.7, 48.7, and 67.9), four methoxy groups (*δ*_H_ 3.0 (3H, *s*), 3.23 (3H, *s*), 3.61 (3H, *s*), 3.63 (3H, *s*), *δ*_C_ 52.6, 56.0, 59.0, and 51.2), an intracyclic double bond signal (*δ*_H_ 5.98 (1H, *dd*, *J* = 9.7, 1.4 Hz), 7.02 (1H, *tt*, *J* = 19.7, 7.3 Hz), *δ*_C_ 129.6, 148.8), and a N-ethyl signal (*δ*_H_ 1.20 (3H, *t*, *J* = 7.2 Hz), *δ*_C_ 42.7, 11.1). Thus, compound **1** was considered as a lycoctonine-type C19-diterpenoid alkaloid based on the above data.

The HMBC correlations of H-21 (*δ*_H_ 3.67 (1H, *d*, *J* = 3.3 Hz), 3.32 (1H, *d*, *J* = 3.3 Hz)), H-18 (*δ*_H_ 3.67 (1H, *m*)), H-17 (*δ*_H_ 3.28 (1H, *m*)) with C-19 (*δ*_C_ 170.2) and H-3 (*δ*_H_ 7.02 (1H, *tt*, *J* = 19.7, 7.3 Hz)), H-1 (*δ*_H_ 3.82 (1H, *m*)), H-10 (2.87 (1H, *dd*, *J* = 9.7, 5.0 Hz)) with C-6 (*δ*_C_ 202.2) suggested that two carbonyl groups might positioned at C-19 and C-6, respectively. The location of double bond group was attached to C-2 and C-3 positions on the basis of HMBC correlations of H-1 (*δ*_H_ 3.82 (1H, *m*)) with C- 3 (*δ*_C_ 56.0), H-2 (*δ*_H_ 5.98 (1H, *dd*, *J* = 9.7, 1.4 Hz)) with C-10 (*δ*_C_ 56.1), and H-3 (*δ*_H_ 7.02 (1H, *tt*, *J* = 19.7, 7.3 Hz)) with C-5 (*δ*_C_ 38.3). According to the HMBC correlations of H-8 (*δ*_H_ 2.04 (1H, *s*) with C-14 (*δ*_C_ 174.6), a carboxyl group was considered positioned at C-14. Four methoxy groups was attached to C-1, C-7, C-16, and C-18 position due to the HMBC correlations of 1-OCH_3_ (*δ*_H_ 3.23 (3H, *s*)) with C-1 (*δ*_C_ 89.6), 7-OCH_3_ (*δ*_H_ 3.61 (3H, *s*)) with C-14 (*δ*_C_ 77.1), 16-OCH_3_ (*δ*_H_ 3.31 (3H, *s*)) with C-16 (*δ*_C_ 76.9), and 18-OCH_3_ (*δ*_H_ 3.63 (1H, *s*)) with C-7 (*δ*_C_ 67.9). 

**Figure 2:**
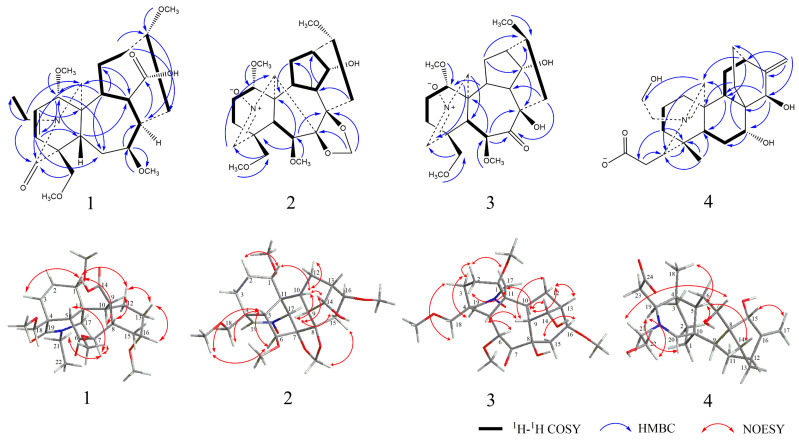
Selected ^1^H–^1^H COSY, HMBC, and NOESY correlations of compounds **1**–**4**.

The relative configuration of compound **1** was deduced by NOESY spectrum. The NOESY correlations of H-1 with H-3/H-8/H-10/H-12, H-8 with H-3/H-9/H-1, H-7 with H-21/H-5/7-OCH_3_, and H-13 with H-12/H-16 indicated *β*-orientation for H-7, H-18, and H-5, and *α*-orientation for H-1, H-14, H-8, and H-16. Finally, compound **1** was determined as 7,17-seco, 6,14-dione, 7*β*,16*α*-methoxy, 18*α*-hydrogen, 19-oxobrowniine (Figure 2), which was named brunodelphinine B.

Compound **2**, a yellow amorphous powder, exhibited positive reaction using Dragendorff’s reagent. Its molecular formula was determined as C_24_H_35_NO_8_ (night degrees of unsaturation) on the basis of the HR-ESI-MS (calculated for C_24_H_36_NO_8_, 466.2441). Its IR spectrum showed absorptions for the hydroxyl (3437 cm^−1^) groups. The ^1^H and ^13^C NMR spectroscopic data (Table 1) of compound **2** displayed the presence of one double bond group (*δ*_H_ 6.78 (1H, *d*, *J* = 1.6 Hz, *δ*_C_ 138.0)), four methoxy groups (*δ*_H_ 3.18 (3H, *s*), 3.33 (3H, *d*, *J* = 2.7 Hz), 3.31 (3H, *d*, *J* = 2.9 Hz), 3.29 (3H, *d*, *J* = 3.2 Hz), *δ*_C_ 56.0, 58.9, 56.7, and 59.3), one methylenedioxy group (*δ*_H_ 5.18 (2H, *d*, *J* = 4.9 Hz), *δ*_C_ 95.0), ten methines (*δ*_C_ 35.6, 41.9, 47.1, 48.4, 73.9, 77.7, 80.2, 81.3, 89.2, and 138.0), five methylene groups (*δ*_C_ 24.4, 28.4, 26.5, 31.4, and 74.3), and four quaternary carbon groups (*δ*_C_ 44.5, 49.5, 80.9, and 90.4). 

HMBC correlations of -O-CH_2_-O- (*δ*_H_ 5.18 (2H, *d*, *J* = 4.9 Hz), *δ*_C_ 95.0) with C-7 (*δ*_C_ 90.4) and C-8 (*δ*_C_ 80.9) indicated a methylenedioxy group locating at the C-7 and C-8 positions. Four methoxy groups were assigned to be positioned at C-1, C-6, C-16, and C-18 due to the HMBC correlations of 1-OCH_3_ (*δ*_H_ 3.18 (3H, *s*)) with C-1 (*δ*_C_ 80.2), 6-OCH_3_ (*δ*_H_ 3.33 (3H, *d*, *J* = 2.7 Hz)) with C-6 (*δ*_C_ 89.2), 16-OCH_3_ (*δ*_H_ 3.31 (3H, *d*, *J* = 2.9 Hz)) with C-16 (*δ*_C_ 81.3), and 18-OCH_3_ (*δ*_H_ 3.29 (3H, *d*, *J* = 3.2 Hz)) with C-18 (*δ*_C_ 74.3). In addition, it was substantiated the position of the hydroxyl group at C-14 by the ^1^H-^1^H COSY correlations of H-14 (*δ*_H_ 3.99 (1H, *d*, *J* = 5.5 Hz)) with H-9 and H-13.

Careful comparison of the ^13^C NMR spectral data with the known compound sharwuphinine A (**12**) revealed that compound **2** might be a deazoethyl compound that contained a nitrone structure and a nitrogen–oxygen double bond with C-19 (*δ*_H_ 6.78 (1H, *d*, *J* = 1.6 Hz), *δ*_C_ 138.0). Our results confirmed that compound **2** is a lycoctonine-type C19-diterpenoid alkaloid. The NOESY correlations of H-5 with H-18 /H-6/18-OCH_3_, H-6 with H-18, H-10 with H-1/H-12/H-9/H-14, and H-15 with H-17, H-9 with H-14/H-15 suggested the *β*-orientation for H-6, H-7, H-8, H-16, and H-18 and the *α*-orientation for H-1 and H-14. Therefore, compound **2** was determined as 14*α*-hydroxy-18*β*-methoxy-Nnitrone (Figure 2), and named as brunodelphinine C.

Compound **3** was isolated as a yellow amorphous powder and showed a positive reaction with Dragendorff’s reagent. Its molecular formula was determined as C_23_H_35_NO_8_ by HR-ESI-MS at *m*/*z* 454.2439 [M + H]^+^ (calculated for C_23_H_36_NO_8_, 454.2446), corresponding to 7 degrees of unsaturation. The IR spectrum showed absorptions for hydroxyl (3447 cm^−1^) groups. The ^1^H and ^13^C NMR spectroscopic data (Table 1) of compound **3** were essentially identical with those of compound **2**, suggesting that **3** also belonged to lycoctonine-type C19-diterpenoid alkaloid. The only difference between **3** and **2** was the presence of a methylenedioxy group (*δ*_H_ 5.18 (2H, *d*, *J* = 4.9 Hz), *δ*_C_ 95.0) in the former, instead of the carbonyl group (*δ*_C_ 209.3) in the latter. This was also supported by 2D-NMR data; the location of one carbonyl group could be assigned at C-7 due to the HMBC correlation of H-6 with C-7 (*δ*_C_ 209.3) and H-15 with C-7 (*δ*_C_ 209.3). By comparison with compound **2**, it was found that compound **3** is also a C19-diterpenoid alkaloids containing deazoethyl group. Finally, **3** was identified as 7,17-seco, 7-keto, 8-hydroxy-brunodelphinine C (Figure 2), named brunodelphinine D. 

Compound **4** was isolated as a white amorphous powder, and it presented a positive reaction under Dragendorff’s reagent. Its molecular formula was ascertained as C_24_H_35_NO_5_ (night degrees of unsaturation), deducing from the quasi-molecular ion peak (M + H)^+^ at *m*/*z* 418.2543 (calculated for C_24_H_36_NO_5_, 418.2586) in HR-ESI-MS. The IR spectrum displayed absorptions bands for hydroxyl (3410 cm^−1^), carboxylate (1594 cm^−1^ and 1384 cm^−1^), and amine salt (2932 cm^−1^ and 2872 cm^−1^) groups. The ^1^H NMR and ^13^C NMR spectroscopic data (Table 1) of compound **4** exhibited one methyl group (*δ*_H_ 0.96 (3H, *s*), *δ*_C_ 25.0) and two methine signals substituted with hydroxyl groups (*δ*_H_ 3.76 (1H, *dd*, *J* = 11.6, 4.1Hz), 4.02 (1H, *s*), *δ*_C_ 67.2, 69.5), and a set of terminal double bond signals (*δ*_H_ 5.00 (1H, s), 4.96 (1H, s), *δ*_C_ 155.8, 109.5), and four quaternary carbon groups (*δ*_C_ 36.3, 42.2, 44.9, and 182.9) that included one carboxyl group (*δ*_C_ 182.9), and of the eleven methylene groups (*δ*_C_ 18.3, 18.9, 24.8, 27.5, 27.8, 34.8, 36.2, 36.3, 58.0, 61.3, and 109.5), one nitrogen-containing substituted methine groups (*δ*_C_ 173.2) and four methine groups (δ_C_ 44.7, 35.5, 39.4, and 66.1). The above results indicate that compound **4** was a C20-diterpenoid alkaloid. 

The four hydroxyl groups were assigned to be positioned at C-19, C-22, C-7, and C-15 due to the HMBC correlations of H-18 (*δ*_H_ 0.96 (3H, *s*)) with C-4 (*δ*_C_ 36.3), C-5 (*δ*_C_ 44.7), C-19 (*δ*_C_ 66.1), H-9 (*δ*_H_ 2.03 (1H, *t*, *J* = 8.6 Hz)) with C-20 (*δ*_C_ 173.2), H-20 (*δ*_H_ 8.60 (1H, *s*)) with C-19 (*δ*_C_ 66.1), C-23 (*δ*_C_ 36.2), H-7 (*δ*_H_ 3.76 (1H, *dd*, *J* = 11.6, 4.1 Hz)) with C-8 (*δ*_C_ 42.2), C-15 (*δ*_C_ 69.5), H-15 (*δ*_H_ 4.02 (1H, *s*)) with C-13 (*δ*_C_ 24.8), C-9 (*δ*_C_ 39.4), and H-17 (*δ*_H_ 5.00 (1H, *s*), 4.96 (1H, *s*)) with C-15 (*δ*_C_ 69.5). 

**Figure 3:**
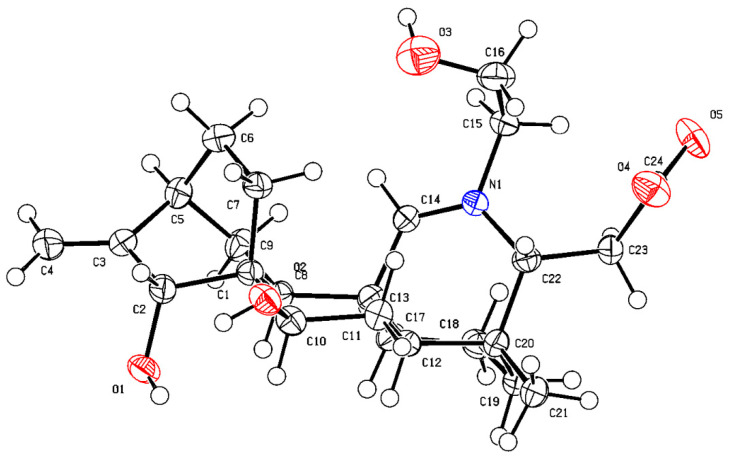
ORTEP drawing of compound **4**.

The NOESY correlations of H-5 with H-2/H-14/H-18, H-7 with H-21, H-15 with H-17, and H-19 with H-20/H-22 indicated the *β*-orientation for C-15, C-18, and the *α*-orientation for C-7. Combined with the single crystal diffraction X-ray experiment, the hydroxyl groups on C-22 at the *β*-position, and the carboxyl group on C-19 was *β*-substituted (Figure 3). Finally, compound **4** was confirmed as 7*α*, 15*β*, 22*β*-hydroxy-19*β*-carboxylatisine (Figure 2), and named brunodelphinine E.

Furthermore, eleven known diterpenoid alkaloids were determined to be delbruline (**5**) [11], delpheline (**6**) [13], lycoctonine (**7**) [11], delbrunine (**8**) [11], delcosine (**9**) [11], uraphine (**10**) [14], anthranoyllycoctonine (**11**) [15], sharwuphinine A (**12**) [16], browniine (**13**) [11], shawurensine (**14**) [17], and delavaine B (**15**) [17] by comparison of their corresponding spectroscopic data with those reported previously in the literature.

### 2.2. Cell Viability of Fifteen Isolated Compound and Positive Drug in BRL Cells

**Figure 4:**
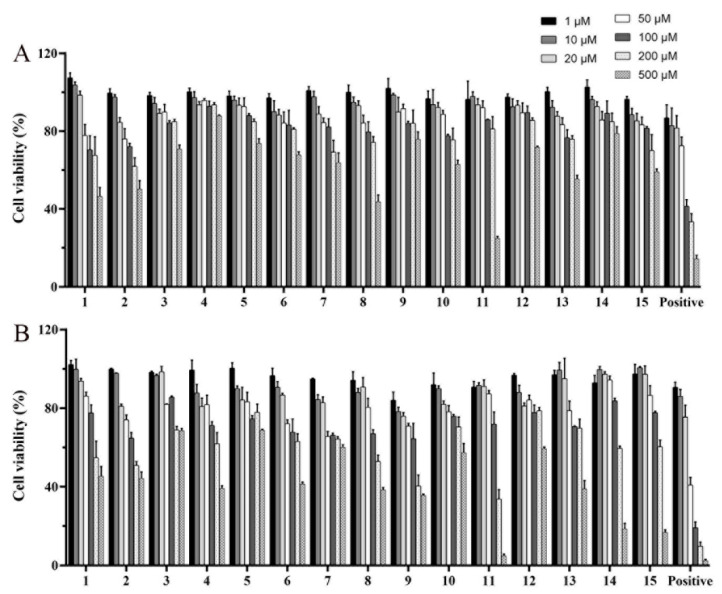
Cell viability of compounds **1**–**15** positive (ATC) with the concentration range of 1–500 μM in BRL cells for 24 h (**A**) and 48 h (**B**).

The cell viability (%) of the isolated diterpenoid alkaloids in the concentration range of 1–500 μM and the time-effectiveness of them for 24 h and 48 h were evaluated using CCK-8 assay (Figure 4). The results show that the cell viability of isolated compounds **1**–**15** and positive (ATC) decreased after 48 h treatment and was generally lower than that of cells treated for 24 h at the same concentration. The IC_50_ values of 15 compounds and positive drug were calculated by SPSS 23.0 software. The results show that the IC_50_ values of compounds (**1**, **2**, **8**, and **11**) were in the range of 300–500 μM, while others were more than 500 μM (Table 2). Therefore, through the entire experiments, the maximum concentration of tested diterpenoid alkaloids was limited to 10 μM, a concentration about one-tenth to one-fiftieth of IC_50_ of them. In addition, optimum dosage of modeling agent (FFA) was also selected as the concentration of 0.5 mM through CCK-8 assay (Figure S77). 

### 2.3. The Inhibitory Effects of Isolated Compounds on Lipid Accumulation in FFA-Induced BRL Cells

#### 2.3.1. TG, TC, ALT, and AST Levels of Fifteen Isolated Compounds in FFA-Induced BRL Cells

**Figure 5:**
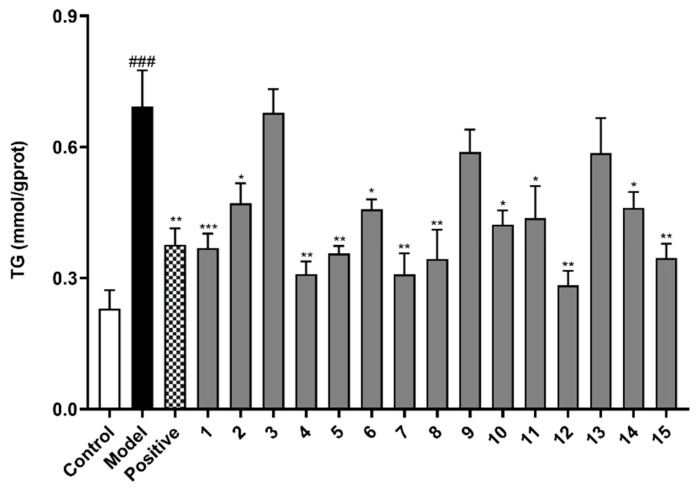
The effects of compounds (**1**–**15**) on triglyceride (TG) level in FFA-induced BRL cells. BRL cells were treated with a mixture of 0.5 mM FFA in the absence or presence of compounds (**1**–**15**) at a concentration of 10 µM. The values are presented as mean ± SEM of six independent experiments. ^###^ *p* < 0.001, vs. the control group; * *p* < 0.05, ** *p* < 0.01, *** *p* < 0.001, vs. the model group.

We selected FFA-induced BRL cells (oleate and palmitate ratio was 2:1) to establish a cellular NAFLD model, which is a widely used method by researchers for studying diseases related to hepatocytes lipid accumulation [18]. The TG secretion was measured to preliminarily evaluate the inhibitory effect on lipid accumulation of 15 isolated alkaloid compounds. The results show that TG content in the model group was 3 times that of the normal control group, indicating that BRL cell stimulated by 0.5 mM FFA for 24 h could successfully establish a hepatocytes model of lipid accumulation. Compounds **1**–**15** were tested at a concentration of 10 μM in FFA-induced BRL cells to determine their inhibitory effects on hepatocytes TG secretion (Figure 5). Atorvastatin calcium (ATC) is a statin cholesterol-lowering drug and widely used in the clinical treatment of hyperlipidemia and NAFLD, etc. [19,20]. Therefore, we used atorvastatin calcium (ATC, 10 µM) as a positive control drug. Compared with the model group, compounds **1**, **2**, **4**–**8**, **10**–**12**, **14,** and **15** had the effect of inhibiting the lipid accumulation (TG secretion) in BRL cells stimulated by FFA (*p* < 0.05), and compound **12** showed the strongest inhibition effect on the level of TG (*p* < 0.01). 

**Figure 6:**
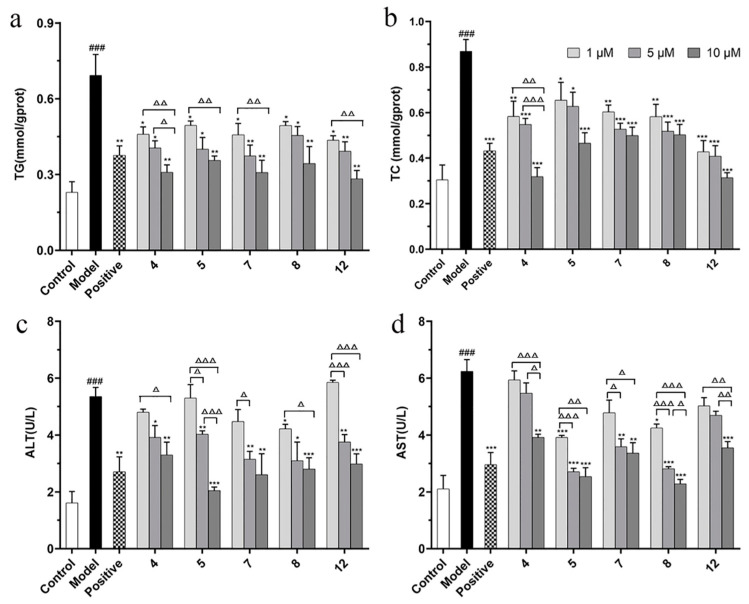
The effects of compounds (**4**, **5**, **7**, **8**, and **12**) on TG (**a**), TC (**b**), ALT (**c**), and AST (**d**) level in FFA-induced BRL cells. BRL cells were treated with a mixture of 0.5 mM FFA in the absence or presence of compounds (**4**, **5**, **7**, **8**, and **12**) at the concentration of 1, 5, or 10 µM. The values are presented as mean ± SEM of six independent experiments. ^###^ *p* < 0.001, vs. the control group; * *p* < 0.05, ** *p* < 0.01, *** *p* < 0.001, vs. the model group. ^Δ^ *p* < 0.05, ^ΔΔ^ *p* < 0.01, ^ΔΔΔ^ *p* < 0.001, pairwise comparisons between three doses of each compound group.

The activity of reducing lipid accumulation (TG secretion) of compounds **4**, **5**, **7**, **8**, and **12** was better than that of the positive control drug (10 µM). Therefore, we selected these five compounds for the further experiments. In addition to TG level, TC level and the liver injury indicators (ALT and AST) as typical indicators of NAFLD metabolic phenotype are also necessary for the five isolated compounds to be measured. According to the results, compounds **4**, **5**, **7**, **8**, and **12** ameliorated the indicators of TG, TC, ALT, and AST levels to varying degrees in FFA-induced BRL cells (Figure 6), showing that these five compounds have the potential to be developed as drugs for treatment of NAFLD. 

The significance across dosage was evaluated by performing multiple comparisons within each compound with the different concentrations compared with each other. The results show that the low (1 µM) and high (10 µM) doses of compound **4** were statistically significant at TG, TC, ALT, and ALT levels (^Δ^ *p* < 0.05), while the medium (5 µM) and high (10 µM) doses of compound **4** were statistically significant at TG, TC, and AST levels (^Δ^ *p* < 0.05). The low (1 µM) and high (10 µM) doses of compound **5** and **12** were statistically significant at TG, ALT, and ALT levels (^ΔΔ^ *p* < 0.01). The low (1 µM) and high (10 µM) doses of compound **7** were statistically significant at TG and AST levels (^Δ^ *p* < 0.05). In pairwise comparison between different dose groups, compound **5** had a significant difference in ALT level (^Δ^ *p* < 0.05), as did compound **8** in AST level (^Δ^ *p* < 0.05).

#### 2.3.2. Oil Red O Staining of Lipid Droplets and Quantitative Analysis in FFA-Induced BRL Cells

**Figure 7:**
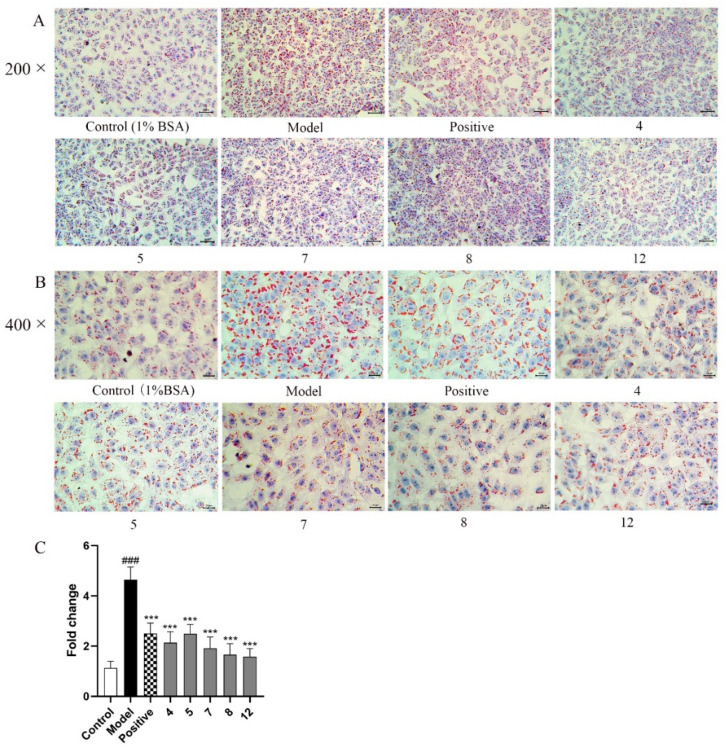
Oil red O staining of lipid droplets and quantitative analysis in BRL cells treated with or without 0.5 mM FFA mixed compounds **4**, **5**, **7**, **8,** and **12** (10 µM); (**A**) 200×; (**B**) 400×; (**C**) Quantitative analysis of red-stained cells. The images shown are representatives of 3 replicates of each. The values are presented as mean ± SEM. ^###^ *p* < 0.001, vs. the control group; *** *p* < 0.001, vs. the model group.

Combined with the oil red O staining and quantitative analysis assay, the effect of reducing lipid accumulation of compounds **4**, **5**, **7**, **8**, and **12** was performed in FFA-induced BRL cells. Oil red O staining showed that the above five compounds can significantly reduce lipid accumulation in FFA-induced BRL cells compared to the model group (Figure 7A,B). Quantitative analysis of lipid accumulation was also analyzed by ImageJ 1.8.0 software by calculating the area of red-stained cells (Figure 7C). Compared with the control group, the fold change values decreased significantly in FFA-stimulated cells, indicating that using 0.5 mM FFA as a modeling agent could successfully establish lipid accumulation in hepatocytes. Meanwhile, the fold change values on cells treated with compounds **4**, **5**, **7**, **8,** or **12** were less than the model group (*** *p* < 0.001), which was comparable with the positive group (ATC). The above experimental results show that compounds **4**, **5**, **7**, **8,** and **12** could significantly reduce lipid accumulation in FFA-induced BRL cells. Similarly, the above five compounds also had good inhibitory effects on lipid accumulation in FFA-induced HepG2 cells by oil red O evaluation and TG secretion assay (Figures S78 and S79).

### 2.4. Preliminary Mechanism Study of Five Isolated Compounds Based on Network Pharmacology Method

#### 2.4.1. Prediction of Underlying Mechanism for Five Isolated Compounds in the Treatment of NAFLD

**Figure 8:**
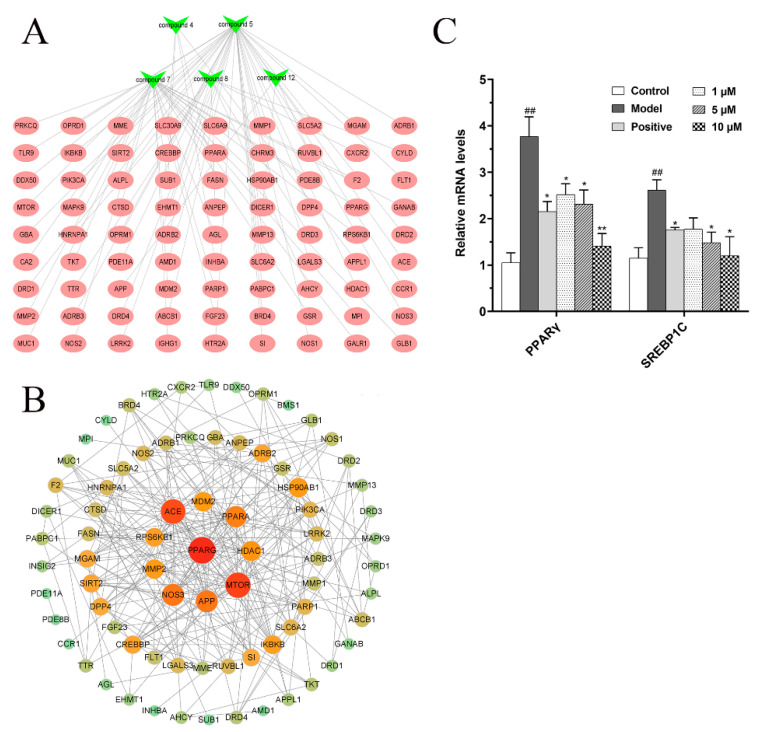
Underlying mechanism study of five isolated compounds based on network pharmacology method. (**A**) Component–target network diagram; (**B**) Protein interaction (PPI) network diagram. (**C**) The effects of compound **12** on fatty acid synthesis in FFA-induced BRL cells. ^##^ *p* < 0.01, vs. the control group; * *p* < 0.05, ** *p* < 0.01, vs. the model group.

According to the network pharmacology method, a total of 81 targets were screened, and these targets were considered as potential targets of five compounds for the treatment of NAFLD. At the same time, Cytoscape 3.8.0 software was used to construct a compounds–targets network diagram (Figure 8A). The protein interaction (PPI) network diagram (Figure 8B) shows that PPARG, MTOR, ACE, PPARA, NOS3, etc., play very important roles in protein interaction, indicating that these targets may be potential therapeutic targets. It is worth noting that PPARG has the strongest protein interaction among the screened targets, suggesting that the inhibition of lipid synthesis may be the underlying mechanism by which five compounds exert their effect on reducing lipid accumulation.

#### 2.4.2. Effects of Compound **12** on Fatty Acid Synthesis in FFA Mixture-Induced BRL Cells

Studies have shown that many enzymes involved in lipid synthesis are upregulated by lipogenic transcription factors, such as peroxisome proliferator-activated receptor gamma (PPARγ, also known PPARG) and sterol regulatory element-binding protein 1C (SREBP1C), which in turn lead to cellular lipid accumulation. Therefore, PPARγ and SREBP1C as the lipogenic regulators play an important role in the occurrence and development of NAFLD [21,22,23]. Compared with the other 14 compounds, compound **12** has the strongest inhibitory effect on TG level (*p* < 0.01). Therefore, we selected compound **12** as a representative for subsequent potential mechanism studies. Based on network pharmacology predicted results and studies in the literature, the relative mRNA levels of PPARγ and SREBP1C in FFA-induced BRL cells were analyzed by real-time PCR system. Our results show that compound **12** decreased relative mRNA levels of PPARγ and SREBP1C in a dose-dependent manner (Figure 8C). It indicated that compound **12**, as a representative of diterpenoid alkaloids isolated from *D. brunonianum*, may inhibit fat accumulation by regulating the expression of lipid synthesis transcription factors, such as PPARγ and SREBP1C.

## 3. Materials and Methods

### 3.1. General Experimental Procedures

The optical rotations were measured on an Autopol IV Automatic polarimeter (SiberHegner, Hong Kong, China). UV spectra were measured by a Shimadzu UV-2550 ultra-visible spectrophotometer (Shimadzu, Kyoto, Japan). IR spectra were recorded on EQUINOX55 FTIR spectrophotometer (Bruker, Bremerhaven, Germany). NMR spectra were obtained with Bruker AV 400 spectrometer (Bruker, Bremerhaven, Germany). HR-ESI-MS spectra were performed on a Triple-TOF 5600 mass spectrometer (AB Sciex, Redwood City, CA, USA). Silica gel (200–300 mesh, Qingdao Haiyang Chemical Co., Ltd., Qingdao, China) was used for column chromatography (CC). Sephadex LH-20 (25–100 μm, Fluka BioChemika, Buchs, Switzerland) and ODS RP-C18 silica gel (40–63 μm, Merck KgaA). The pre-coated silica gel GF254 plates (Qingdao Haiyang Chemical Co., Ltd., Qingdao, China) were used for TLC. Multimode Microplate Reader (PerkinElmer EnSpire, Waltham, MA, USA). Optical microscope (Mingmei Microscope, Guangzhou, China). StepOne Real-Time PCR System (Applied Biosystems, Foster City, CA, USA).

Atorvastatin calcium (product number A121956), sodium palmitate (product number S161450), and sodium oleate (product number S1O4196) were obtained from Shanghai Aladdin Biochemical Technology Co., Ltd., Shanghai, China. Bovine serum albumin (fatty acid free, product number A8850) was obtained from Beijing Solarbio Science & Technology Co., Ltd., Beijing, China. Dulbecco’s modified Eagle medium (Thermo Fisher Biochemicals Co., Ltd., Beijing, China). Fetal bovine serum (Biological Industries, Kibbutz Beit Haemek, USA). Penicillin/streptomycin (1%), oil red O, ematoxylin solution (Solarbio, Beijing, China). Cell Counting Kit-8 (Biosharp, Beijing, China). TG, TC, ALT, and AST reagent test kits (Nanjing Jiancheng Bioengineering Research Institute Co., Ltd., Nanjing, China). Trizol reagent (Ambion, Waltham, MA, USA). All solvents were of analytical grade.

### 3.2. Plant Material

The aerial parts of *D. brunonianum* Royle were collected from Linzhi (Tibet Autonomous Region, China) and were identified by Professor Ga Wu (Tibetan Traditional Medical College, Lasa, China). The voucher specimen was deposited at the Department of College of Chinese Medicine, Guangzhou University of Chinese Medicine, Guangzhou, China.

### 3.3. Extraction and Isolation

Dried and powdered aerial parts of *D. Brunonianum* Royle (20 kg) were repeatedly extracted with methanol (MeOH) at room temperature for five times, and each extraction time was for 24 h. The extracted solution was evaporated in vacuum to afford the crude methanol extraction. The concentrated extraction was suspended in water and partitioned in different solvents to obtain petroleum extract (650 g), chloroform extract (240 g), EtOAc extract (42 g), and n-butanol extract (400 g), respectively. 

The crude chloroform fraction was subjected to CC with silica gel (100–200 mesh) and eluted with cyclohexane (CYH)/ethyl acetate (EtOAc) gradient system (5:0 to 1:0) and EtOAc/MeOH gradient system (4:1 to 1:1); the gradient system gave fractions 1–9. Fr.5 was separated to Sephadex LH-20 CC (CHCl_3_/MeOH, 1:1) to afford subfractions (Fr.5-1 and Fr.5-2). Fr.5-2 was subjected to normal phase silica gel column with CYH/EtOAc gradient system (3:1 to 1:3), ODS middle pressure column eluted with MeOH/H_2_O (30–80%) to obtain compounds **5** (10 g), **6** (22 mg), **7** (1034 mg), **8** (2800 mg), **9** (254 mg). Fr. 6 was subjected to Sephadex LH-20 CC (CHCl_3_/MeOH, 1:1) to afford subfractions (Fr.6-1 and Fr.6-2). Fr.6-1 was purified by ODS middle pressure column eluted with MeOH/H_2_O (60%) to obtain **10** (93 mg), and Fr.6-1 was subjected to normal phase silica gel column CYH/EtOAc (1:5) to obtain **11** (110 mg). Fr.6-2 was subjected to normal phase silica gel column CYH/EtOAc (1:1) and ODS middle pressure column eluted with MeOH/H_2_O (40–80%) to obtain **12** (18 mg). Fr.7 was subjected to Sephadex LH-20 CC (CHCl_3_/MeOH, 1:1) and ODS reversed phase column (40–80%) to yield **13** (382 mg). Fr.8 was subjected to Sephadex LH-20 CC (CHCl_3_/MeOH, 1:1), and ODS reversed phase column (40–80%) to afford subfractions (Fr.8-1 and Fr.8-2). Fr.8-1 was subjected to silica gel column (CHCl_3_/MeOH, 10:1) to obtain **14** (15 mg) and **15** (80 mg). Fr.9 was subjected to silica gel column with CHCl_3_/MeOH gradient system (100:1 to 5:1), and preparative HPLC (acetonitrile-Water system) to obtain **1** (9 mg), **2** (25 mg), and **3** (6 mg).

The crude n-butanol extract (400 g) was subjected to normal phase silica CC eluted with CH_2_Cl_2_/MeOH gradient system (50:1 to 1:1) to provide Frs.1–9. Fr.3 (53 g) was subjected to silica gel CC with CH_2_Cl_2_/MeOH gradient system (1:0 to 2:1) to obtain (Fr.3.1-Fr.3.4). Fr.3.4 was eluted by Sephadex LH-20 column (80% MeOH/H_2_O), gradient elution by ODS CC (MeOH/H_2_O, 20–70%), and silica gel column chromatography eluted with EtOAc/MeOH gradient system (1:0 to 5:1) to obtain **4** (375 mg).

### 3.4. Characterization of Compounds ***1**–**4***

Brunodelphinine B (**1**): White amorphous powder; ${\left[\alpha \right]}_{D}^{25}$ +70.00 (c = 0.800, MeOH); UV(MeOH) *λ*_max_: 193, 208 nm; IR (KBr) *v*_max_: 3435, 2952, 2885, 2826, 1728, 1671, 1629, 1454, 1124, 1084, 744, 659 cm^−1^; CD (MeOH, Δ*ε*) *λ*_max_ 227 (−5.01), 310 (−0.55); HR-ESI-MS *m*/*z* 480.2592 [M + H]^+^ (calculated for C_25_H_38_NO_7_, 480.2597); ^1^H-NMR (MeOD, 400 MHz) and ^13^C-NMR (MeOD) data see Table 1.

Brunodelphinine C (**2**): Light yellow amorphous powder; ${\left[\alpha \right]}_{D}^{25}$ +4.35 (c = 0.850, MeOH); UV(MeOH) *λ*_max_: 200, 209, 245 nm; IR (KBr) *v*_max_: 3437, 2939, 2895, 1647,1462, 1196, 1088, 560 cm^−1^. CD (MeOH, Δ*ε*) *λ*_max_ 203 (+4.07), 225 (−0.30), 259 (−2.14) nm; HR-ESI-MS *m*/*z* 466.2445 [M + H]^+^ (calculated for C_24_H_36_NO_8_, 466.2441). ^1^H-NMR (CDCl_3_) and ^13^C-NMR (CDCl_3_) data see Table 1.

Brunodelphinine D (**3**): Light yellow amorphous powder; ${\left[\alpha \right]}_{D}^{25}$ −9.33 (c = 0.300, MeOH); UV(MeOH) *λ*_max_: 200, 209, 257 nm; IR (KBr) *v*_max_: 3447, 2949, 2894, 2827, 1697, 1627, 1585, 1464, 1200, 1126, and 1089 cm^−1^. CD (MeOH, Δ*ε*) *λ*_max_ 203 (+4.07), 225 (−0.30), 259 (−2.14) nm; HR-ESI-MS *m*/*z* 454.2439 [M + H]^+^ (calculated for C_23_H_36_NO_8_, 454.2446). ^1^H-NMR (MeOD) and ^13^C-NMR (MeOD) data are shown in Table 1.

Brunodelphinine E (**4**): White amorphous powder; ${\left[\alpha \right]}_{D}^{25}$ +46.0 (c = 0.10, MeOH); UV(MeOH) *λ*_max_: 282 nm; IR (KBr) *v*_max_: 3410, 2932, 2872, 1594, 1459, 1384, 1079 cm^−1^; HR-ESI-MS *m*/*z* 418.2586 [M + H]^+^ (calculated for C_24_H_36_NO_5_, 418.2543); ^1^H-NMR (DMSO) and ^13^C-NMR (DMSO) data see Table 1. 

### 3.5. Effects of Fifteen Isolated Compounds on Cell Activity

Buffalo rat liver (BRL) cells were obtained from the Cell Bank, Committee for Typical Culture Preservation, Chinese Academy of Sciences. BRL cells were cultured in DMEM (containing 10% fetal bovine serum and 1% penicillin/streptomycin) under humidified 5% CO_2_ (*v*/*v*) atmosphere at 37 °C. To determine the cell viability of compounds **1**–**15**, the CCK-8 assay was performed [24]. The fifteen isolated compounds were dissolved in DMSO and diluted 1:1000 in culture medium, respectively. Cells were seeded in 96-well plates (6 × 10^3^ cells per well) for 24 h incubation and then treatment with different concentrations (1, 10, 20, 50, 100, 200, and 500 µM) of each compound for 24 h or 48 h, respectively. CCK-8 solution (10% of the total solution) was added to each well, further incubated for 1 h at 37 °C. The absorbance was measured at 450 nm using a Multimode Microplate Reader.

### 3.6. Effects of Fifteen Isolated Compounds on the Inhibition of Lipid Accumulation in FFA-Induced BRL Cells

#### 3.6.1. TG, TC, ALT, and AST Quantification of Fifteen Isolated Compounds in FFA-Induced BRL Cells

BRL cells were grown in 24-well plates until 70–80% confluence. Subsequently, 0.5 mM FFA (oleate and palmitate in a final ratio of 2:1) was applied for 24 h to establish an in vitro model of hepatocytes lipid accumulation in the model group, treated with 0.5 mM FFA combination with 1, 5, or 10 µM isolated compounds for 24 h in alkaloids groups. A portion of 1% bovine serum albumin (BSA) was added in BRL cells of the control group. Then, the medium of each group was collected for determination of ALT and AST levels by commercial kits. After removing the medium, 200 µL of 0.25% trypsin was added to digest the cells in 37 °C for 2 min. Then, 500 µL of DMEM that contained 10% fetal bovine serum (FBS) was added to stop digesting. Cells suspension was centrifuged at 800 r/min for 3 min. After adding 200 µL of ethanol, cells were collected and crushed by ultrasonic cell disruptor at 4 °C. Part of the cell fragmentation solution was used for the determination of cell protein content, the other part was used to determine TG and TC levels by commercial kits according to the manufacturer’s instruction [25]. 

#### 3.6.2. Oil Red O Staining Assay

BRL cells were plated in 96-well plates until reaching 70–80% confluence and treated with 0.5 mM FFA mixture and the tested compounds for 24 h. Controls were incubated for the same period in complete medium. 

After 24 h of incubation, the cells were fixed with 5% formalin solution for 30 min and then incubated with oil red O working solution for 15 min at room temperature. After that, the cells were washed once with 60% isopropanol and three times with water. The nuclei were stained with hematoxylin solution for 2 min at room temperature. Then, the operation of oil red O staining was referred to the previous research [26]. Finally, the red oil droplets were observed using an optical microscope.

### 3.7. Underlying Mechanism Study of Five Isolated Compounds Based on Network Pharmacology

#### 3.7.1. Prediction of Potential Targets of Action for Five Isolated Compounds in the Treatment of NAFLD

The ChemDraw software (14.0) was used to draw the structural formulas of the five compounds (**4**, **5**, **7**, **8**, and **12**), and their respective SMILE formulas were saved. The pharmmapper database (http://www.lilab-ecust.cn/pharmmapper/ (accessed on 14 February 2022)) and swisstarget database (http://swisstargetprediction.ch/ (accessed on 14 February 2022)) were used to predict the potential targets of five compounds. All targets were aggregated, and duplicate targets were removed to obtain potential targets for five compounds. In OMIM (https://www.omim.org/ (accessed on 14 February 2022)), TTD (http://db.idrblab.net/ttd/ (accessed on 15 February 2022)), GeneCards (https://www.genecards.org/ (accessed on 15 February 2022)), DisGeNET (https://www.disgenet.org/dbinfo (accessed on 15 February 2022)) and DrugBank (https://go.drugbank.com/drugs (accessed on 15 February 2022)) databases, with “Non-alcoholic liver disease” as the search term, NAFLD-related targets were searched. The predicted targets of the five compounds were intersected with the related targets of NAFLD, and the intersection genes were considered as possible targets for the treatment of NAFLD by the five compounds. 

#### 3.7.2. Target Protein Interaction (PPI) Core Network Construction

The potential therapeutic targets were imported to the String (https://string-db.org/ (accessed on 16 February 2022)) database, and the multiple proteins tool was used to define the species as “Homo sapiens” to obtain protein interaction relationships. The results were imported into Cytoscape 3.8.0 software in CSV format for visual analysis, and a protein interaction network diagram was constructed. 

#### 3.7.3. Effects of Compound **12** on Fatty Acid Synthesis in FFA Mixture-Induced BRL Cells

Total RNA was extracted from BRL cells with the use of the Trizol reagent. The expression levels of PPARγ and SREBP1C mRNA in FFA-induced BRL cells were analyzed by Applied Biosystems StepOne Real-Time PCR (RT-PCR) System. In addition, the PCR amplification was performed for 40 repetitive thermal cycles with SYBR green (95 °C for 15 s, 60 °C for 15 s, and 72 °C for 32 s). Data were normalized by the amount of β-actin mRNA. Primers are listed in Table 3.

### 3.8. Statistical Analysis

All data are expressed as mean ± SEM of six independent experiments. SPSS 23.0 software was used for statistical analysis by one-way ANOVA method. A *p* value less than 0.05 was considered statistically significant.

## 4. Conclusions

Natural products extracted from medical plants are a rich source of biologically active substances, which play an important and irreplaceable role in the drug discovery field [27,28,29]. In our present research, fifteen compounds were isolated from the CHCl_3_ extraction of *D. brunonianum*, including four undescribed compounds (**1**–**4**) and eleven known compounds (**5**–**15**). Among the known compounds, uraphine (**10**) was isolated from genus *Delphinium* for the first time, and delpheline (**6**), anthranoyllycoctonine (**11**), sharwuphinine A (**12**), shawurensine (**14**), and delavaine B (**15**) were first discovered in *D. brunonianum*. The inhibitory effects of isolated compounds (**1–15**) on hepatocytes lipid accumulation were also evaluated in FFA-induced BRL cells. Our results indicate that five selected compounds (**4**, **5**, **7**, **8**, and **12**) showed strong inhibitory effects on hepatocytes lipid accumulation in a dose-dependent manner (1, 5, and 10 µM). Compound **12**, as a representative of diterpenoid alkaloids isolated from *D. brunonianum*, may inhibit fat accumulation by regulating the expression of lipid synthesis transcription factors PPARγ and SREBP1C. In summary, the compounds isolated in this study could serve as a potential for treatment of diseases caused by lipid accumulation, such as NAFLD and hyperlipidemia diseases. In addition, this study enriches the chemical constituents and activity of *D. brunonianum* and provides a reference for further research on the potential mechanism of diterpenoid alkaloids in improving hepatocytes lipid accumulation. 

## Figures and Tables

**Table 1:** NMR spectroscopic data of brunodelphinine B–E (**1**–**4)** (400 MHz for ^1^H-NMR and 100 MHz for ^13^C-NMR).

Position	Brunodelphinine B (1), MeOD		Brunodelphinine C (2), CDCl_3_		Brunodelphinine D (3), MeOD		Brunodelphinine E (4), DMSO	
	*δ_C_*, Type	*δ*_H_ (*J* in Hz)	*δ_C_*, Type	*δ*_H_ (*J* in Hz)	*δ_C_*, Type	*δ*_H_ (*J* in Hz)	*δ_C_*, Type	*δ*_H_ (*J* in Hz)
1	89.6, CH	3.82 *m*	80.2, CH	3.26 *s*	91.9, CH	3.25 *q* (4.6)	34.8, CH_2_	1.9 m
								1.46 *d* (9.7)
2	129.6, CH	5.98 *dd* (9.7, 1.4)	24.4, CH_2_	1.85 *m*	23.7, CH_2_	2.09 *m*	18.3, CH_2_	1.22 *dd* (8.0, 4.0)
				1.48 *m*	32.9, CH_2_	1.04 *m*	-	1.52 *d* (16.3)
3	148.8, CH	7.02 *tt* (19.7, 7.3)	28.4, CH_2_	1.76 *m*		1.88 *m*	36.3, CH_2_	1.78 *d* (13.3)
				1.59 *m*		1.60 *m*		1.22 *dd* (8.0, 4.0)
4	40.7, C		44.5, C		39.7, C	-	36.3, C	-
5	38.3, CH	2.95 *dd* (10.8, 6.6)	48.4, CH	1.76 *s*	49.8, CH	2.22 *s*	44.7, CH	1.42 *d* (8.6)
6	202.2, C	-	89.2, CH	3.63 *d* (1.3)	88.5, CH	3.99 *t* (6.6)	18.9, CH_2_	1.09 *d* (13.4)
								1.52 *d* (16.3)
7	77.1, CH	4.23 *s*	90.4, C	-	209.3, C	-	67.2, CH	3.76 *dd* (11.6, 4.1)
8	45.5, CH	2.04 *s*	80.9, C	-	74.4, C	-	42.2, C	-
9	39.4, CH	3.31 *m*	41.9, CH	3.59 *t* (5.4)	41.6, CH	3.63 *s*	39.4, CH	2.03 *t* (8.6)
10	56.1, CH	2.87 *dd* (9.7, 5.0)	47.1, CH	2.08 *m*	45.3, CH	2.67 *m*	44.9, C	
11	50.8, C	-	49.5, C	-	46.4, C	-	27.5, CH_2_	1.70 *d* (5.4)
12	27.7, CH_2_	2.16 *m*	26.5, CH_2_	1.87 *m*	26.9, CH_2_	1.50 *dd* (15.3, 7.9)	35.5, CH_2_	2.33 *m*
		1.72 *m*		1.65 *m*		2.16 *dd* (15.3, 7.9)		-
13	21.8, CH_2_	2.06 *m*	35.6, CH	2.42 *s*	37.0, CH	2.54 *m*	24.8, CH_2_	1.60 *d* (14.9)
		1.35 *m*						1.38 *d* (8.6)
14	174.6, C	-	73.9, CH	3.99 *d* (5.5)	76.0, CH	4.13 *t* (4.5)	27.8, CH_2_	1.15 *dd* (21.9, 9.2)
15	27.9, CH_2_	2.20 *m*	31.4, CH_2_	2.40 *m*	32.6, CH_2_	1.64 *d* (16.8)	69.5, CH	4.02 *s*
		1.32 *m*		1.89 *m*		2.37 *d* (16.8)		
16	76.88, CH	3.30 *m*	81.3, CH	3.51 *m*	81.1, CH	3.61 *s*	155.8, C	-
17	48.7, CH_2_	3.28 *m*	77.7, CH	4.26 *d* (2.3)	62.4, CH_2_	3.82 *d* (17.3)	109.5, CH_2_	5.00 *s*
		-				3.51 *d* (17.3)		4.96 *s*
18	67.9, CH_2_	3.67 *m*	74.3, CH_2_	3.26 *s*	76.7, CH_2_	3.50 *s*	25.0, CH_3_	0.96 *s*
				3.43 *d* (9.1)				
19	170.2, C	-	138.0, CH	6.78 *d* (1.6)	142.5, CH	7.25 *s*	66.1, CH	4.37 *d* (7.5)
20		-	-	-	-	-	173.2, CH	8.60 *s*
21	42.7, CH_2_	3.67 *d* (3.3)	-	-	-	-	58.0, CH_2_	3.98 *d* (9.2)
		3.32 *d* (3.3)						3.66 *s*
22	11.1, CH_3_	1.20 *t* (7.2)	-	-	-	-	61.3, CH_2_	4.21 *d* (13.6)
								4.08 *m*
23	-	-	-	-	-	-	36.2, CH_2_	2.45 *d* (16.6)
								2.33 *m*
24	-	-	-	-	-	-	182.9, C	-
1-OCH_3_	56.0	3.23 *s*	56.0	3.18 *s*	55.5	3.26 *s*	-	-
6-OCH_3_	-	-	58.9	3.33 *d* (2.7)	57.4	3.37 *s*	-	-
7-OCH_3_	59.0	3.61 *s*	-	-	-	-	-	-
16-OCH_3_	52.6	3.0 *s*	56.7	3.31 *d* (2.9)	55.6	3.37 *s*	-	-
18-OCH_3_	51.2	3.63 *s*	59.3	3.29 *d* (3.2)	58.2	3.34 *s*	-	-
O-CH_2_-O	-	-	95.0	5.18 *d* (4.9)	-	-	-	-

Chemical shifts in *δ* (ppm); Down-field from TMS coupling constants *J* in Hz (s: singlet, d: doublet, t: triplet, q: multiplet).

**Table 2:** The IC_50_ value of compounds **1**–**15** and positive (ATC) in BRL cells.

Compounds	IC_50_ (μM)	Compounds	IC_50_ (μM)
**1**	387.8	**9**	>500.0
**2**	314.7	**10**	>500.0
**3**	>500.0	**11**	399.1.0
**4**	>500.0	**12**	>500.0
**5**	>500.0	**13**	>500.0
**6**	>500.0	**14**	>500.0
**7**	>500.0	**15**	>500.0
**8**	407.9	positive (ATC)	88.4

**Table 3:** Primer sequences of target genes for RT-PCR.

Genes	Forward Primer	Reverse Primer
β-actin	GCTTCTAGGCGGACTGTTAC	CCATGCCAATGTTGTCTCTT
PPARγ	CAAGGTGCTCCAGAAGATGA	GTGGGACTTTCCTGCTAATACA
SREBP1C	CAGCTGATTGCTATCTTTCC	TATGAGCCATGAGATCAGAG

## Data Availability

The data presented in this study are available in this article.

## Supplementary Materials

The following supporting information can be downloaded at: https://www.mdpi.com/article/10.3390/molecules27072257/s1, HR-ESI-MS, NMR, IR, UV, and CD spectra of **1**–**4** (Figure S1–S43); HR-ESI-MS, ^1^H and ^13^C NMR spectra of **5**–**15** (Figure S44–S76); Cell viability at different FFA concentrations in BRL cells (Figure S77); Effects of compounds (**4**, **5**, **7**, **8**, and **12**) on TG levels in FFA-induced HepG2 cells (Figure S78); Oil red O staining of lipid droplets and quantitative analysis in HepG2 cells (Figure S79).

## Sample Availability

Samples of the compounds are not available from the authors.

## Abbreviations

High Resolution Electrospray Ionization Mass Spectrometry, HR-ESI-MS; Nuclear Magnetic Resonance, NMR; Infrared Spectrometry, IR; Ultraviolet Visible Spectrometry, UV; Circular Dichroism Spectrometry, CD; Distortionless Enhancement by Polarization Transfer, DEPT; Two Dimensional ^1^H Correlation Spectroscopy, ^1^H-^1^H COSY; ^1^H-Detected Heteronuclear Multiple Bond Correlation, HMBC; ^1^H-Detected Heteronuclear Single Quantum Correlation, HSQC; Nuclear Overhauser Effect Spectroscopy, NOESY; High Performance Liquid Chromatography, HPLC; Thin Layer Chromatography, TLC; Crude Chloroform, CC; Octadecylsilyl, ODS; Real-Time PCR, RT-PCR; Nonalcoholic Fatty Liver Disease, NAFLD; Atorvastatin Calcium, ATC; Triglyceride, TG; Total Cholesterol, TC; Alanine Transaminase, ALT; Aspartate Transaminase, AST; Dulbecco’s Modified Eagle Medium, DMEM; Fetal Bovine Serum, FBS; Bovine Serum Albumin, BSA; Free Fatty Acids, FFA; Cell Counting Kit-8, CCK-8; Deuterated Chloroform, CDCl_3_; Methanlol-D4, MeOD; Dimethyl Sulfoxide, DMSO; Oak Ridge Thermal Ellipsoid Plot, ORTEP; Cyclohexane, CYH; Ethyl Acetate, EtOAc; Methyl Alcohol, MeOH; Dichloromethane, CHCl_2_; Trichloromethane, CHCl_3_; Methanol, MeOH; Buffalo Rat Liver, BRL.
