# Supplementary material for: Diterpenoid Alkaloids Isolated from Delphinium brunonianum and Their Inhibitory Effects on Hepatocytes Lipid Accumulation
Source: Molecules. 2022 Mar 30;27(7):2257. doi: 10.3390/molecules27072257 (PMC9000738; doi:10.3390/molecules27072257)
Supplement: Supplementary file 1 [file molecules-27-02257-s001.zip › molecules-1641769-supplementary.pdf]

Article

Diterpenoid Alkaloids Isolated from *Delphinium brunonianum* and Their Inhibitory Effects on Hepatocytes Lipid Accumulation

Huanhuan Ma [1,†], Yunxia Ma [1,†], Zeren Dawa [2,*], Yufeng Yao [1], Meiqi Wang [1], Kaihui Zhang [1], Chenchen Zhu [1,*], Fangle Liu [3,*] and Chaozhan Lin [1,*]

1 School of Pharmaceutical Sciences, Guangzhou University of Chinese Medicine, Guangzhou 510006, China; 20202110140@stu.gzucm.edu.cn (H.M.); 20191112282@stu.gzucm.edu.cn (Y.M.); 20193112173@stu.gzucm.edu.cn (Y.Y.); wangmeiqi@gzucm.edu.cn (M.W.); 20212110028@stu.gzucm.edu.cn (K.Z.)
2 Institute of Tibetan Medicine, University of Tibetan Medicine, Lasa 850000, China
3 School of Basic Medical Sciences, Guangzhou University of Chinese Medicine, Guangzhou 510006, China
* Correspondence: bairizerendawa@163.com (Z.D.); zhucc@gzucm.edu.cn (C.Z.); liufangle@gzucm.edu.cn (F.L.); linchaozhan@gzucm.edu.cn (C.L.)
† These authors contributed equally to this work.

Table of Contents:

Figure S1. HR-ESI-MS spectrum of brunodelphinine B (**1**).
Figure S2. UV spectrum of brunodelphinine B (**1**).
Figure S3. CD spectrum of brunodelphinine B (**1**).
Figure S4. IR spectrum of brunodelphinine B (**1**).
Figure S5. ^{1}H NMR spectrum of brunodelphinine B (**1**).
Figure S6. ^{1}C NMR spectrum of brunodelphinine B (**1**).
Figure S7. DEPT-135 spectrum of brunodelphinine B (**1**).
Figure S8. ^{1}H,^{1}H-COSY spectrum of brunodelphinine B (**1**).
Figure S9. HSQC spectrum of brunodelphinine B (**1**) .
Figure S10. HMBC spectrum of brunodelphinine B (**1**).
Figure S11. NOESY spectrum of brunodelphinine B (**1**) .
Figure S12. HR-ESI-MS spectrum of brunodelphinine C (**2**).
Figure S13. UV spectrum of brunodelphinine C (**2**) .
Figure S14. CD spectrum of brunodelphinine C (**2**) .
Figure S15. IR spectrum of brunodelphinine C (**2**) .
Figure S16. ^{1}H NMR spectrum of brunodelphinine C (**2**).
Figure S17. ^{1}C NMR spectrum of 2 brunodelphinine C (**2**).
Figure S18. DEPT-135 spectrum of brunodelphinine C (**2**) .
Figure S19. ^{1}H,^{1}H-COSY spectrum of brunodelphinine C (**2**).
Figure S20. HSQC spectrum of brunodelphinine C (**2**).
Figure S21. HMBC spectrum of brunodelphinine C (**2**).
Figure S22. NOESY spectrum of brunodelphinine C (**2**).
Figure S23. HR-ESI-MS spectrum of brunodelphinine D (**3**).
Figure S24. UV spectrum of brunodelphinine D (**3**) .
Figure S25. CD spectrum of brunodelphinine D (**3**) .
Figure S26. IR spectrum of brunodelphinine D (**3**) .
Figure S27. ^{1}H NMR spectrum of brunodelphinine D (**3**).

Figure S28. ^{1}C NMR spectrum of brunodelphinine D (**3**).
Figure S29. DEPT-135 spectrum of brunodelphinine D (**3**).
Figure S30. ^{1}H,^{1}H-COSY spectrum of brunodelphinine D (**3**).
Figure S31. HSQC spectrum of brunodelphinine D (**3**) **.**
Figure S32. HMBC spectrum of brunodelphinine D (**3**) **.**
Figure S33. NOESY spectrum of brunodelphinine D (**3**) .
Figure S34. HR-ESI-MS spectrum of brunodelphinine E (**4**).
Figure S35. UV spectrum of brunodelphinine E (**4**) **.**
Figure S36. IR spectrum of brunodelphinine E (**4**) .
Figure S37. ^{1}H NMR spectrum of brunodelphinine E (**4**).
Figure S38. ^{1}C NMR spectrum of brunodelphinine E (**4**) .
Figure S39. DEPT-135 spectrum of brunodelphinine E (**4**).
Figure S40. ^{1}H,^{1}H-COSY spectrum of brunodelphinine E (**4**) **.**
Figure S41. HSQC spectrum of brunodelphinine E (**4**) .
Figure S42. HMBC spectrum of brunodelphinine E (**4**) **.**
Figure S43. NOESY spectrum of brunodelphinine E (**4**) .
Figure S44. HR-ESI-MS spectrum of delbruline (**5**) **.**
Figure S45. ^{1}H NMR spectrum of delbruline (**5**) .
Figure S46. ^{1}C NMR spectrum of delbruline (**5**) **.**
Figure S47. HR-ESI-MS spectrum of delpheline (**6**) .
Figure S48. ^{1}H NMR spectrum of delpheline (**6**) .
Figure S49. ^{1}C NMR spectrum of delpheline (**6**) .
Figure S50. HR-ESI-MS spectrum of lycoctonine (**7**) **.**
Figure S51. ^{1}H NMR spectrum of lycoctonine (**7**) .
Figure S52. ^{1}C NMR spectrum of lycoctonine (**7**).
Figure S53. HR-ESI-MS spectrum of delbrunine (**8**) .
Figure S54. ^{1}H NMR spectrum of delbrunine (**8**).
Figure S55. ^{1}C NMR spectrum of delbrunine (**8**) .
Figure S56. HR-ESI-MS spectrum of delcosine (**9**) .
Figure S57. ^{1}H NMR spectrum of delcosine (**9**) **.**
Figure S58. ^{1}C NMR spectrum of delcosine (**9**) .
Figure S59. HR-ESI-MS spectrum of uraphine (**10**) .
Figure S60. ^{1}H NMR spectrum of uraphine (**10**) **.**
Figure S61. ^{1}C NMR spectrum of uraphine (**10**) .
Figure S62. HR-ESI-MS spectrum of anthranoyllycoctonine (**11**).
Figure S63. ^{1}H NMR spectrum of anthranoyllycoctonine (**11**).
Figure S64. ^{1}C NMR spectrum of anthranoyllycoctonine (**11**).
Figure S65. HR-ESI-MS spectrum of sharwuphinine A (**12**).
Figure S66. ^{1}H NMR spectrum of sharwuphinine A (**12**).
Figure S67. ^{1}C NMR spectrum of sharwuphinine A (**12**) .
Figure S68. HR-ESI-MS spectrum of browniine (**13**) .
Figure S69. ^{1}H NMR spectrum of browniine (**13**) .
Figure S70. ^{1}C NMR spectrum of browniine (**13**) .
Figure S71. HR-ESI-MS spectrum of shawurensine (**14**) .

Figure S72. ^{1}H NMR spectrum of shawurensine (**14**).
Figure S73. ^{1}C NMR spectrum of shawurensine (**14**).
Figure S74. HR-ESI-MS spectrum of delavaine B (**15**).
Figure S75. ^{1}H NMR spectrum of delavaine B (**15**) .
Figure S76. ^{1}C NMR spectrum of delavaine B (**15**) .
Figure S77. Cell viability at different FFA concentrations in BRL cells .
Figure S78. Effects of compounds (4, 5, 7, 8, and 12) on TG levels in FFA- induced HepG2 cells.
Figure S79. Oil red O staining of lipid droplets and quantitative analysis in HepG2 cells .

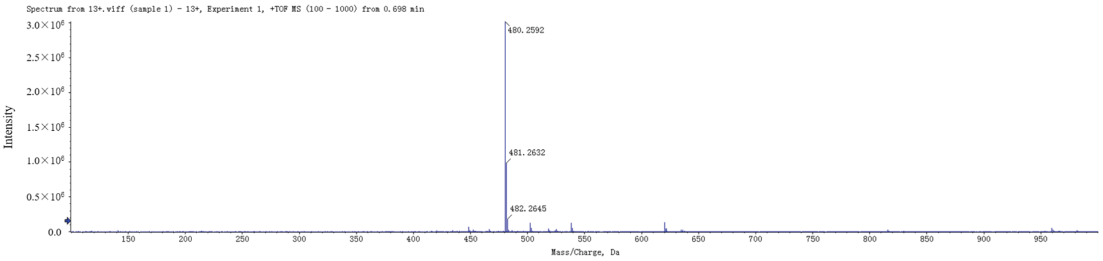

Figure S1. HR-ESI-MS spectrum of brunodelphinine B (**1**).

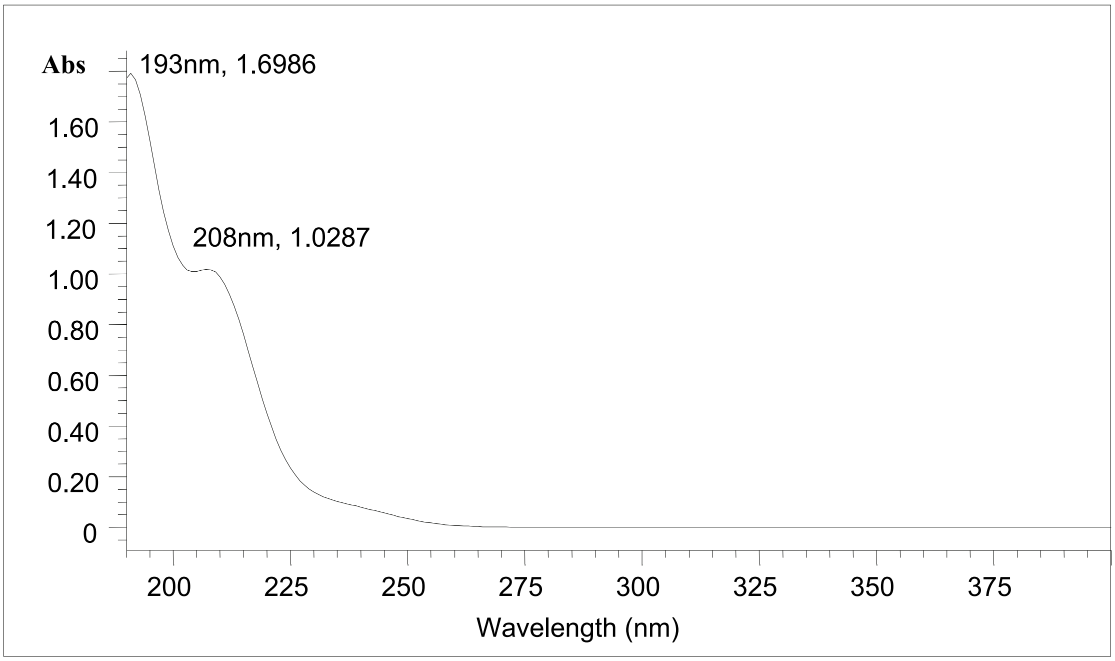

Figure S2. UV spectrum of brunodelphinine B (**1**).

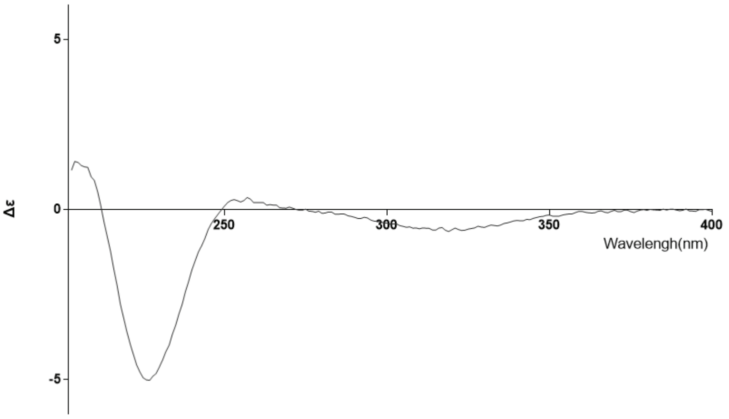

Figure S3. CD spectrum of brunodelphinine B (**1**).

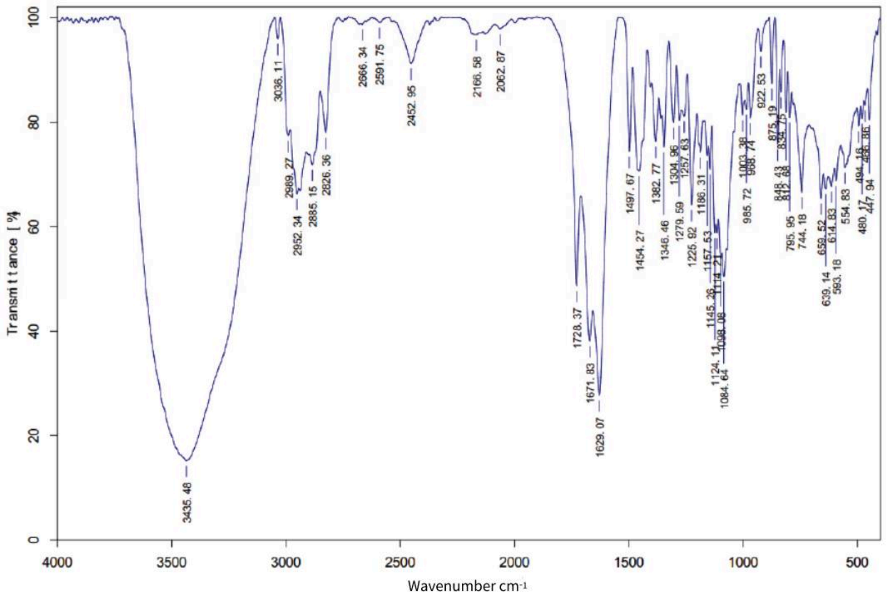

Figure S4. IR spectrum of brunodelphinine B (**1**).

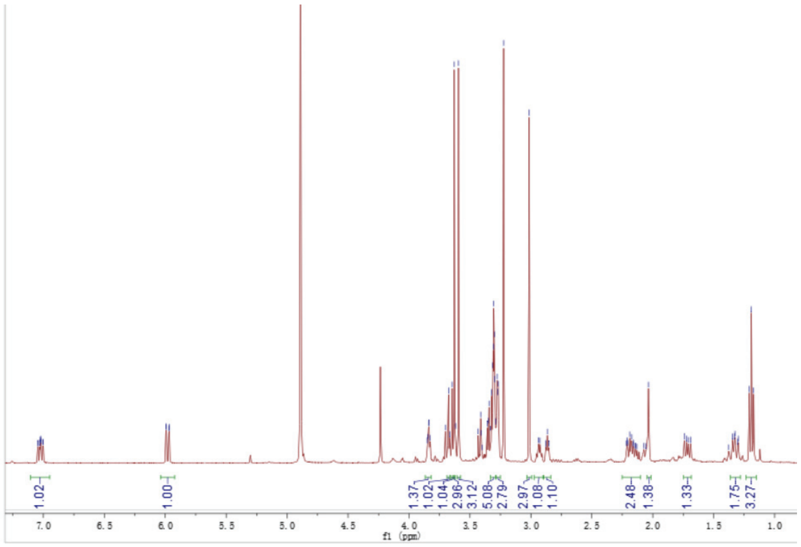

Figure S5. ^{1}H NMR spectrum of brunodelphinine B (**1**) (400 MHZ).

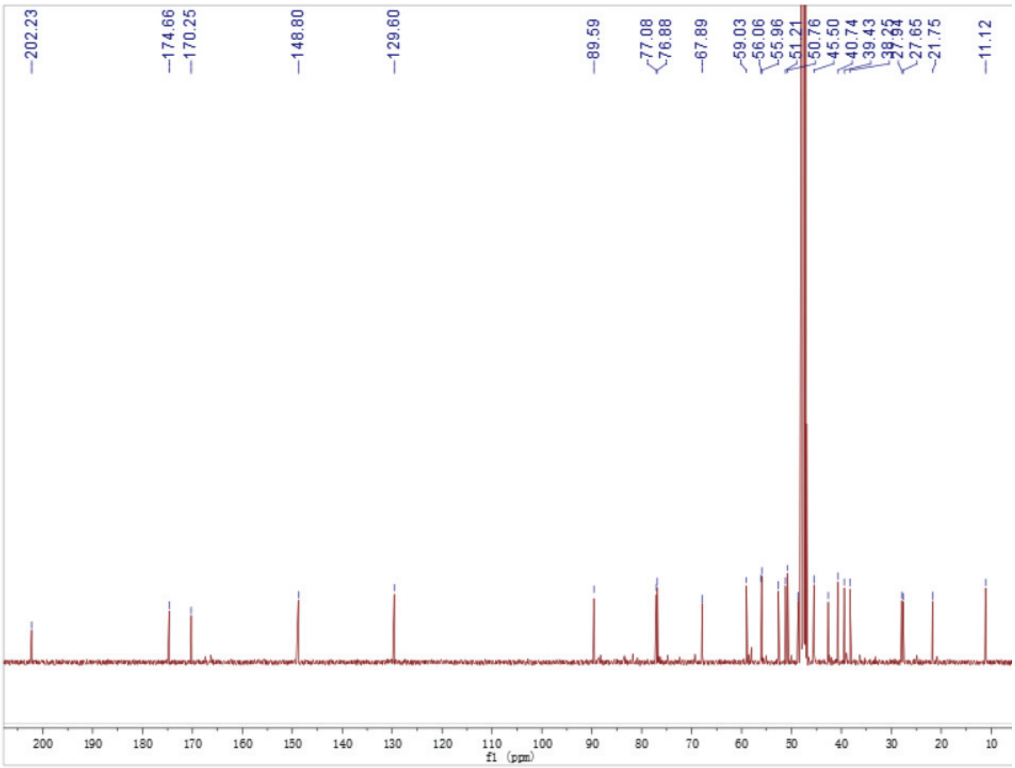

Figure S6. ^{1}C NMR spectrum of brunodelphinine B (**1**) (100 MHZ).

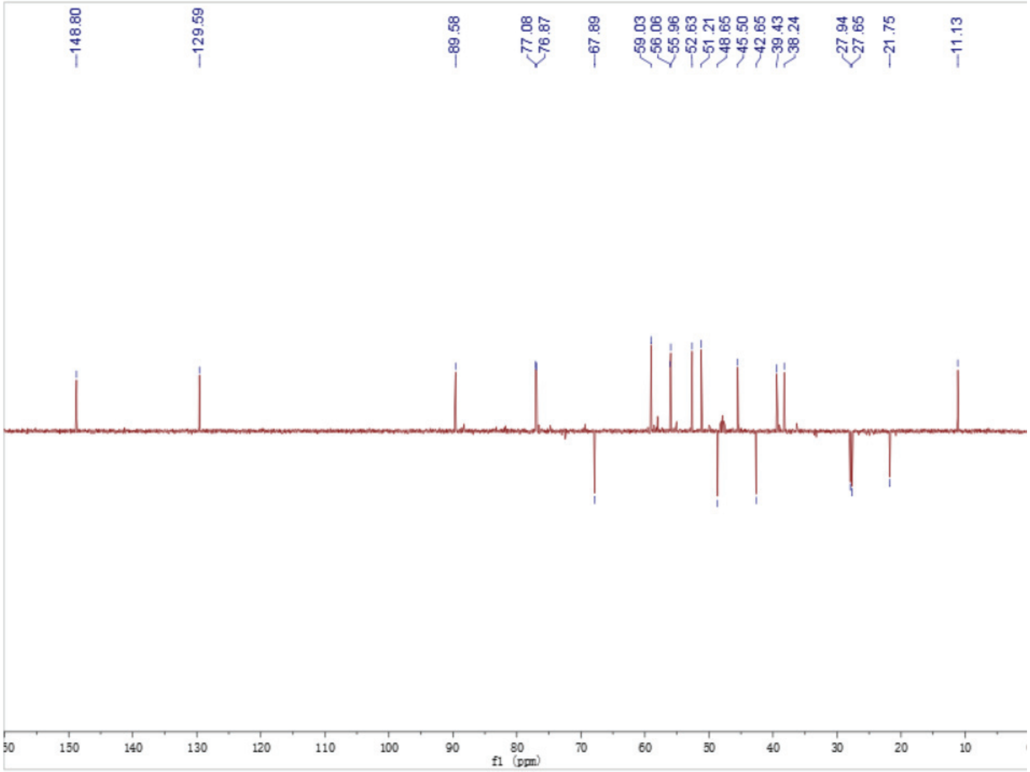

Figure S7. DEPT-135 spectrum of brunodelphinine B (**1**).

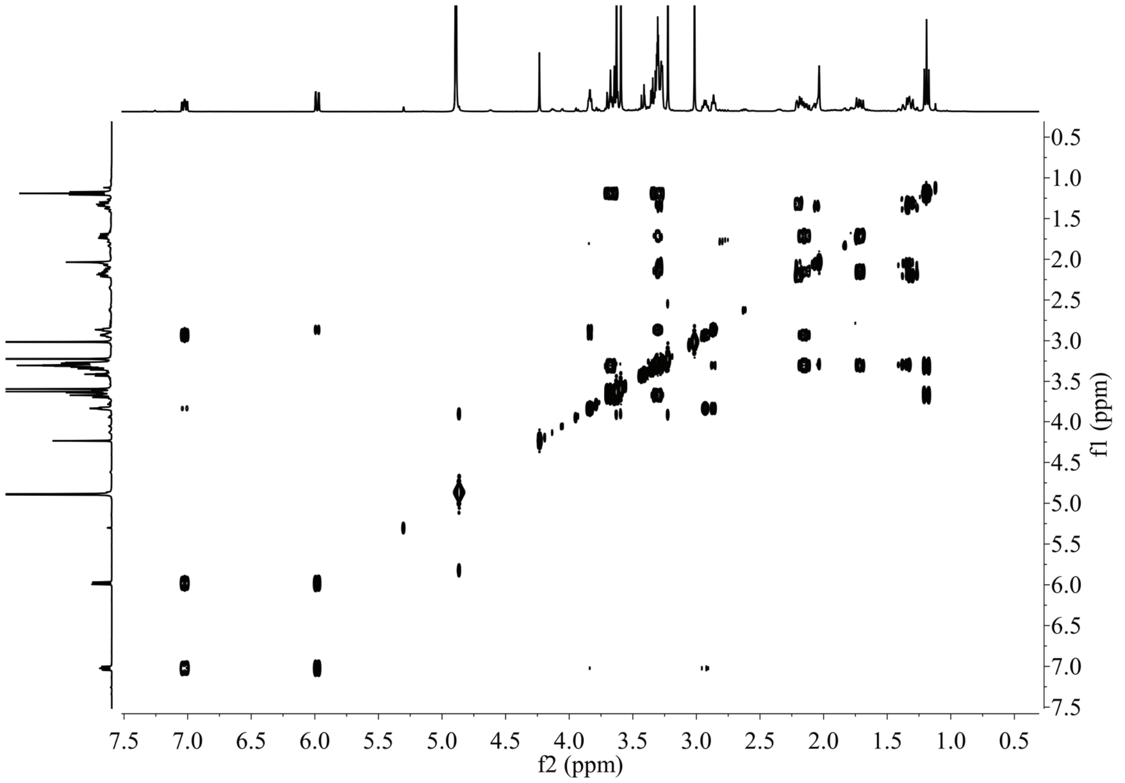

Figure S8. $^{1}H,^{1}H$-COSY spectrum of brunodelphinine B (**1**).

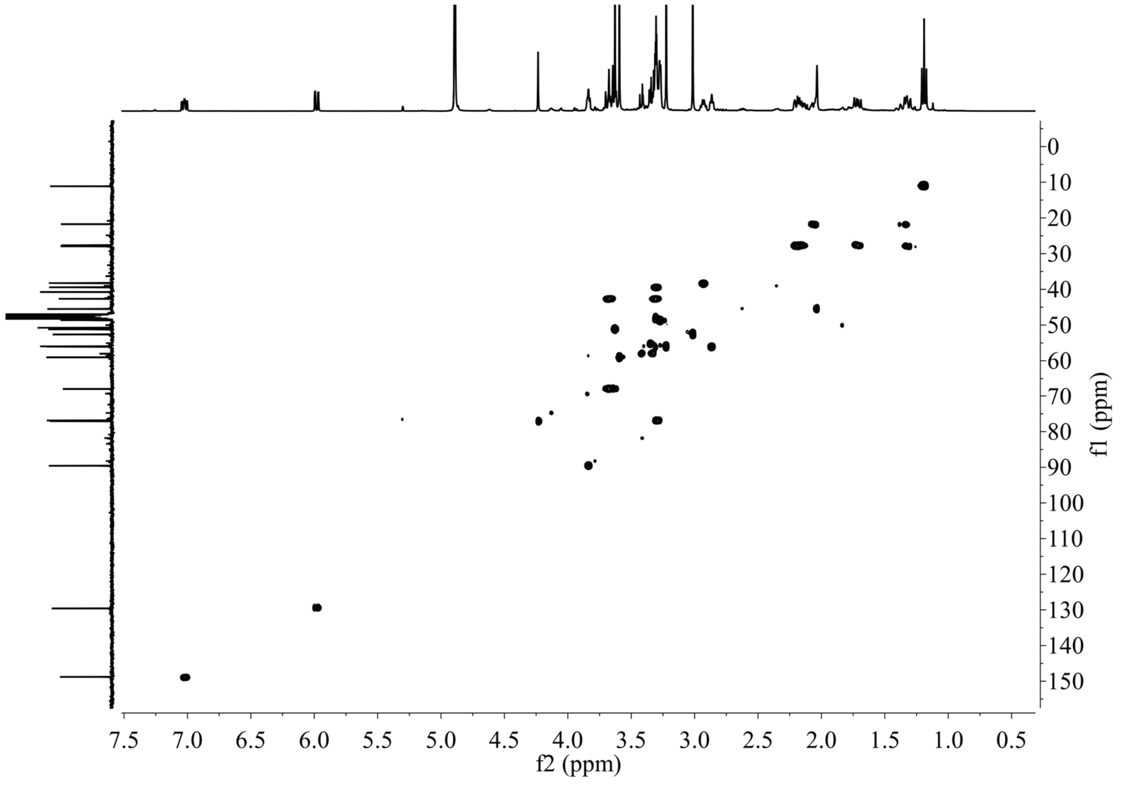

Figure S9. HSQC spectrum of brunodelphinine B (**1**).

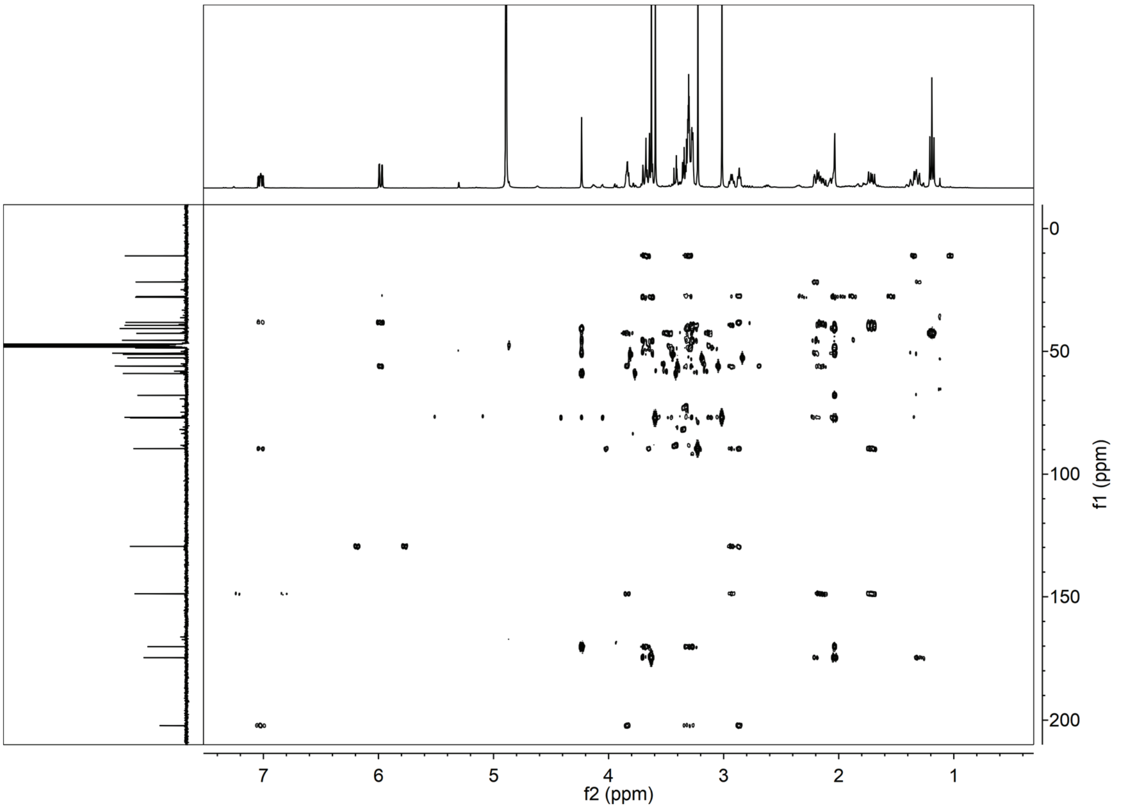

Figure S10. HMBC spectrum of brunodelphinine B (**1**).

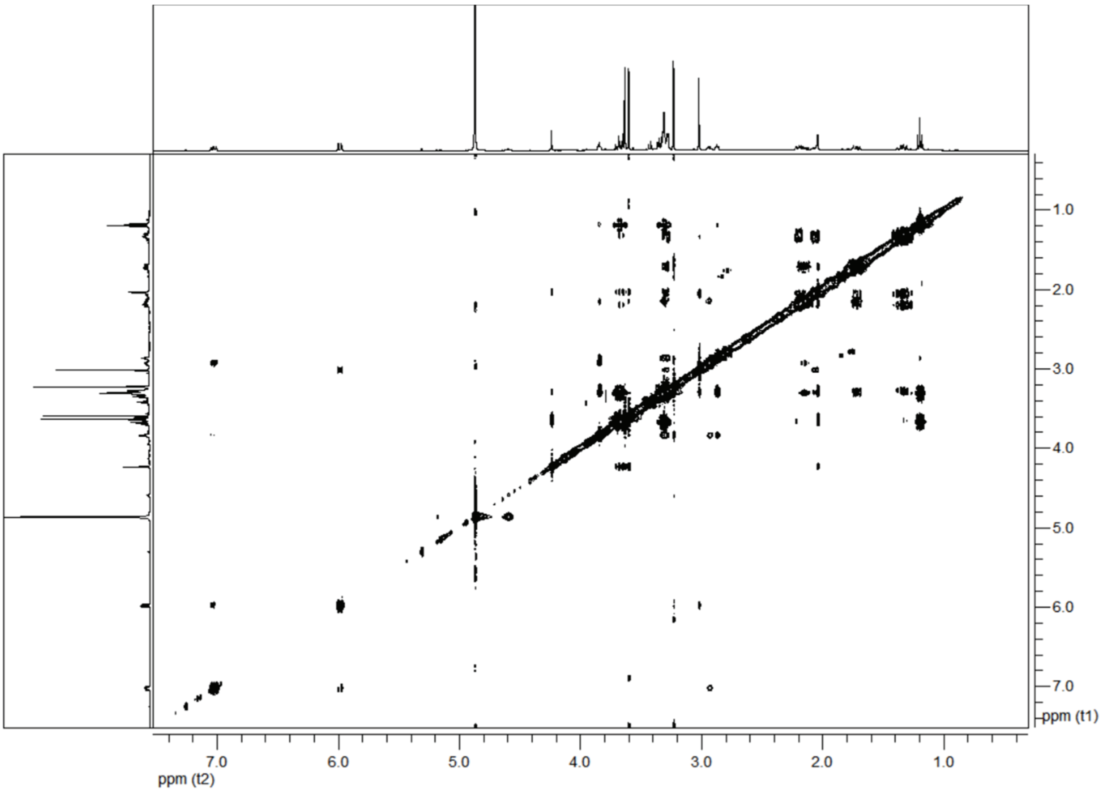

Figure S11. NOESY spectrum of brunodelphinine B (**1**).

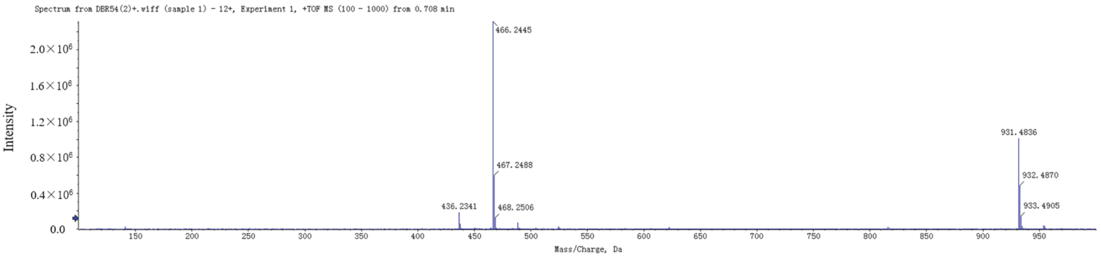

Figure S12. HR-ESI-MS spectrum of brunodelphinine C (**2**).

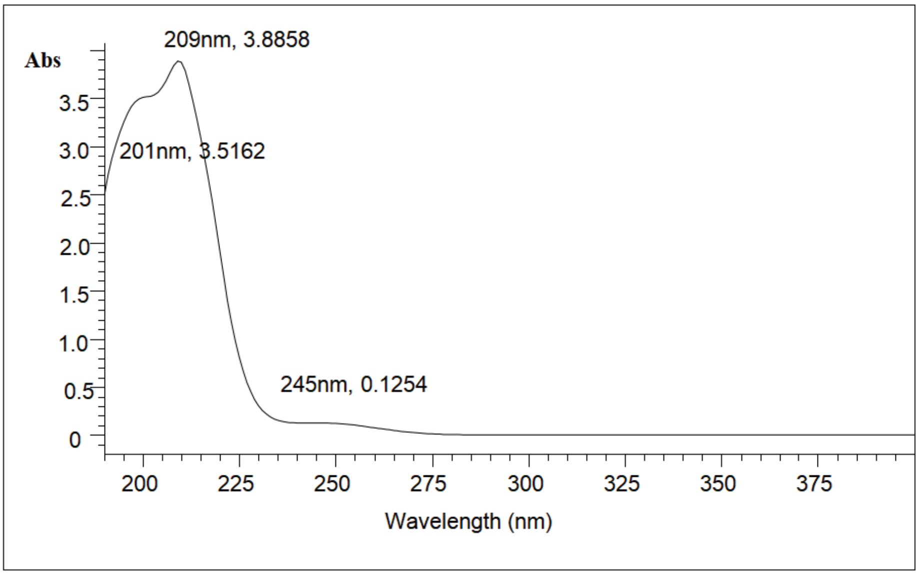

Figure S13. UV spectrum of brunodelphinine C (**2**).

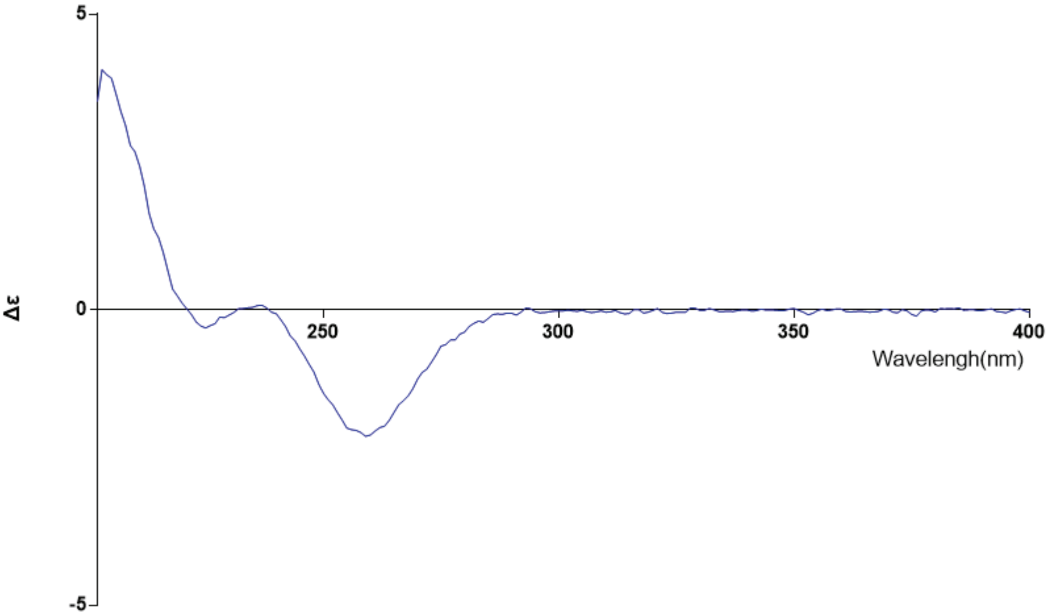

Figure S14. CD spectrum of brunodelphinine C (**2**).

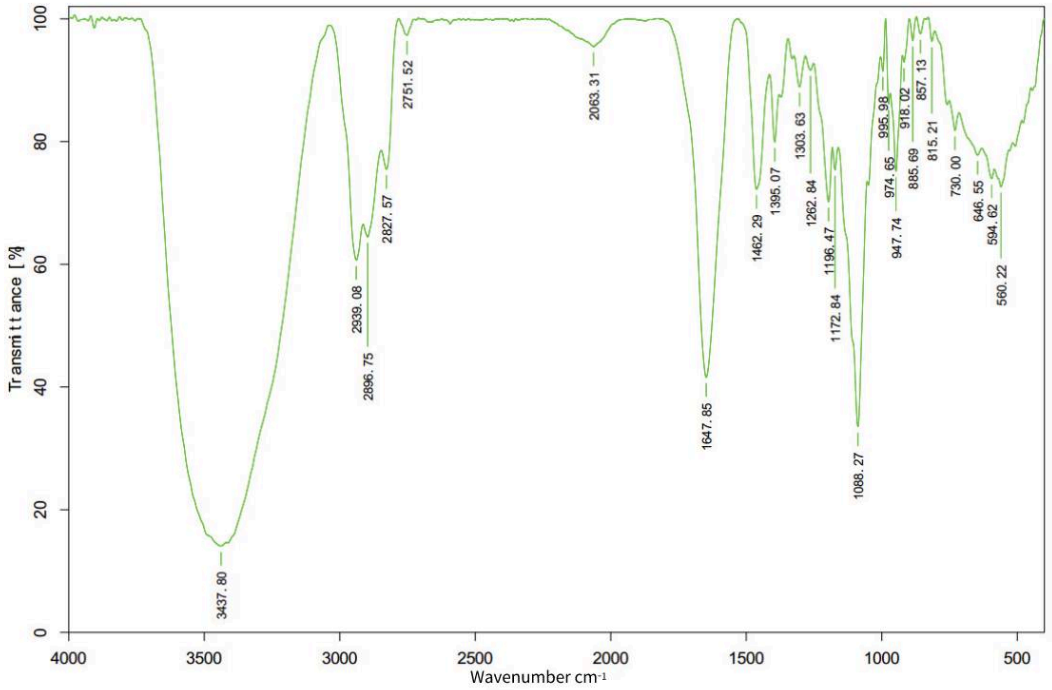

Figure S15. IR spectrum of brunodelphinine C (**2**).

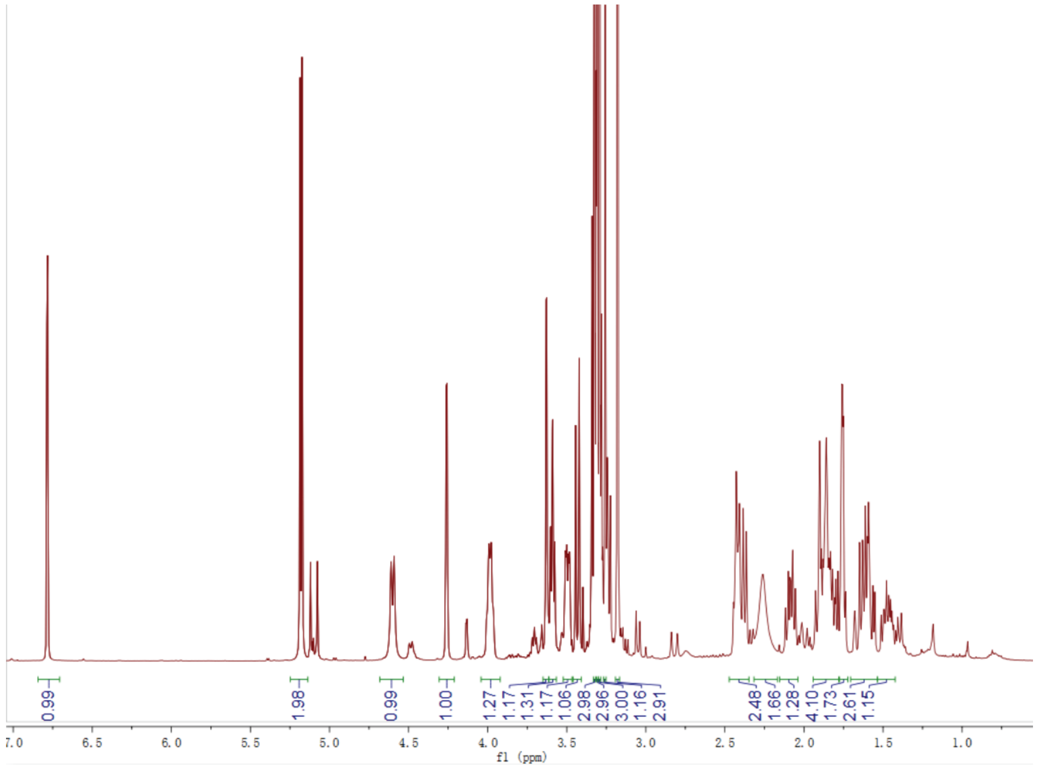

Figure S16. ^{1}H NMR spectrum of brunodelphinine C (**2**).

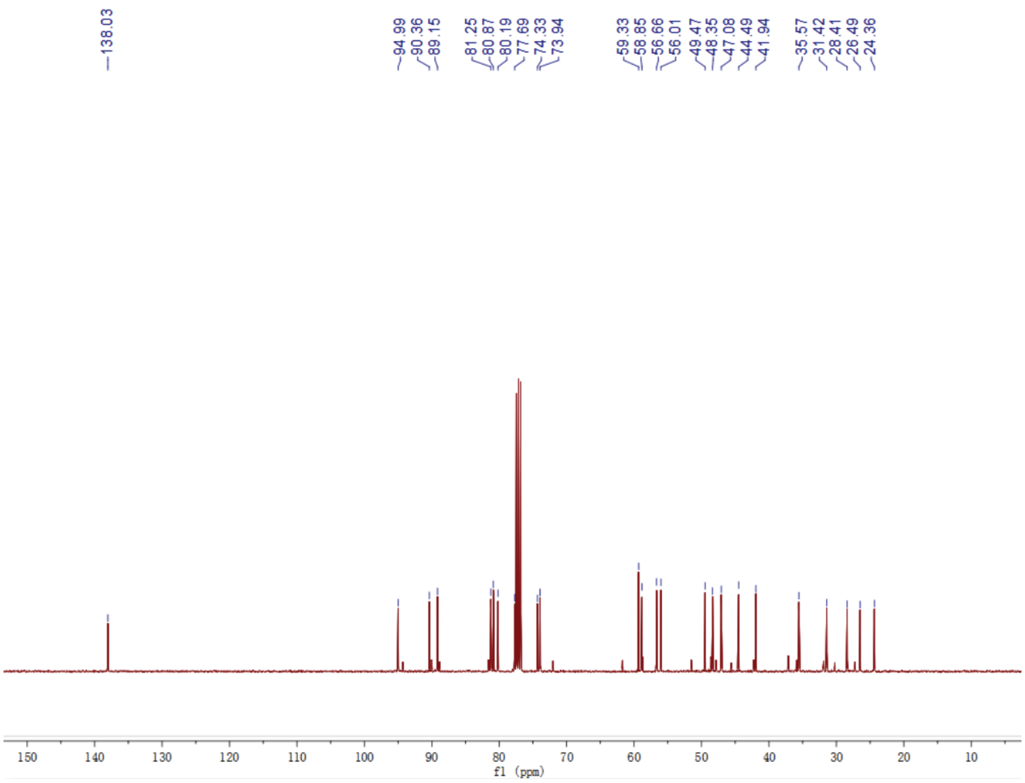

Figure S17. ^{1}C NMR spectrum of brunodelphinine C (**2**).

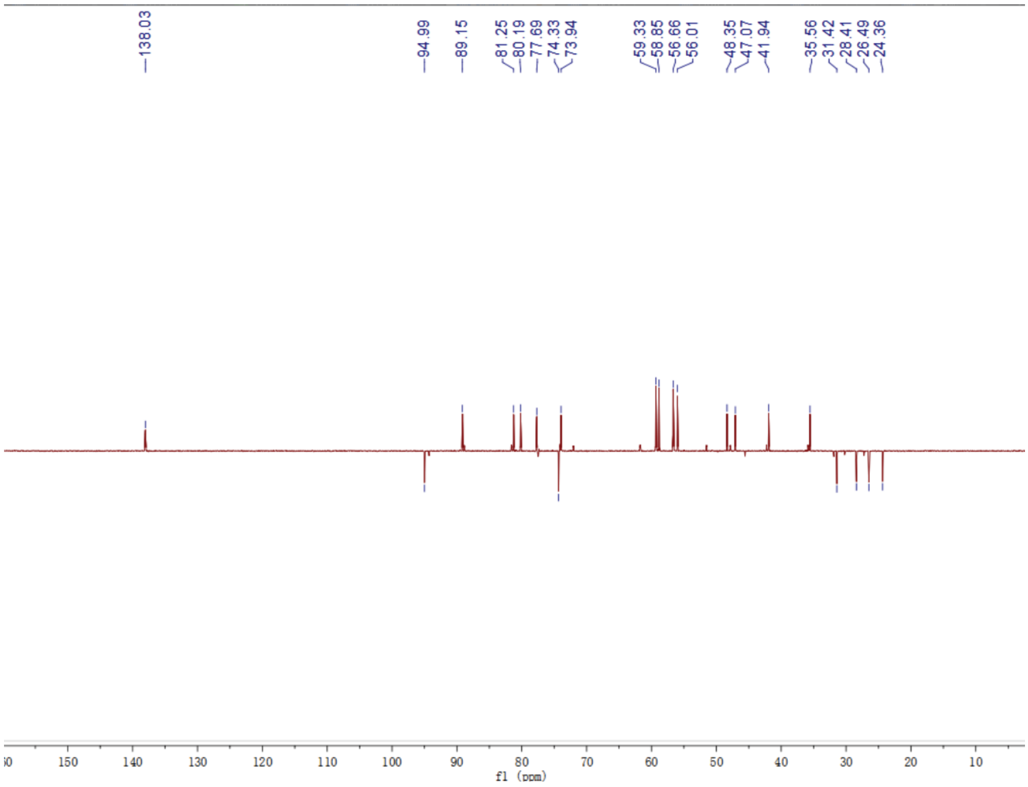

Figure S18. DEPT-135 spectrum of brunodelphinine C (**2**).

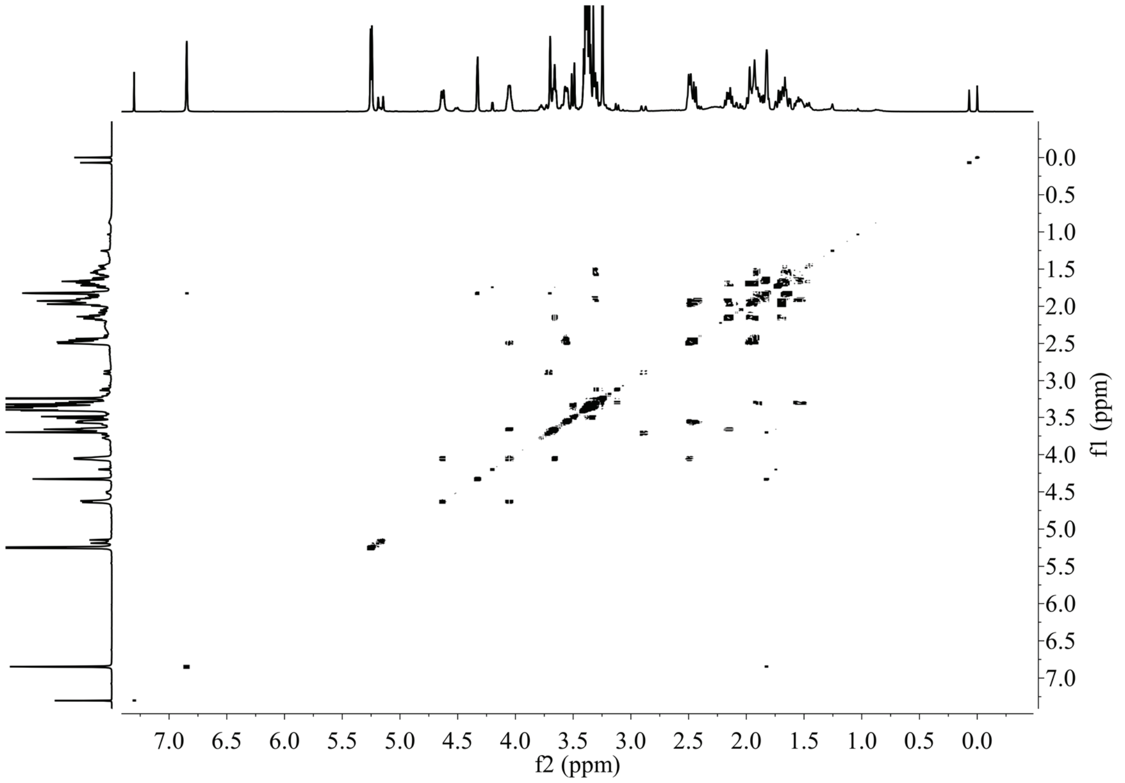

Figure S19. ^{1}H,^{1}H-COSY spectrum of brunodelphinine C (**2**).

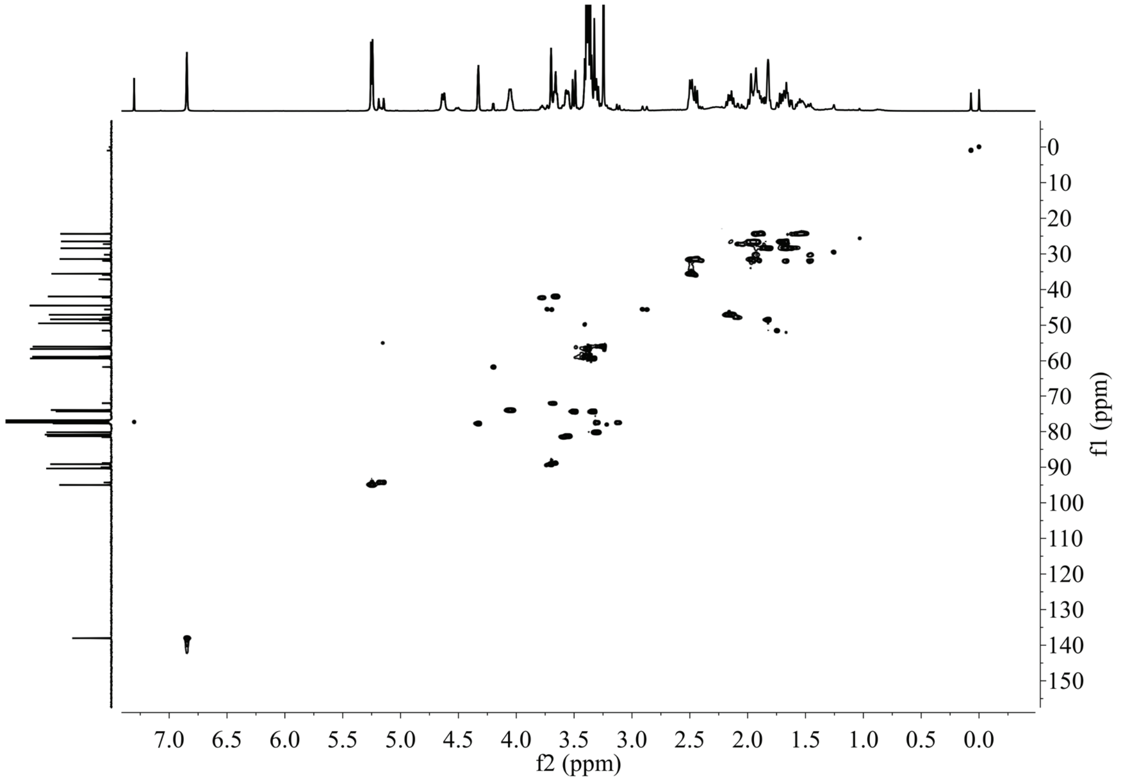

Figure S20. HSQC spectrum of brunodelphinine C (**2**).

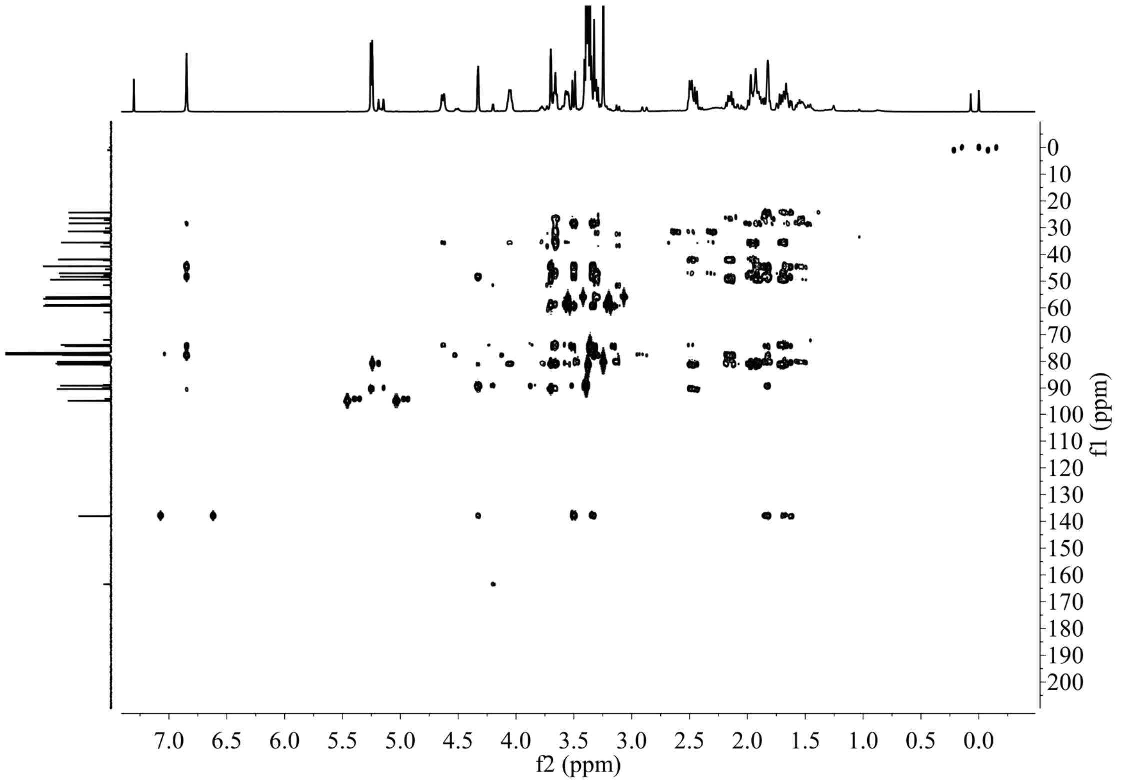

Figure S21. HMBC spectrum of brunodelphinine C (**2**).

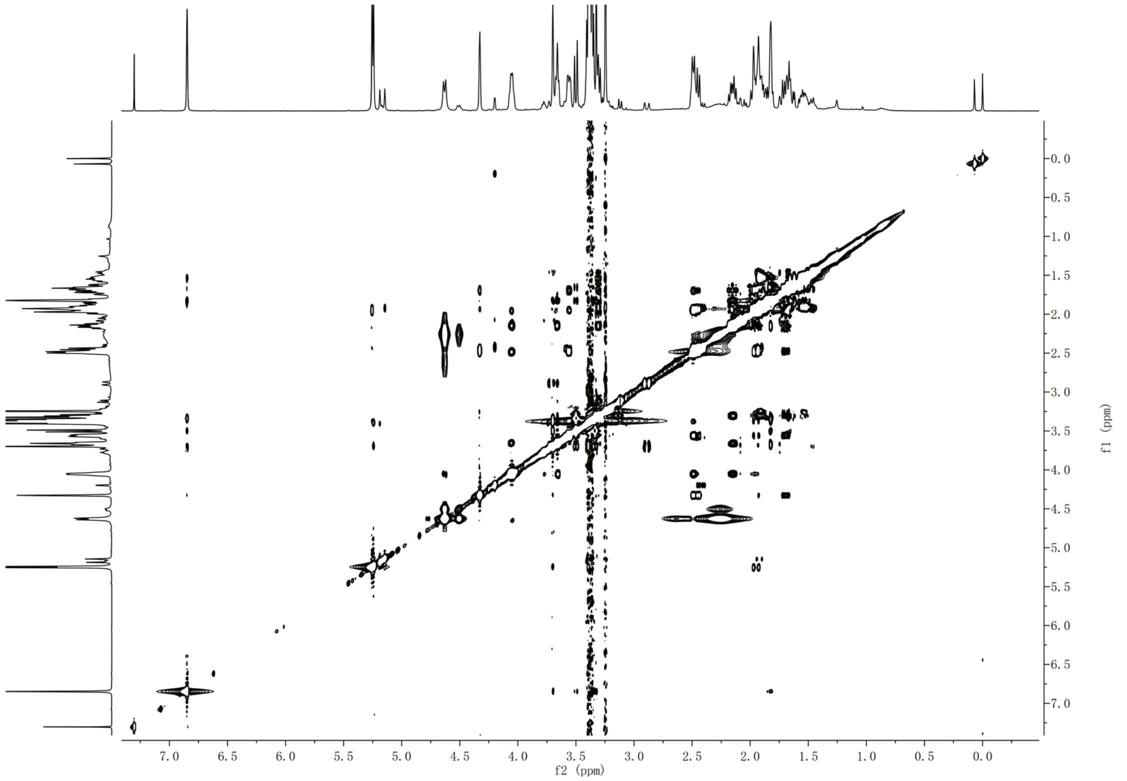

Figure S22. NOESY spectrum of brunodelphinine C (**2**).

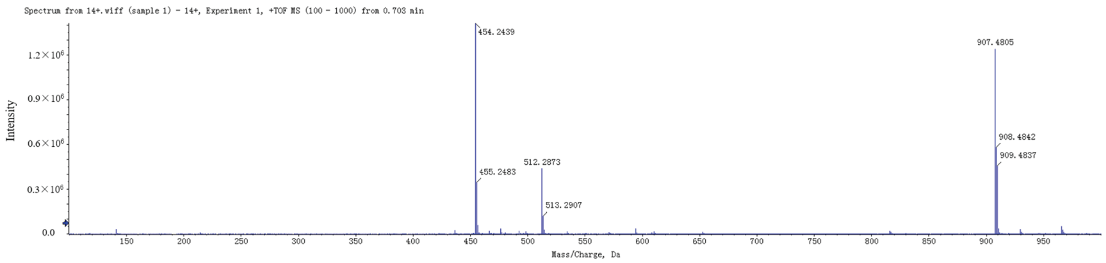

Figure S23. HR-ESI-MS spectrum of brunodelphinine D (**3**).

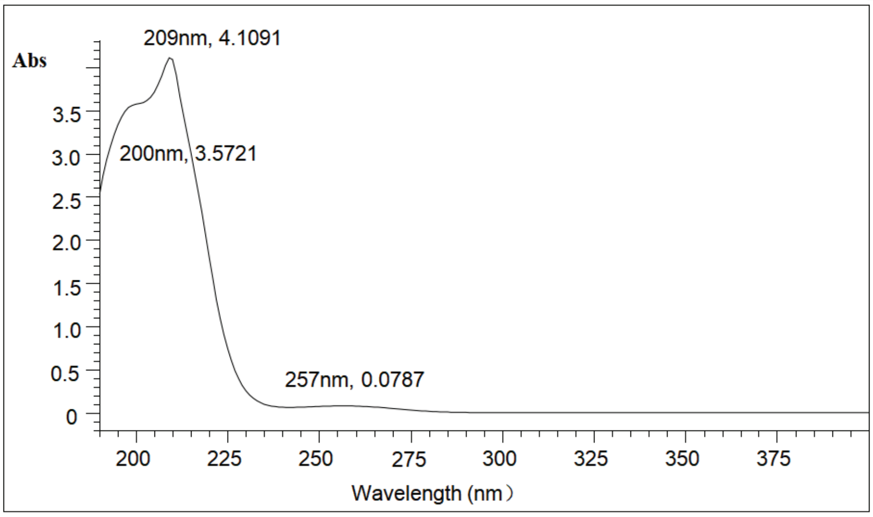

Figure S24. UV spectrum of brunodelphinine D (**3**).

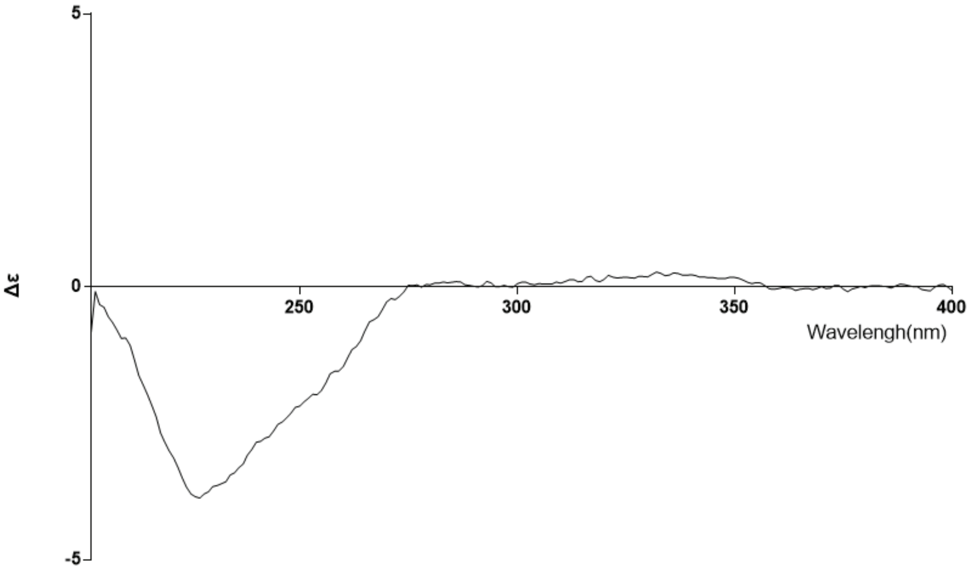

Figure S25. CD spectrum of brunodelphinine D (**3**).

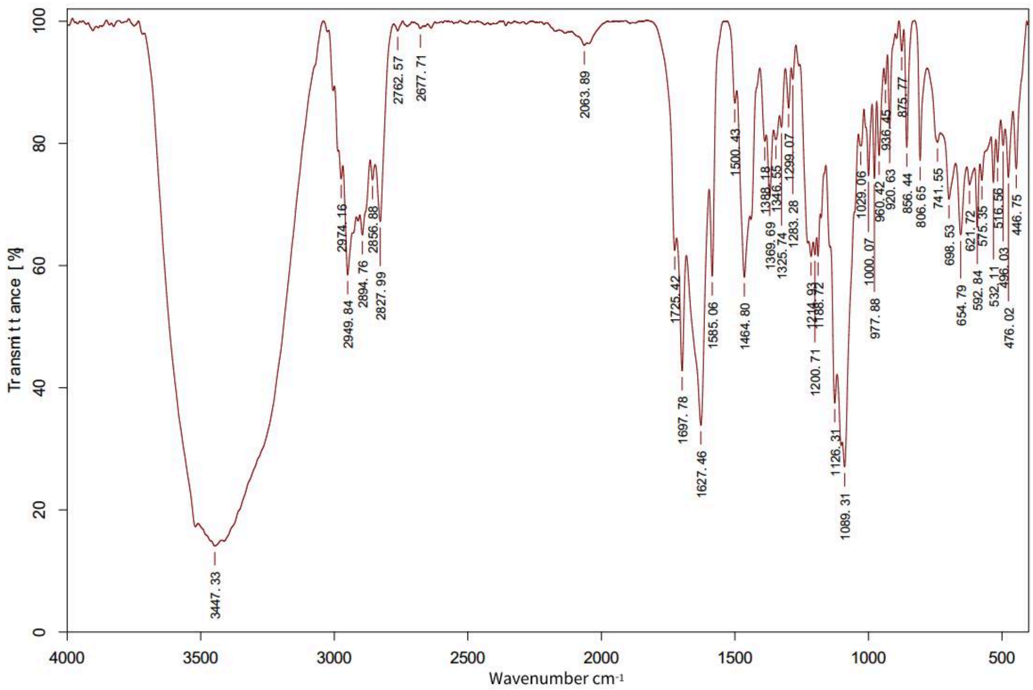

Figure S26. IR spectrum of brunodelphinine D (**3**).

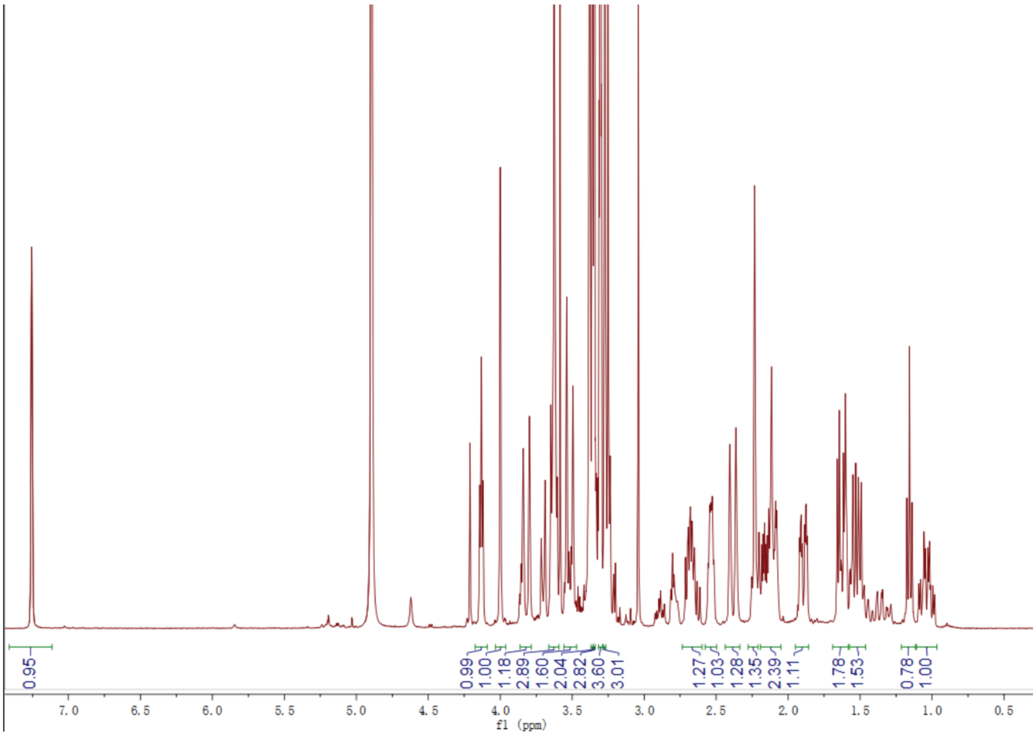

Figure S27. ^{1}H NMR spectrum of brunodelphinine D (**3**).

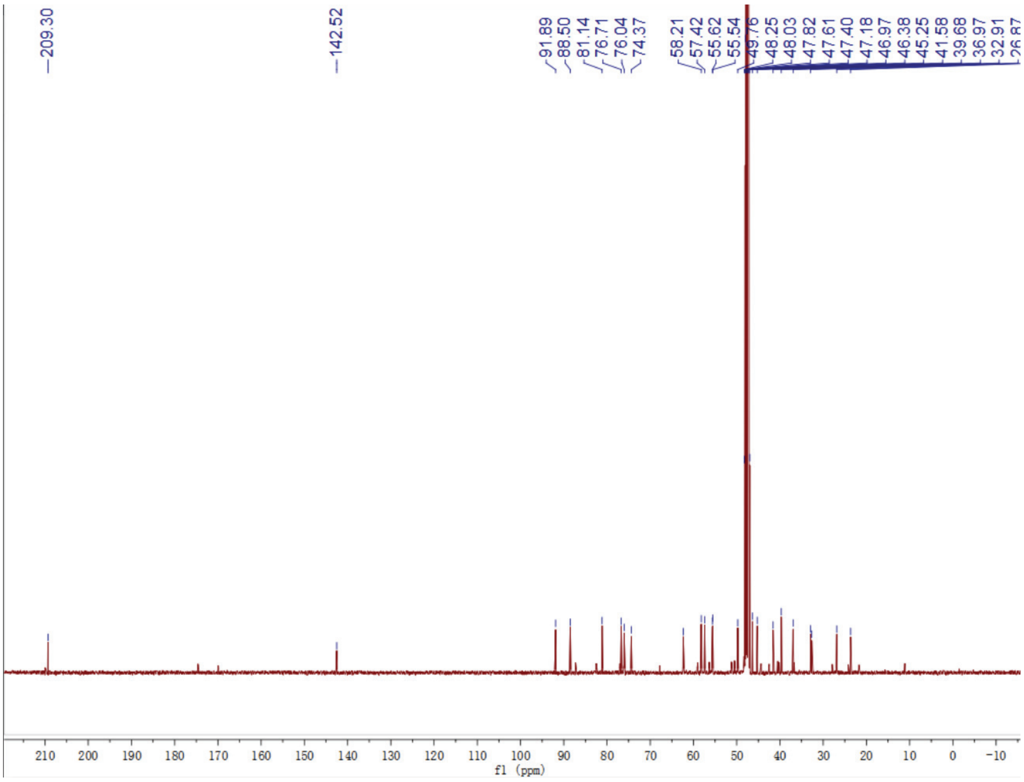

Figure S28. ^{1}C NMR spectrum of brunodelphinine D (**3**).

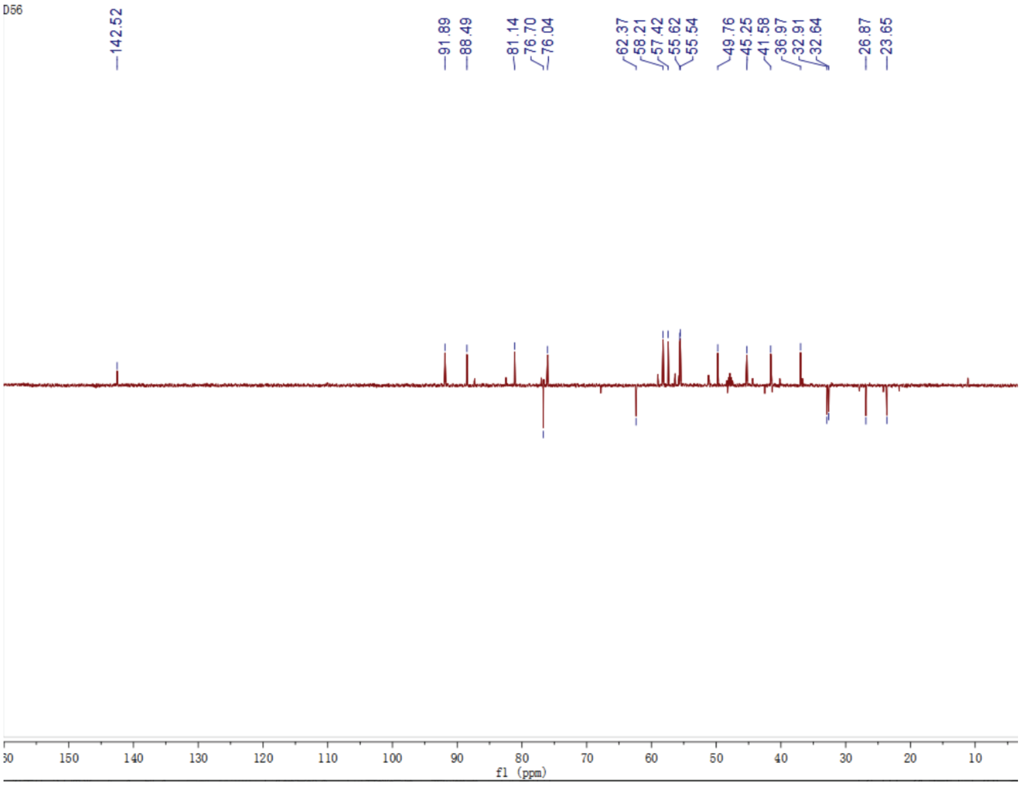

Figure S29. DEPT-135 spectrum of brunodelphinine D (**3**).

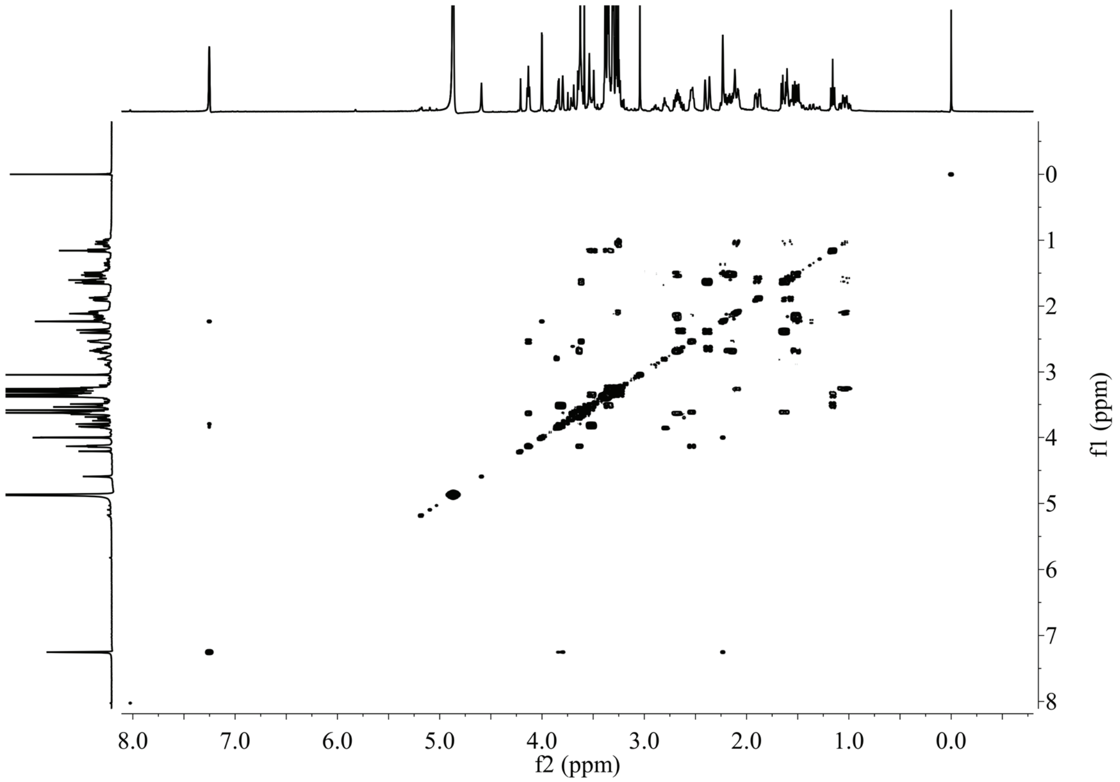

Figure S30. $^1H,^1H$-COSY spectrum of brunodelphinine D (**3**).

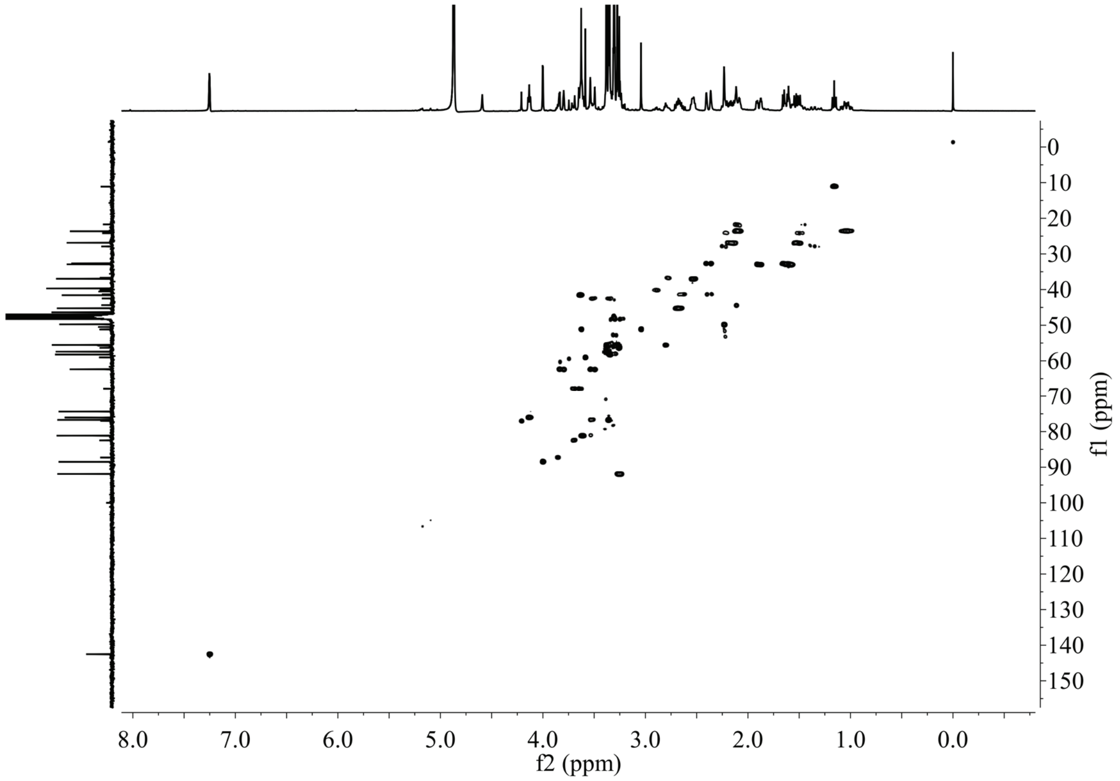

Figure S31. HSQC spectrum of brunodelphinine D (**3**).

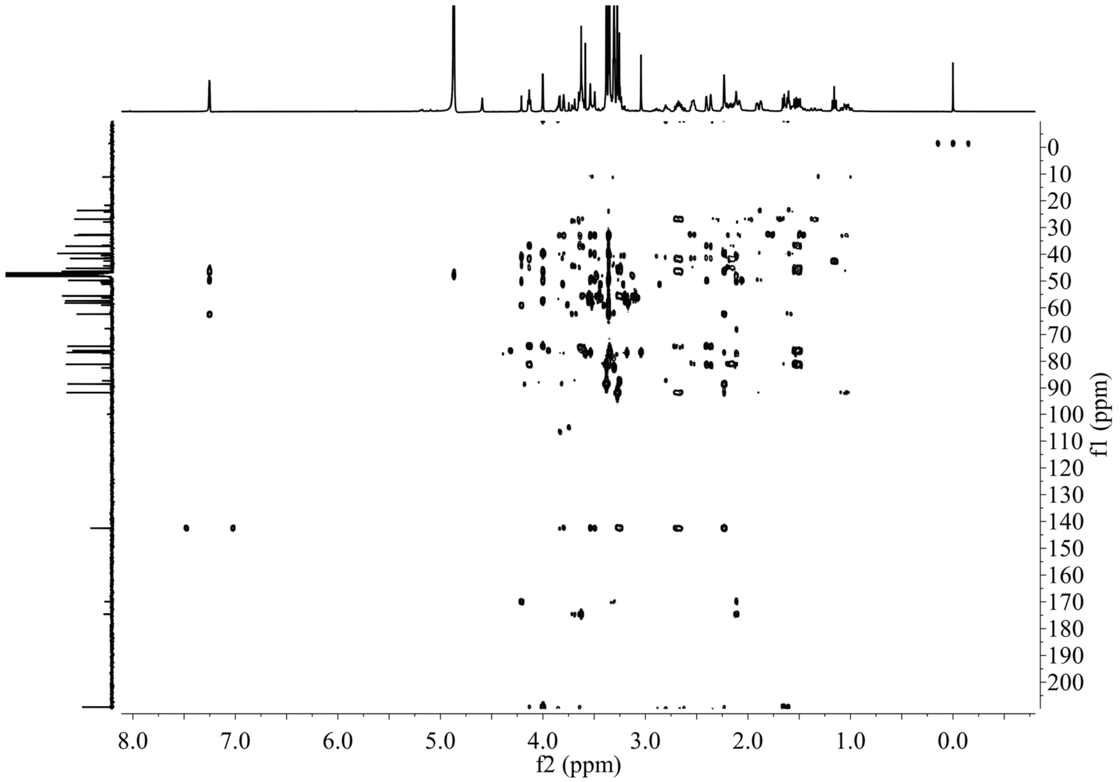

Figure S32. HMBC spectrum of brunodelphinine D (**3**).

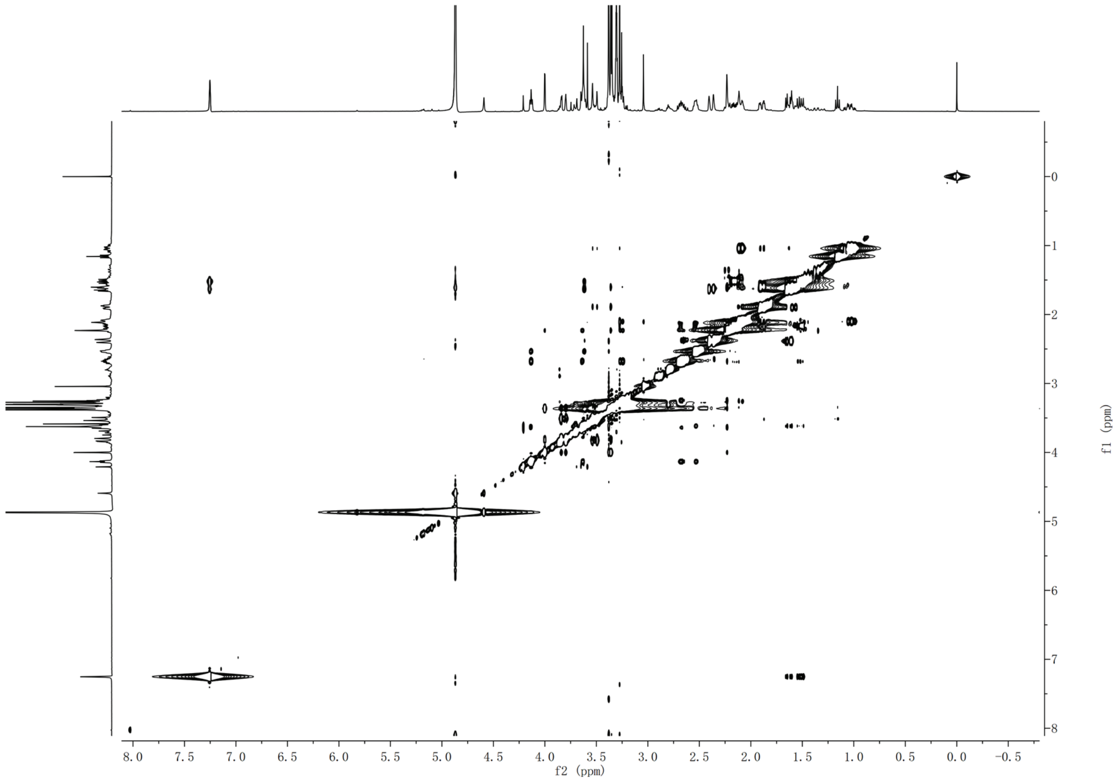

Figure S33. NOESY spectrum of brunodelphinine D (**3**).

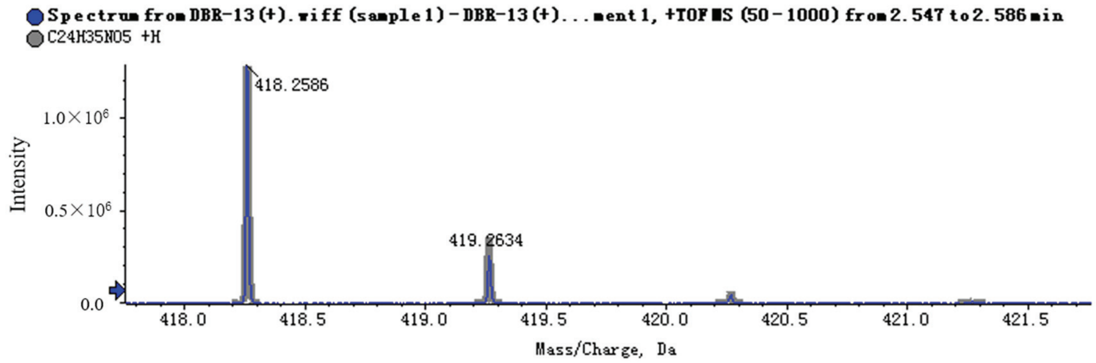

Figure S34. HR-ESI-MS spectrum of brunodelphinine E (**4**).

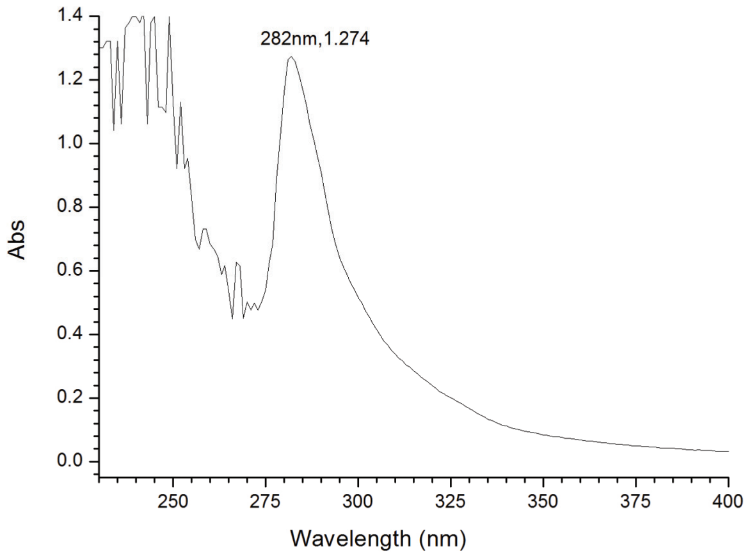

Figure S35. UV spectrum of brunodelphinine E (**4**).

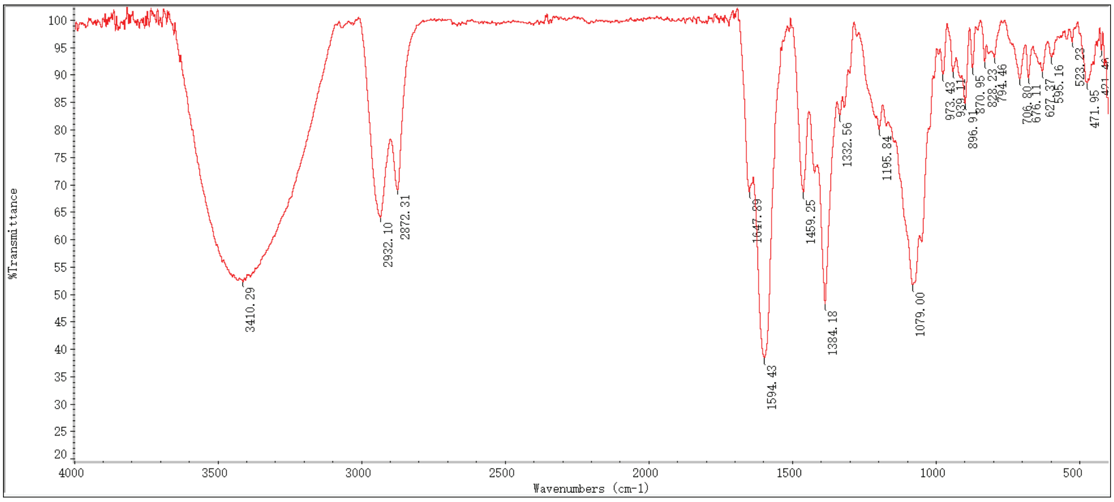

Figure S36. IR spectrum of brunodelphinine E (**4**).

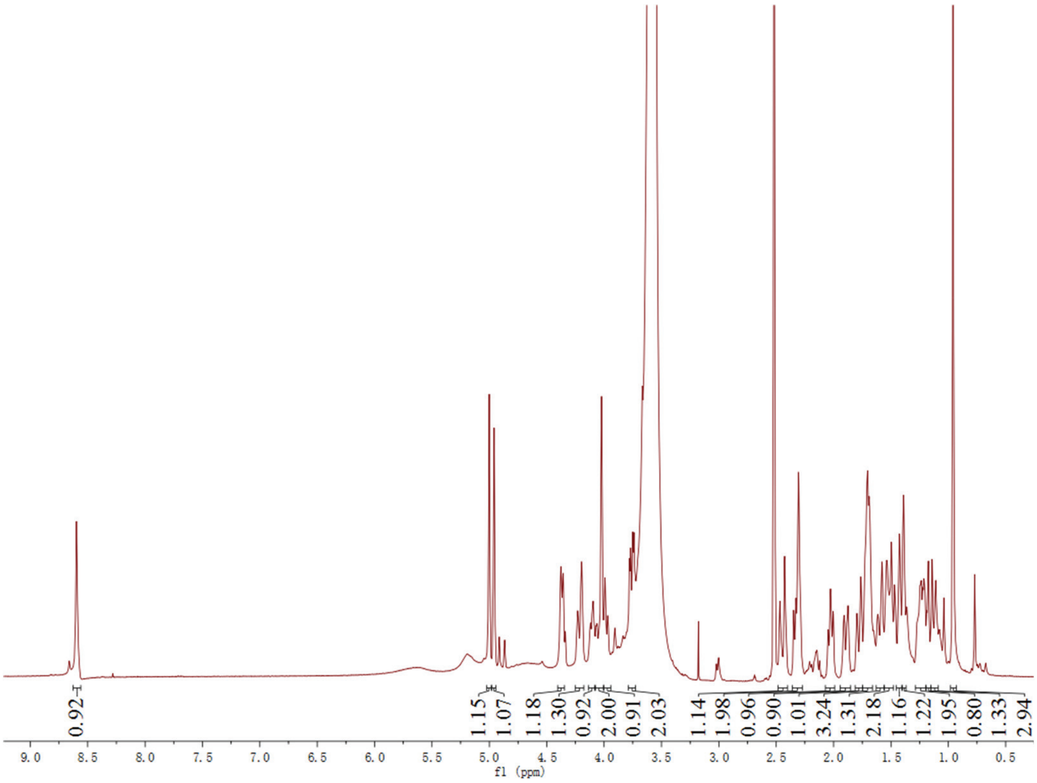

Figure S37. ^{1}H NMR spectrum of brunodelphinine E (**4**).

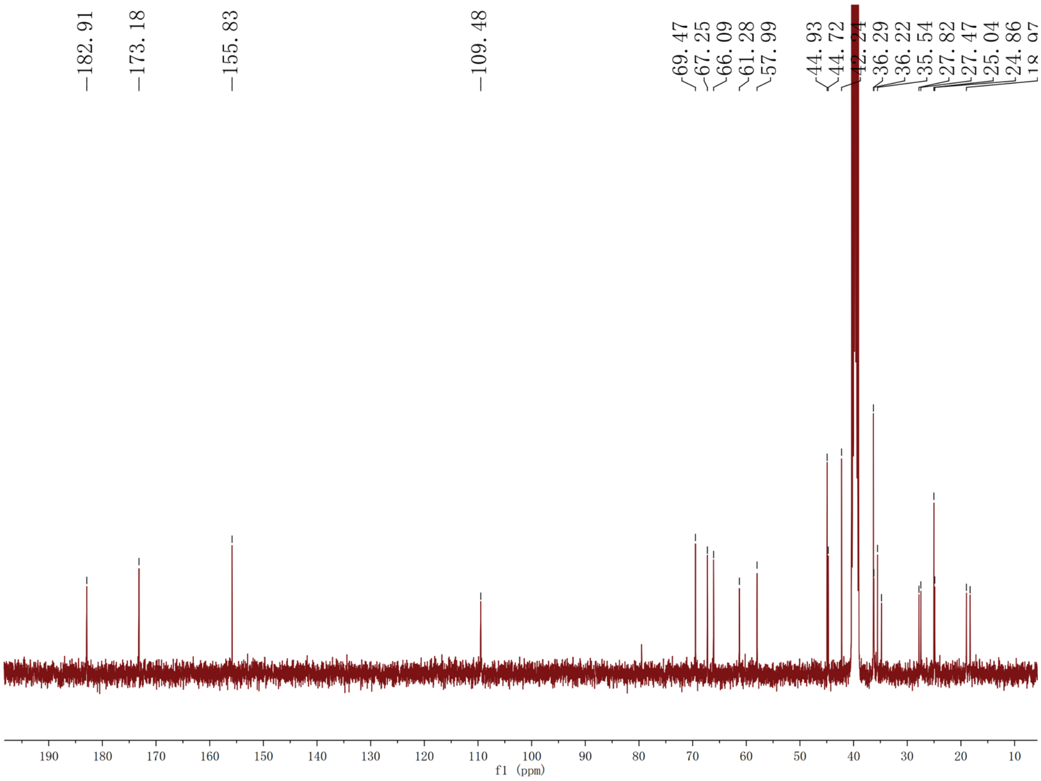

Figure S38. ^{1}C NMR spectrum of brunodelphinine E (**4**).

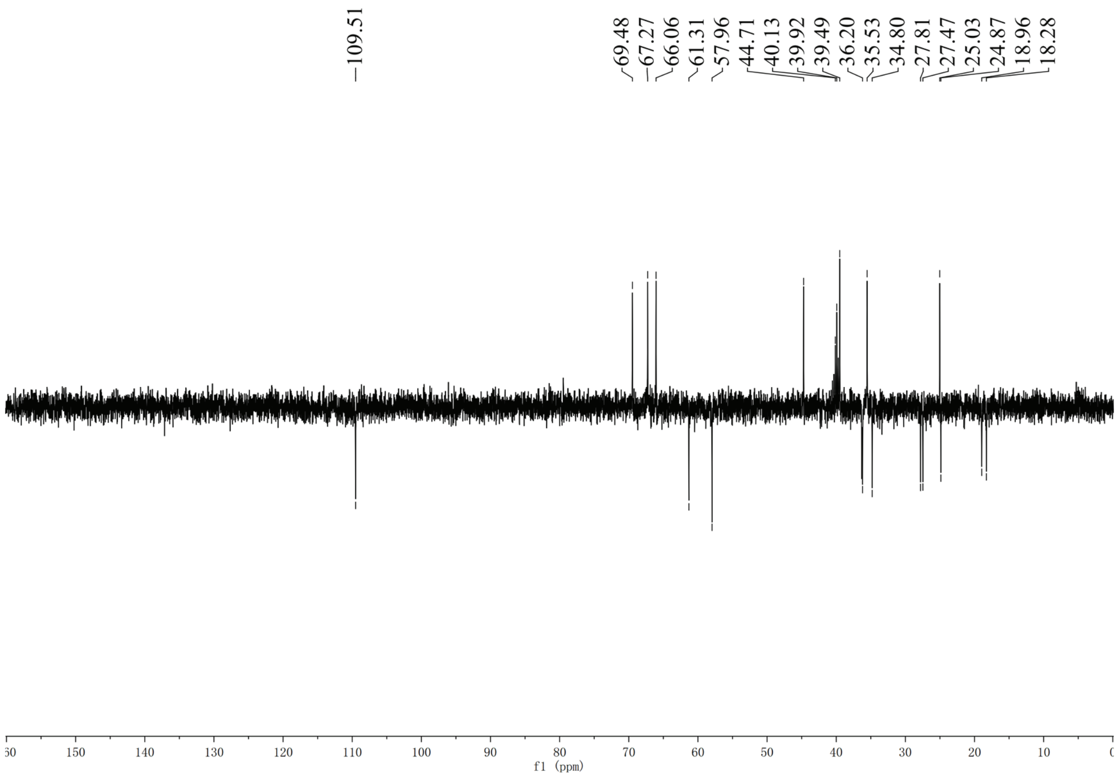

Figure S39. DEPT-135 spectrum of brunodelphinine E (**4**).

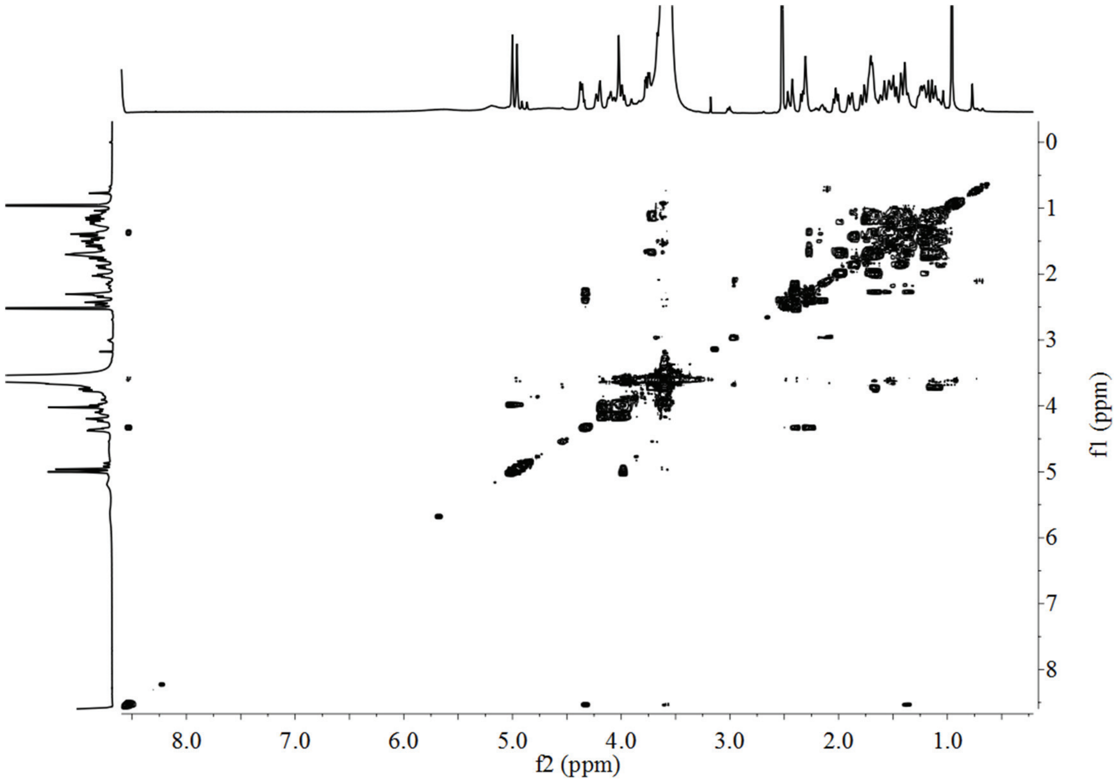

Figure S40. ^{1}H,^{1}H-COSY spectrum of brunodelphinine E (**4**).

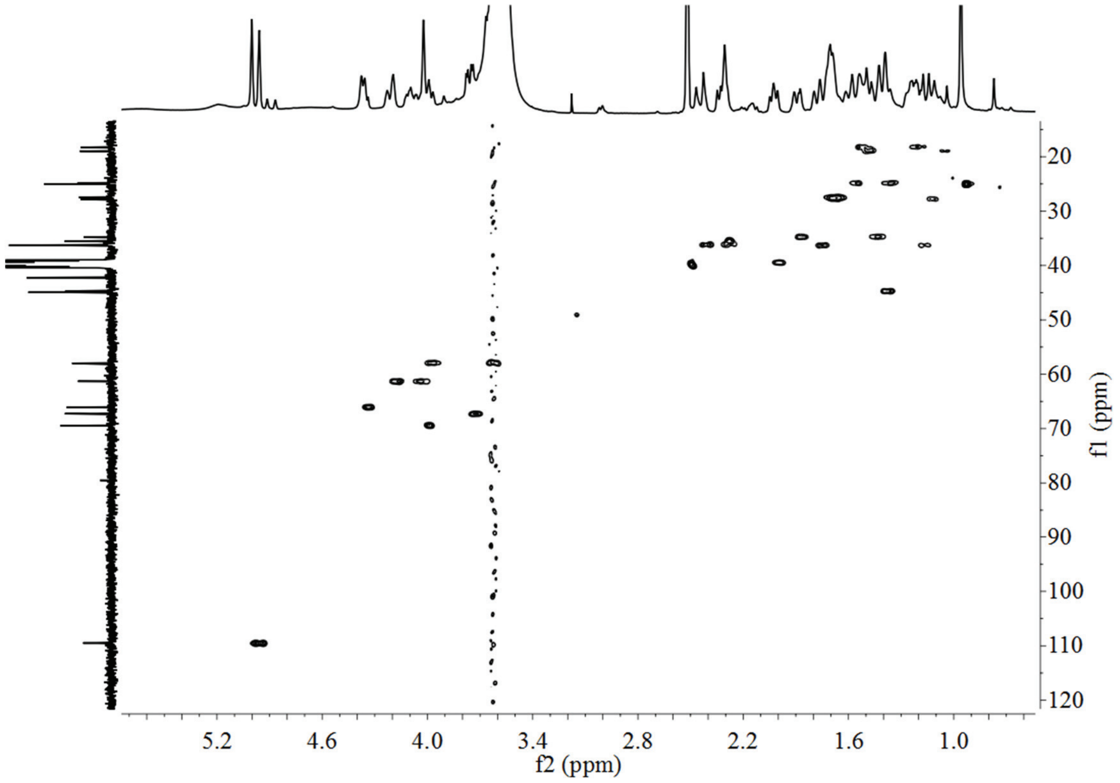

Figure S41. HSQC spectrum of brunodelphinine E (**4**).

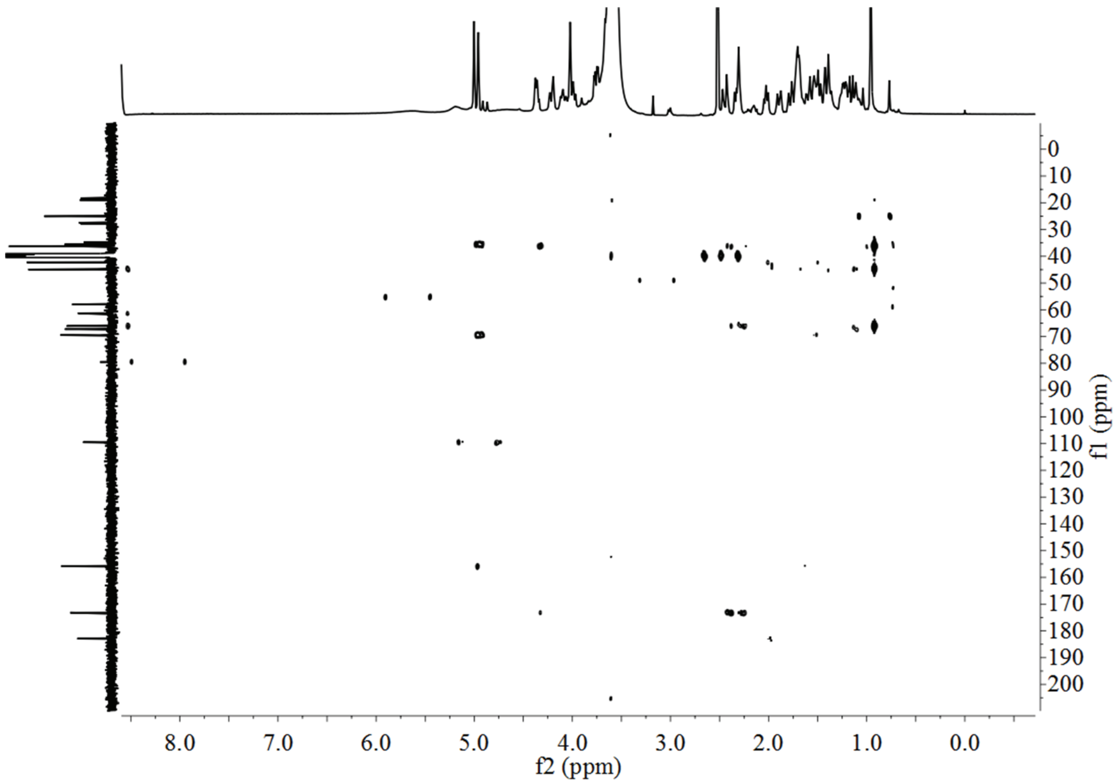

Figure S42. HMBC spectrum of brunodelphinine D (**4**).

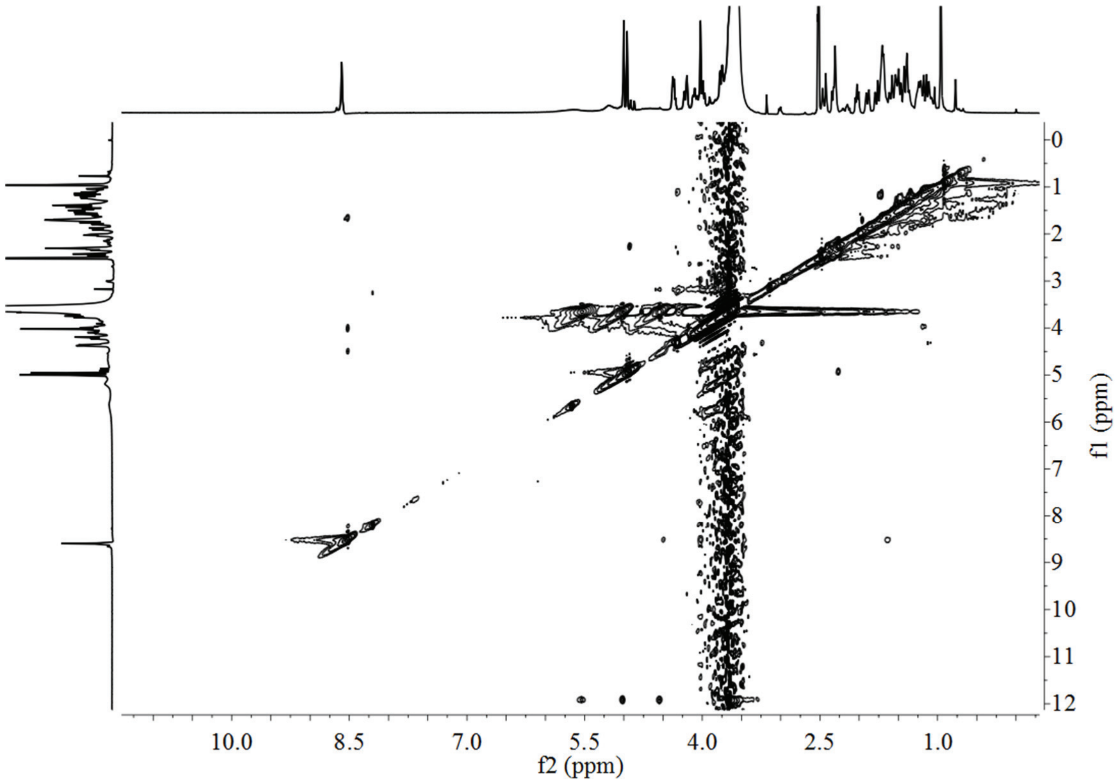

Figure S43. NOESY spectrum of brunodelphinine E (**4**).

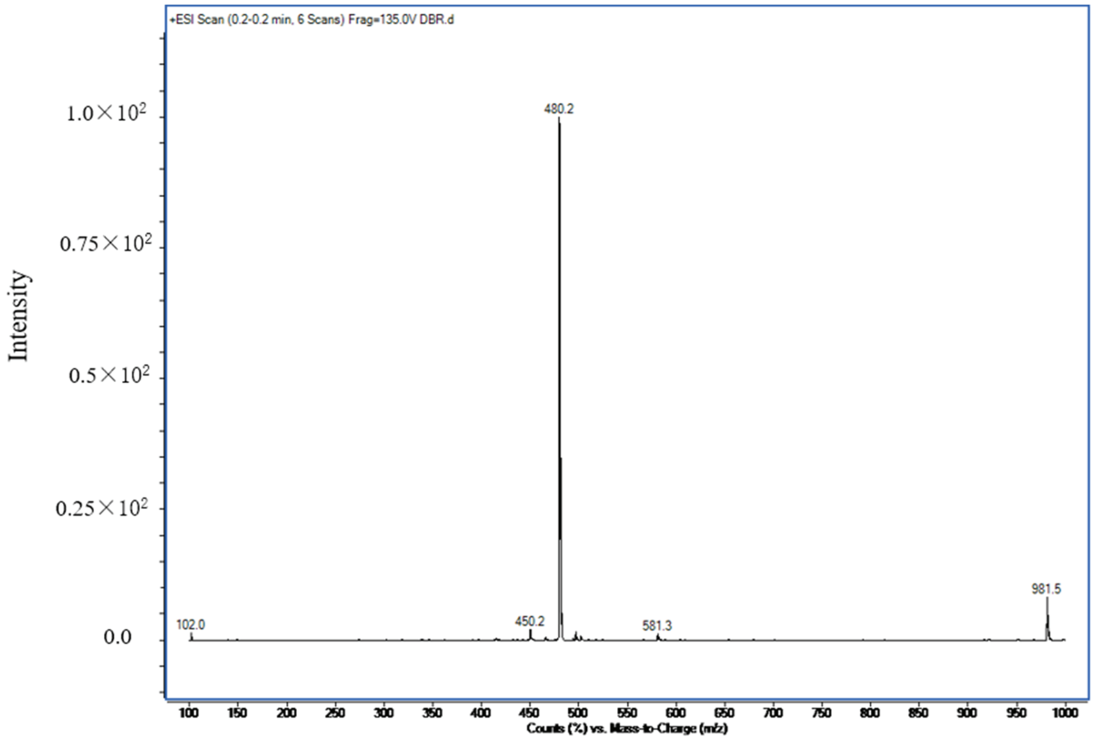

Figure S44. HR-ESI-MS spectrum of delbruline (**5**).

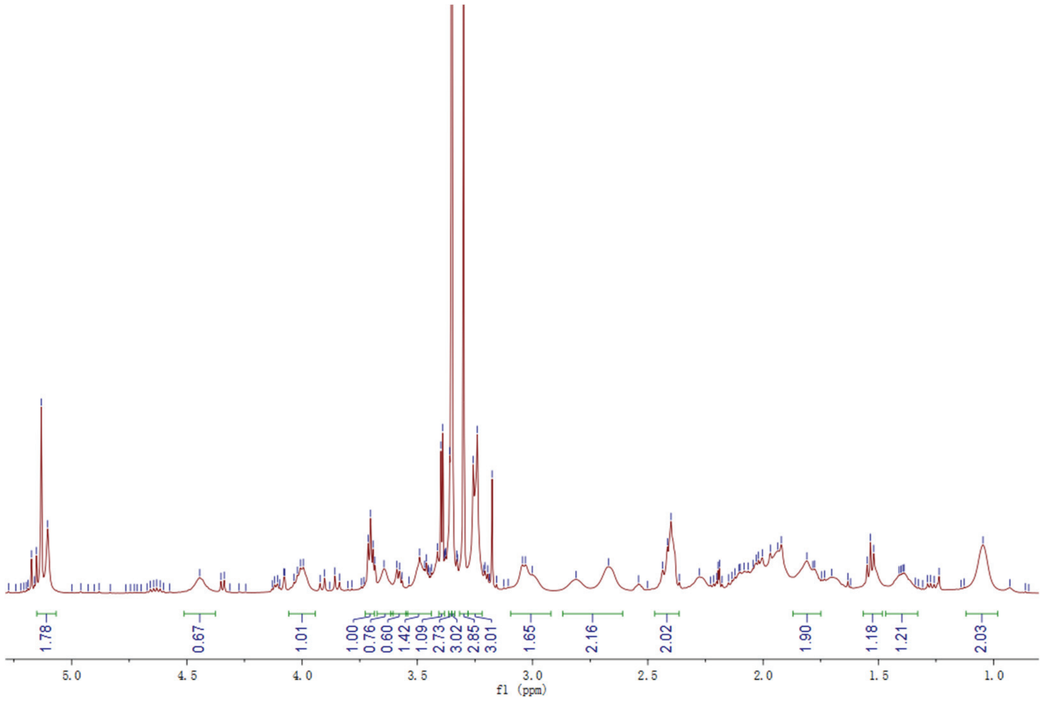

Figure S45. ^{1}H NMR spectrum of delbruline (**5**).

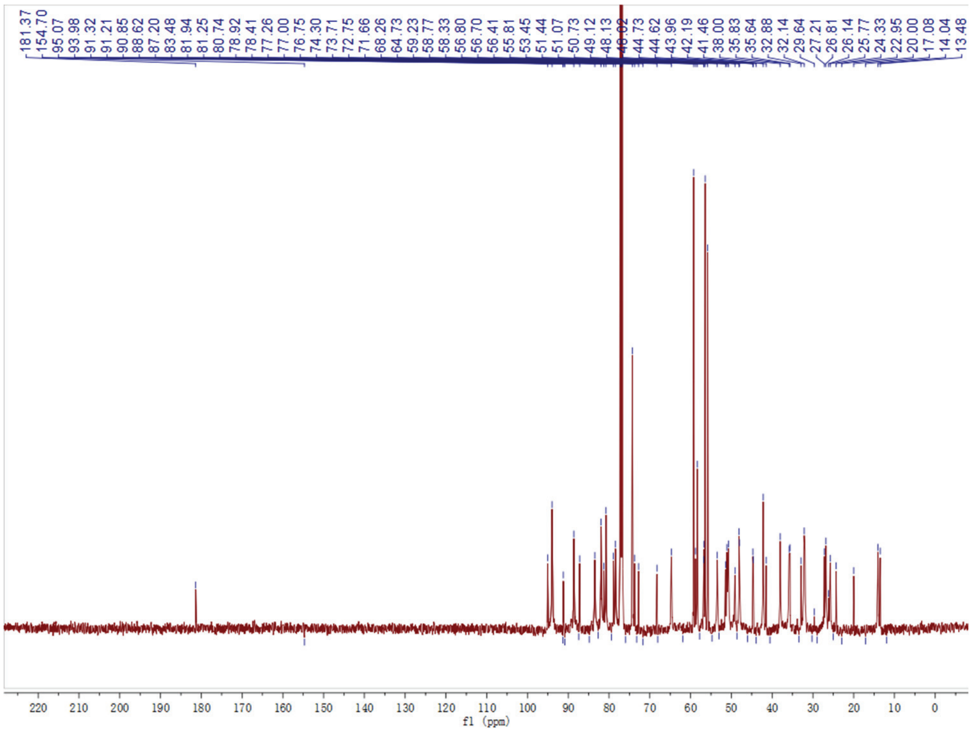

Figure S46. ^{1}C NMR spectrum of delbruline (**5**).

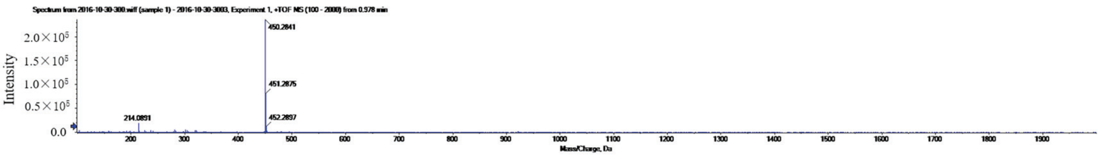

Figure S47. HR-ESI-MS spectrum of delpheline (**6**).

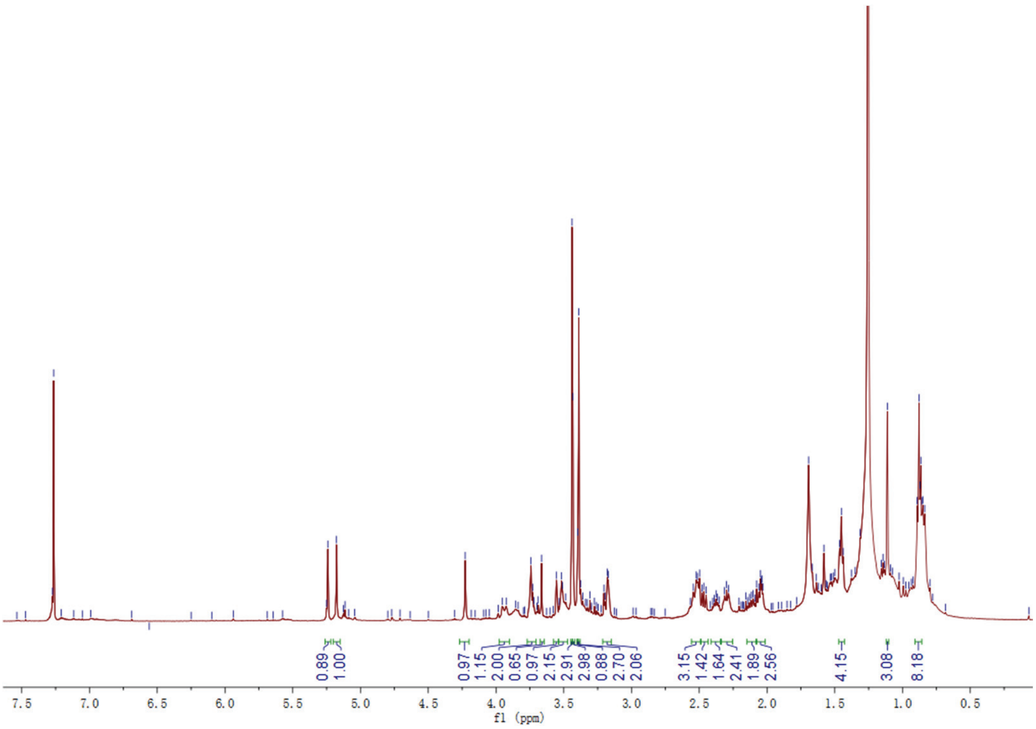

Figure S48. ^{1}H NMR spectrum of delpheline (**6**).

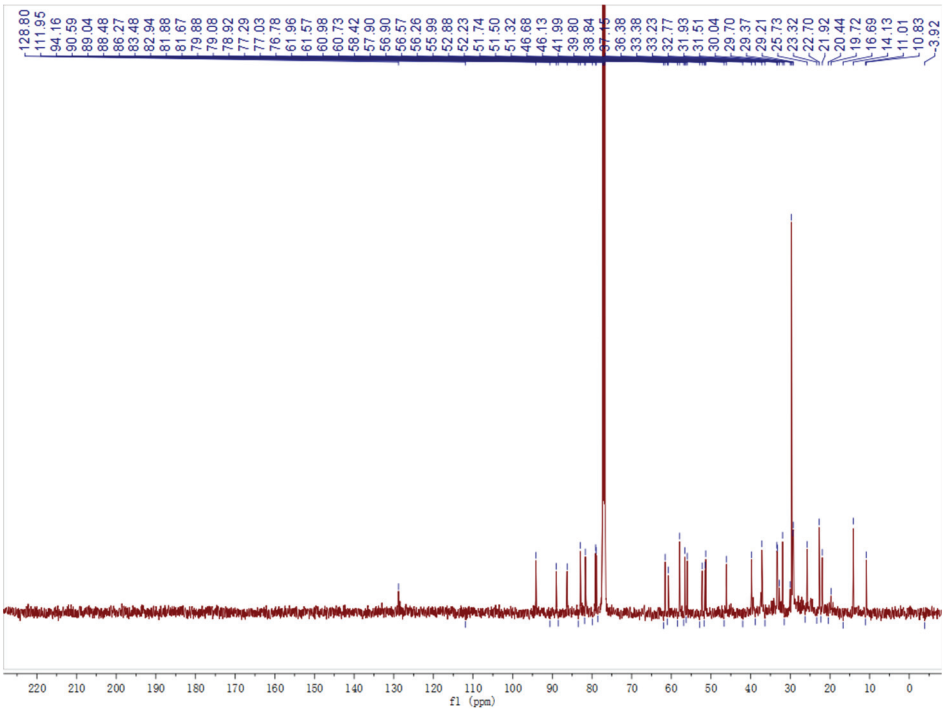

Figure S49. ^{1}C NMR spectrum of delpheline (**6**).

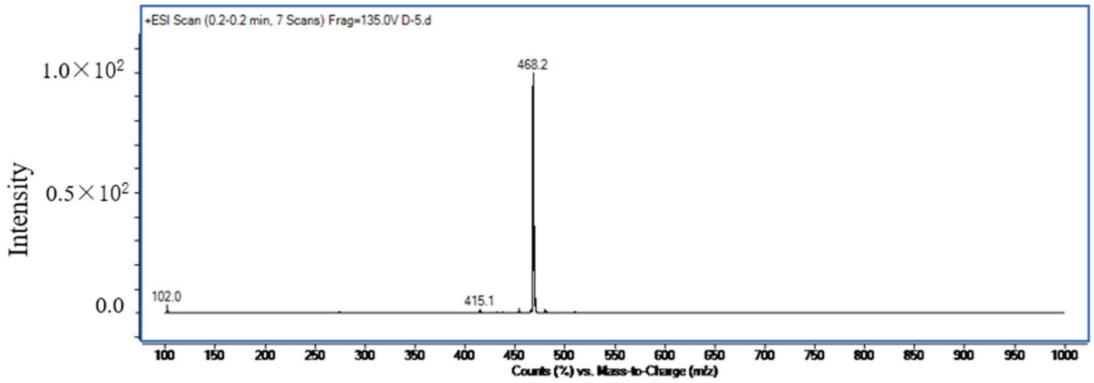

Figure S50. HR-ESI-MS spectrum of lycoctonine (**7**).

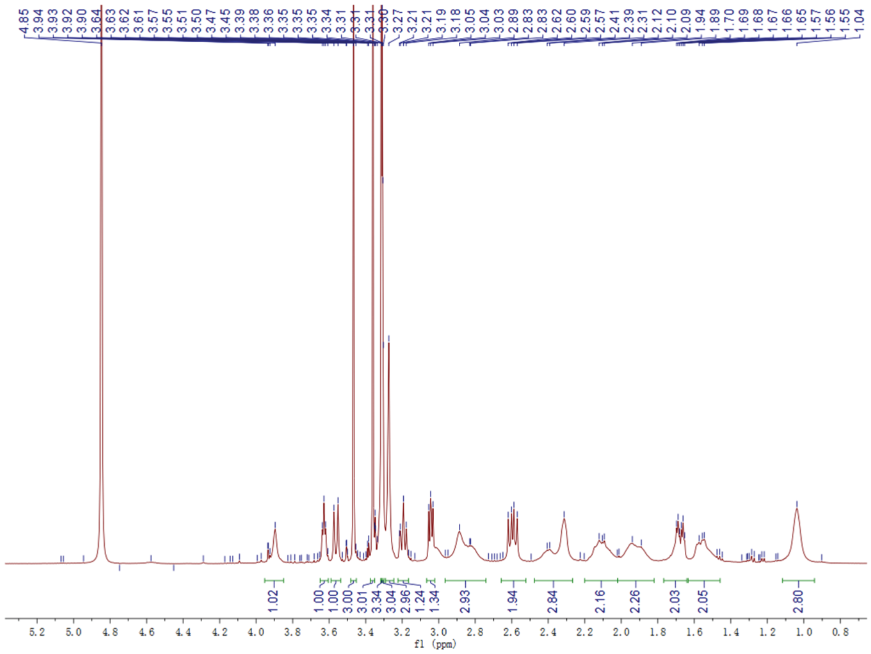

Figure S51. ¹H NMR spectrum of lycoctonine (**7**).

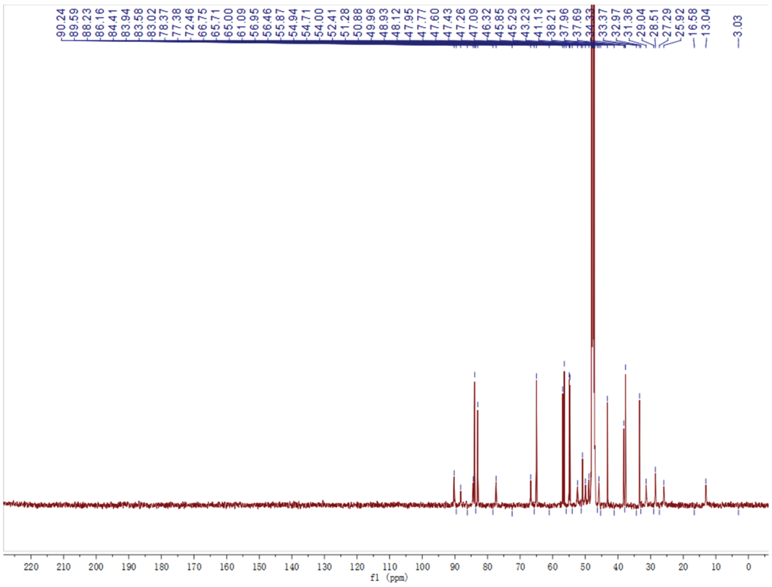

Figure S52. ¹C NMR spectrum of lycoctonine (**7**).

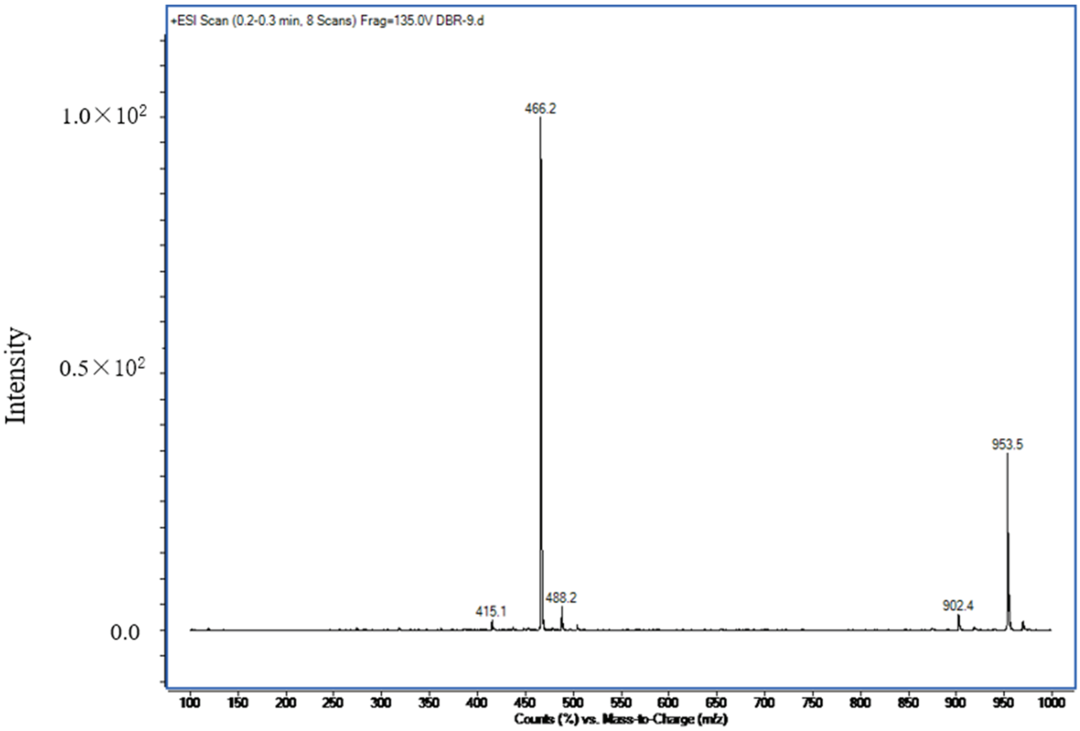

Figure S53. HR-ESI-MS spectrum of delbrunine (**8**).

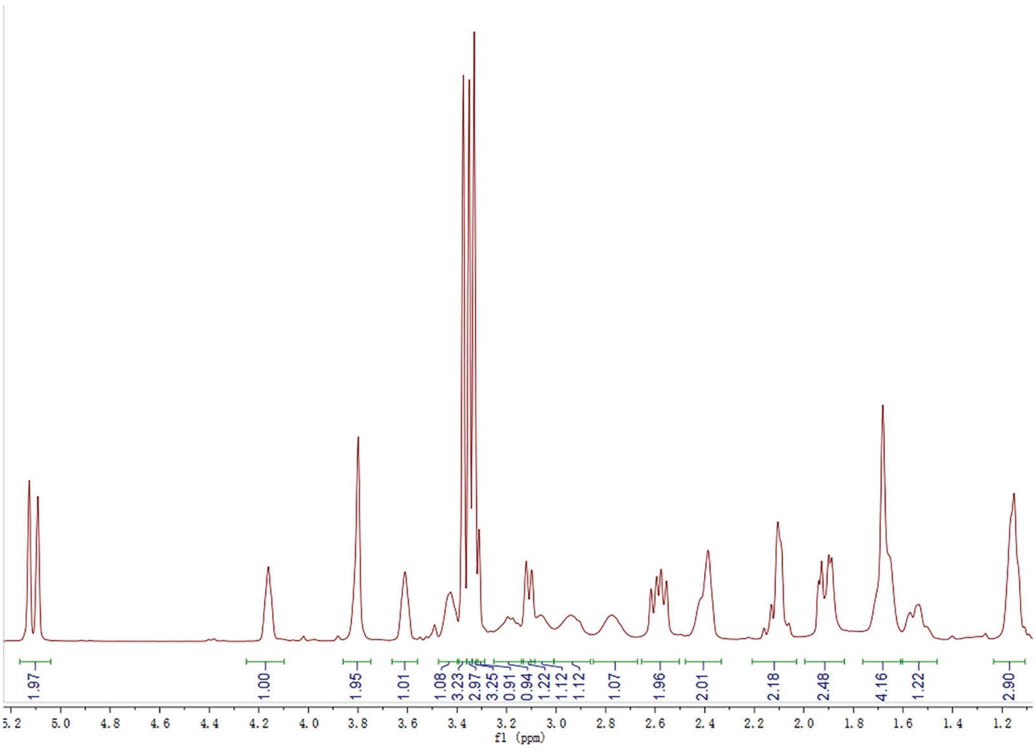

Figure S54. ^{1}H NMR spectrum of delbrunine (**8**).

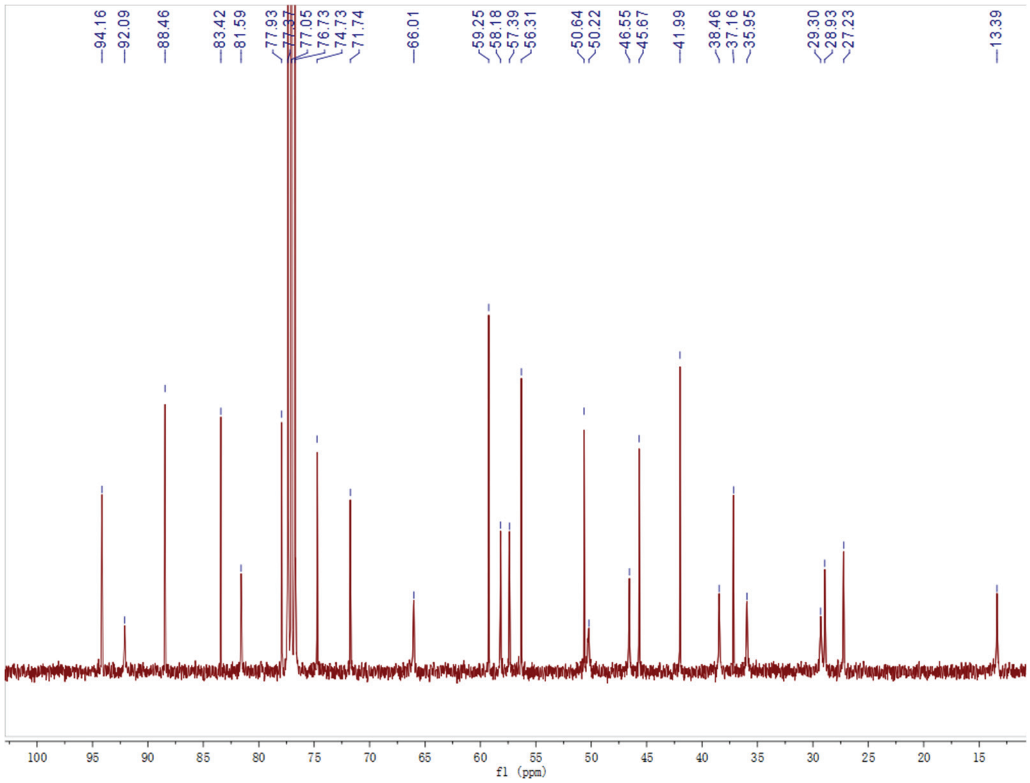

Figure S55. ^{1}C NMR spectrum of delbrunine (**8**).

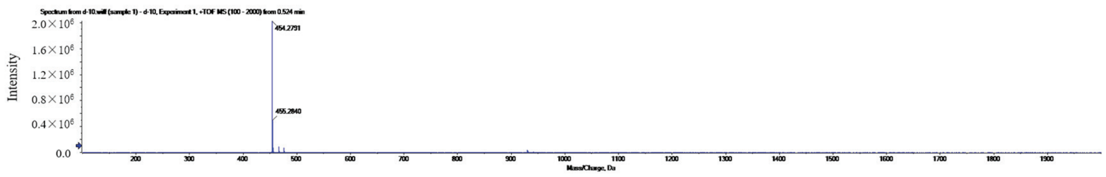

Figure S56. HR-ESI-MS spectrum of delcosine (**9**).

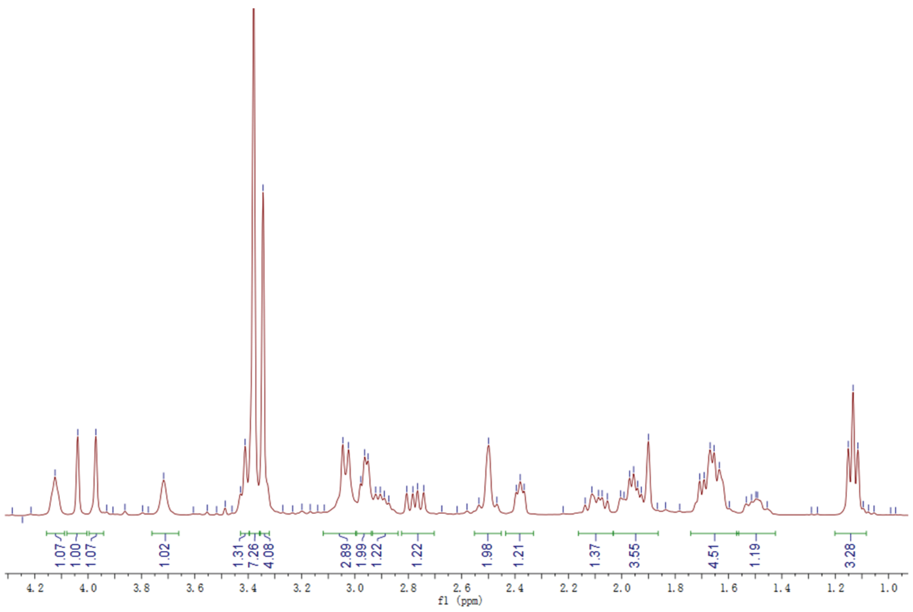

Figure S57. ^{1}H NMR spectrum of delcosine (**9**).

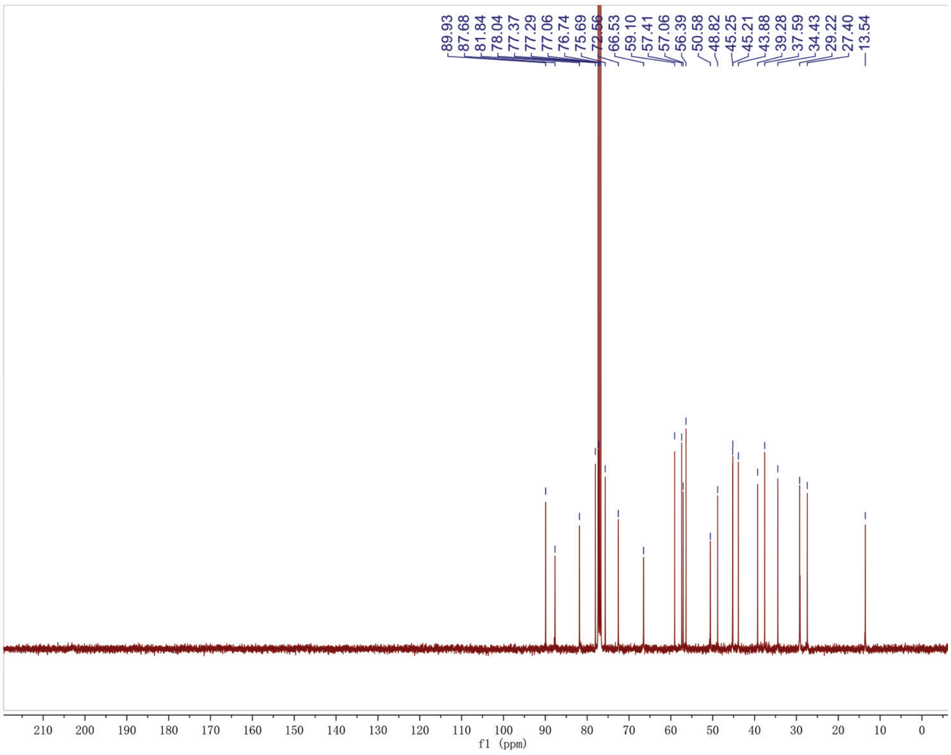

Figure S58. ^{1}C NMR spectrum of delcosine (**9**).

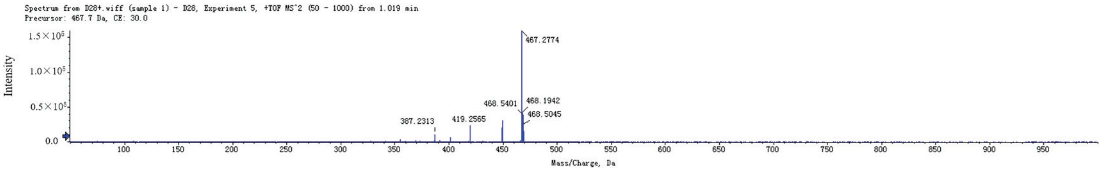

Figure S59. HR-ESI-MS spectrum of uraphine (**10**).

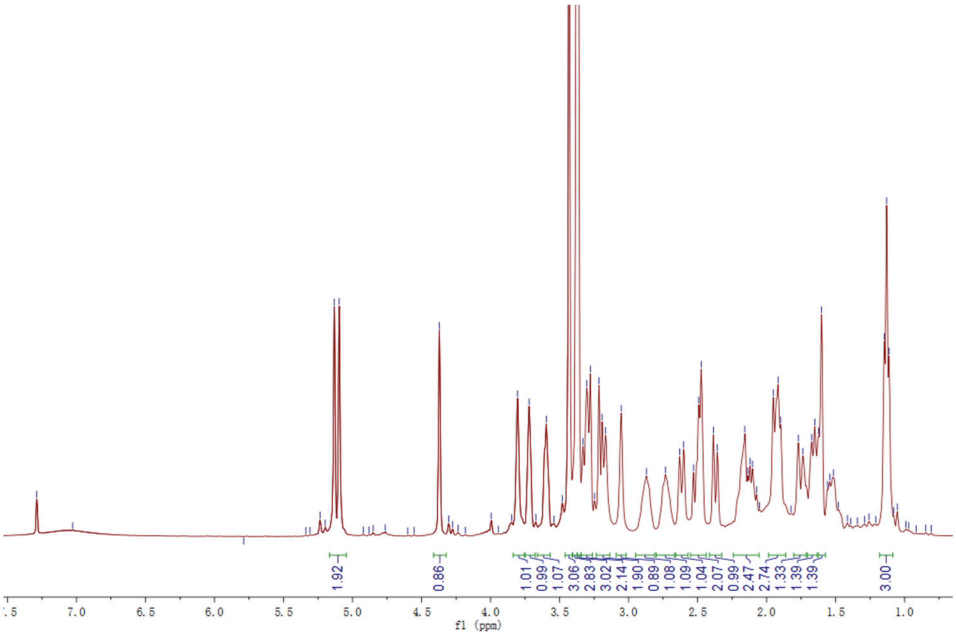

Figure S60. ^{1}H NMR spectrum of uraphine (**10**).

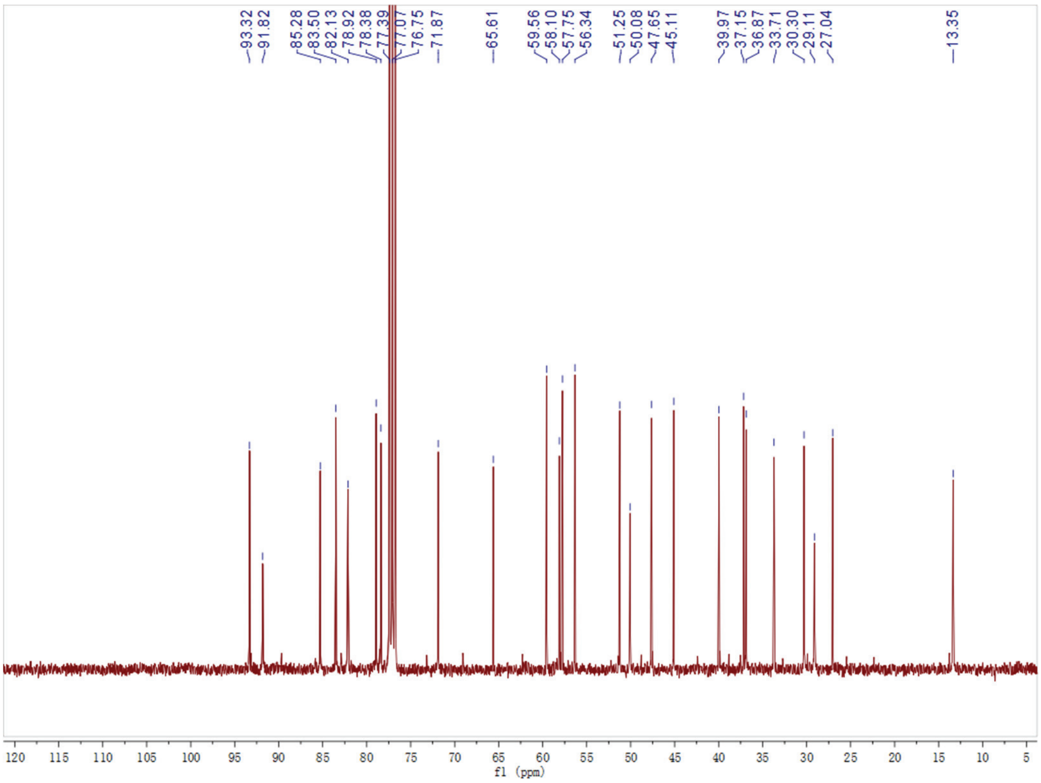

Figure S61. ^{1}C NMR spectrum of uraphine (**10**).

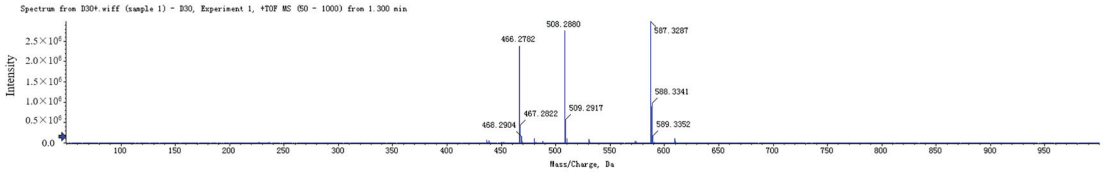

Figure S62. HR-ESI-MS spectrum of anthranoyllycoctonine (**11**).

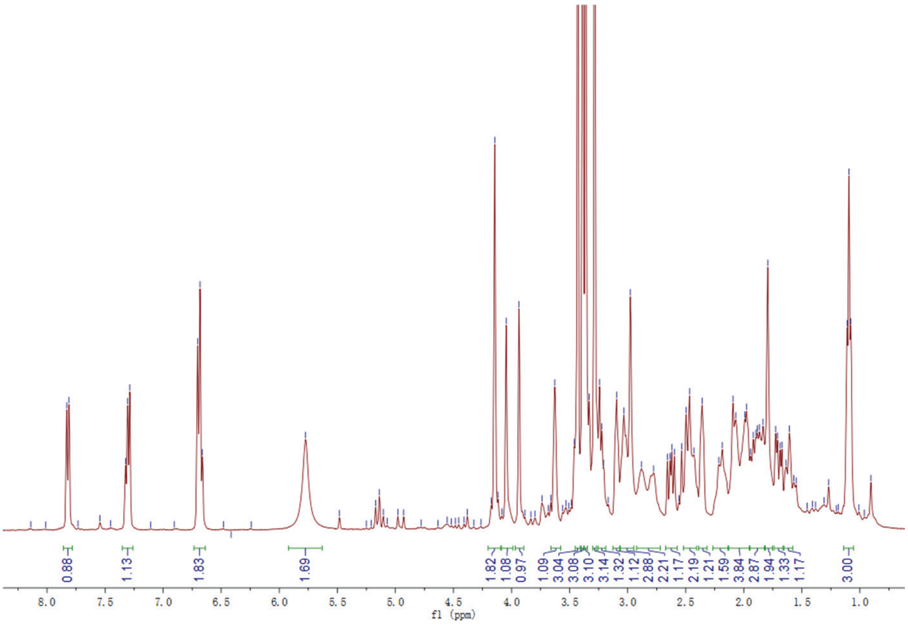

Figure S63. ^{1}H NMR spectrum of anthranoyllycoctonine (**11**).

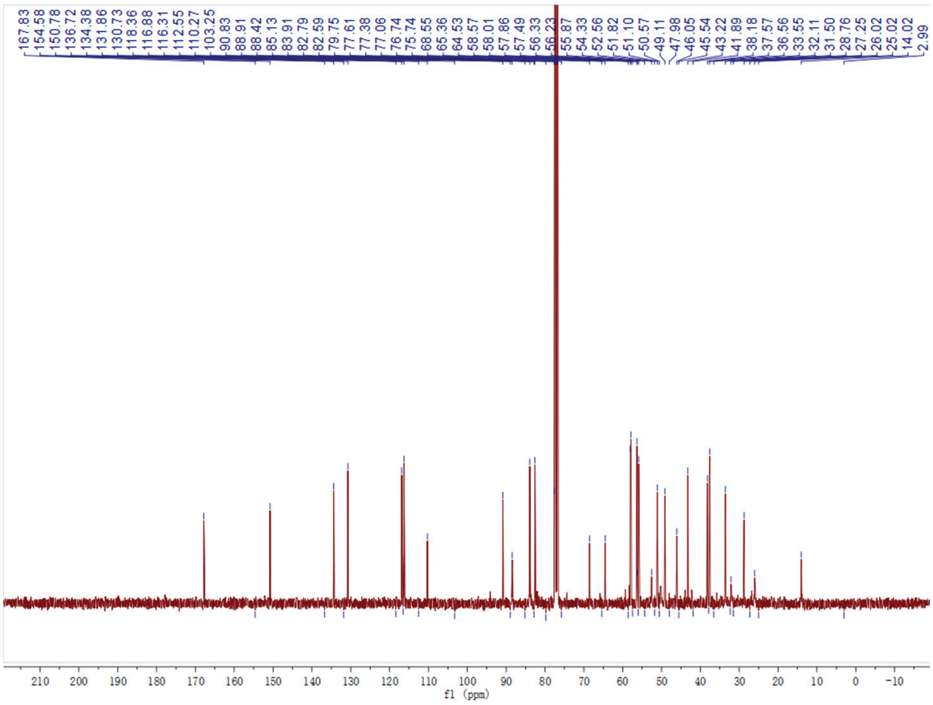

Figure S64. ^{1}C NMR spectrum of anthranoyllycoctonine (**11**).

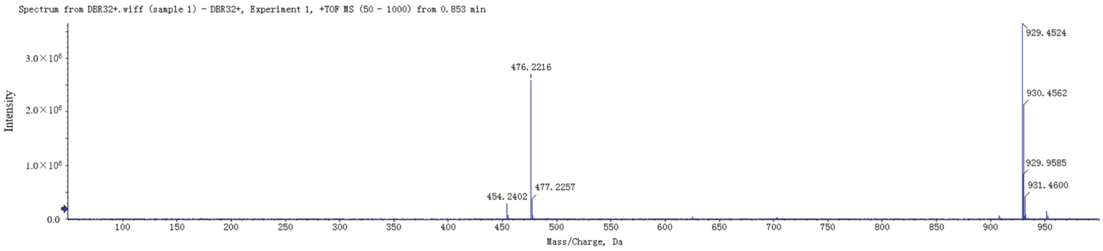

Figure S65. HR-ESI-MS spectrum of sharwuphinine A (**12**).

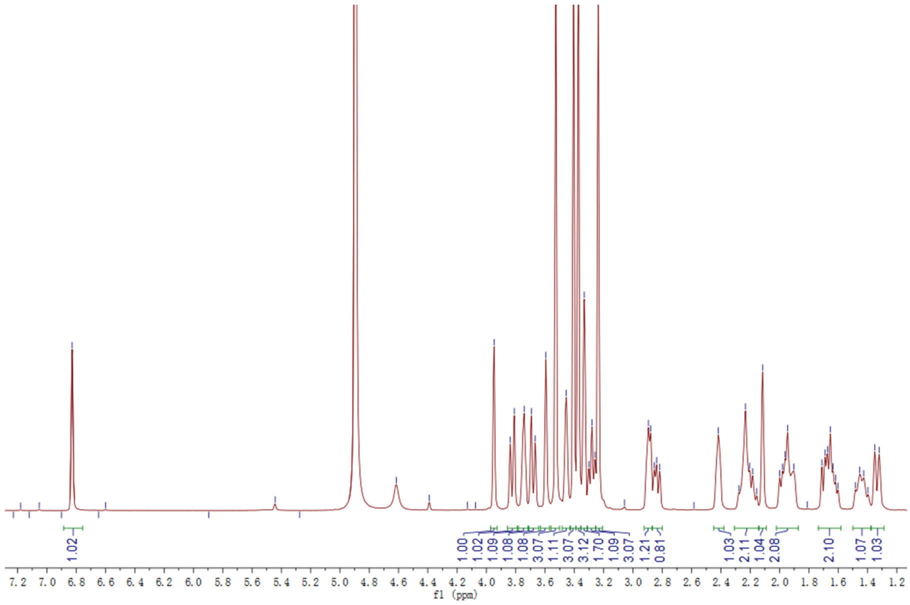

Figure S66. ^{1}H NMR spectrum of sharwuphinine A (**12**).

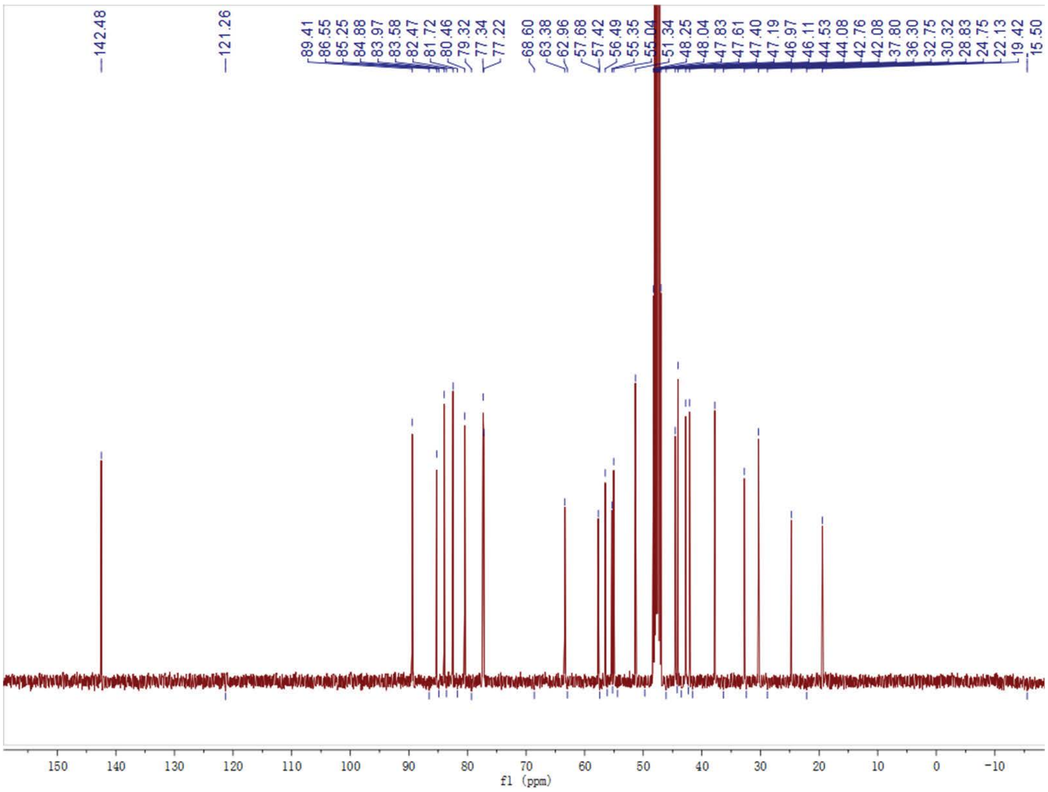

Figure S67. ^{1}C NMR spectrum of sharwuphinine A (**12**).

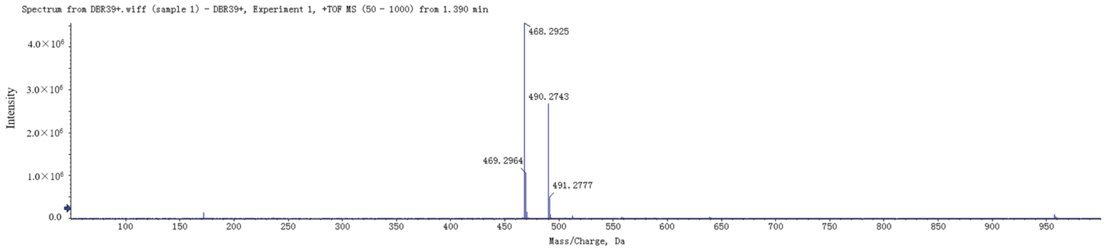

Figure S68. HR-ESI-MS spectrum of browniine (**13**).

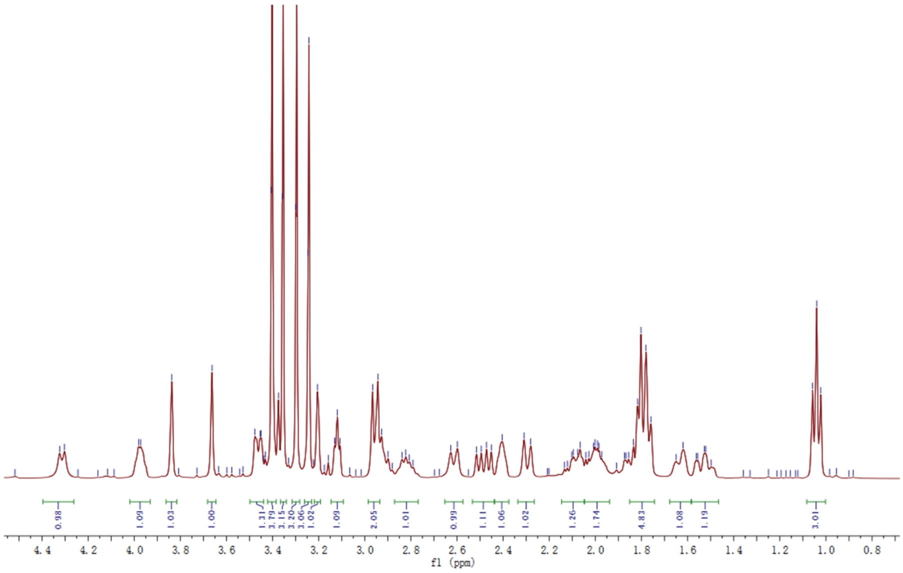

Figure S69. ^{1}H NMR spectrum of browniine (**13**).

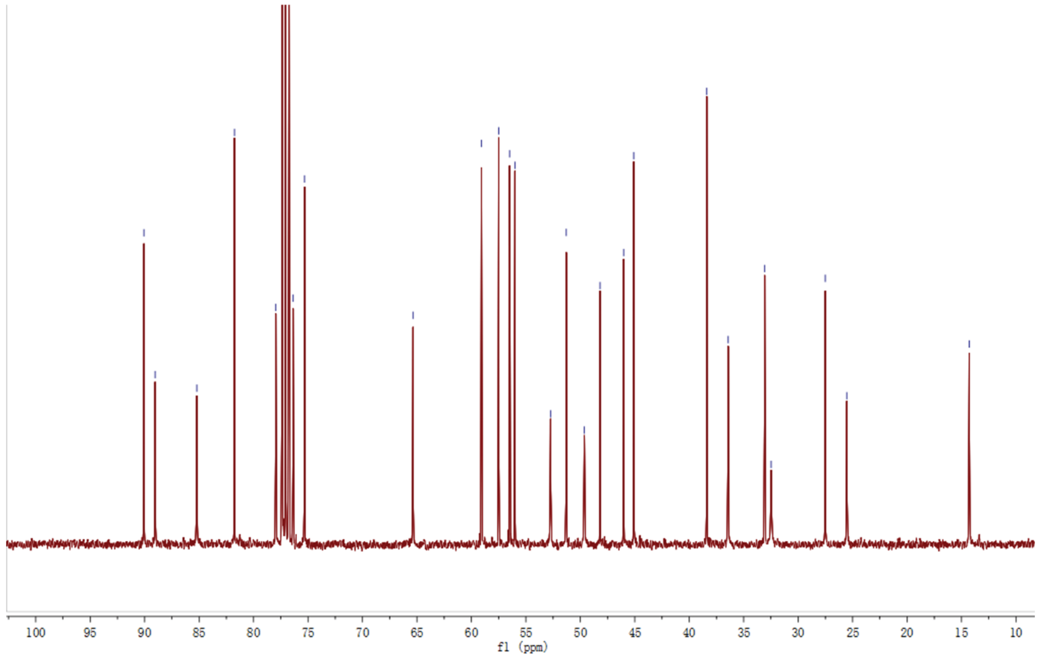

Figure S70. ^{1}C NMR spectrum of browniine (**13**).

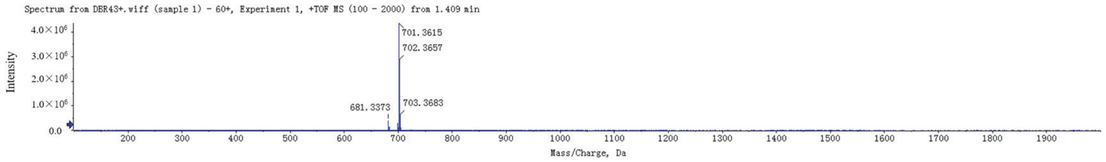

Figure S71. HR-ESI-MS spectrum of shawurensine (**14**).

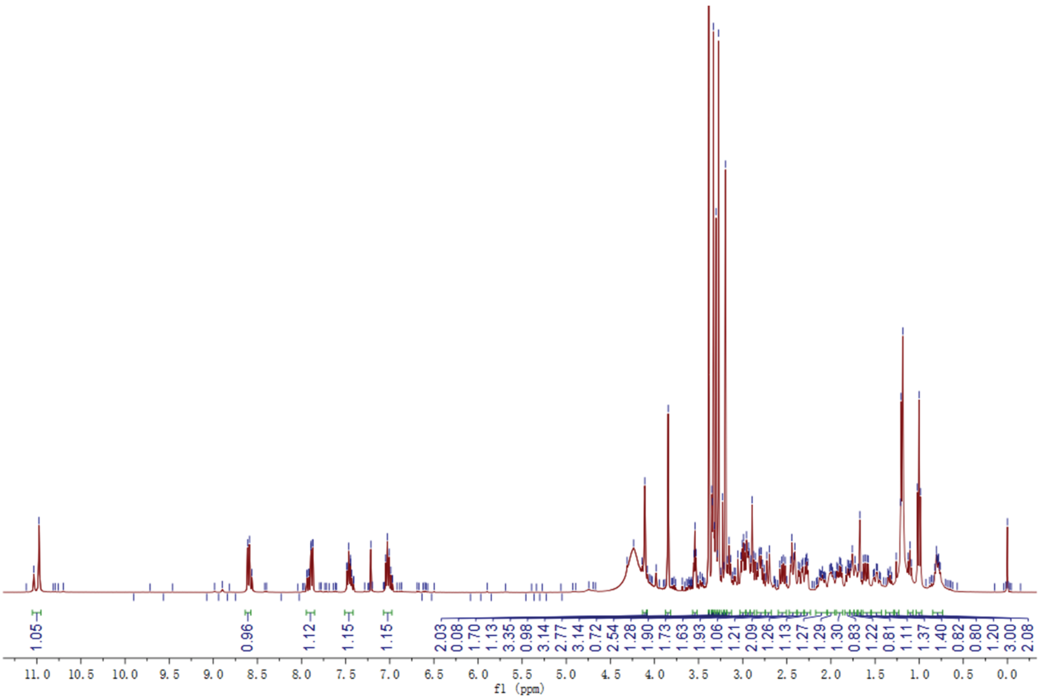

Figure S72. ^{1}H NMR spectrum of shawurensine (**14**).

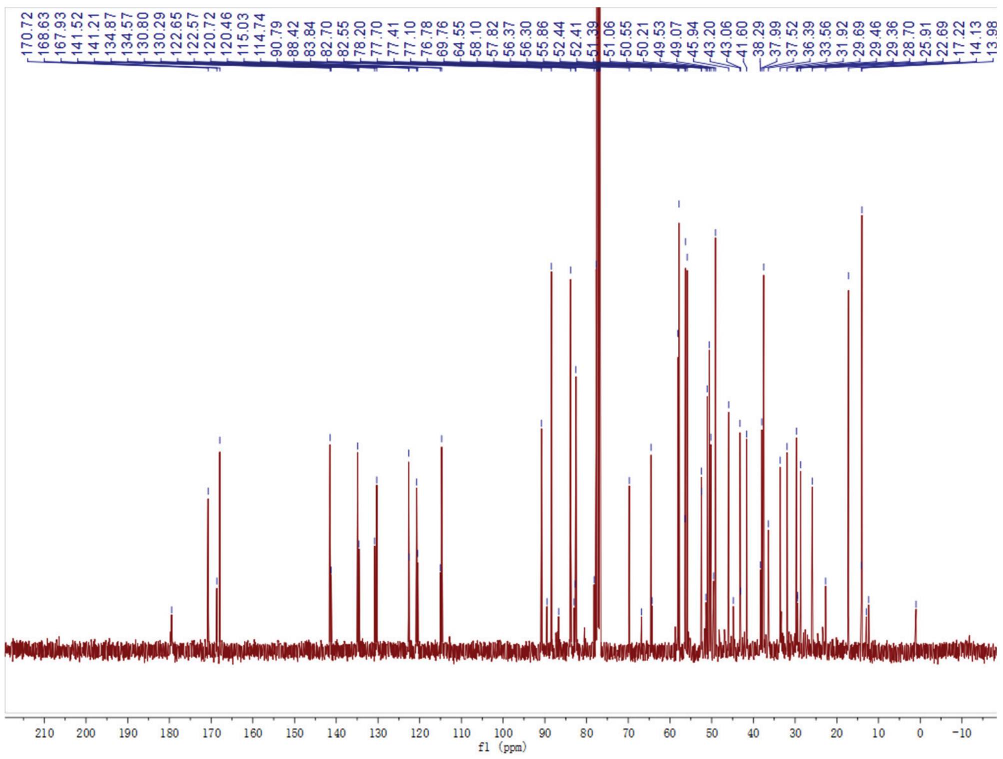

Figure S73. ^{1}C NMR spectrum of shawurensine (14).

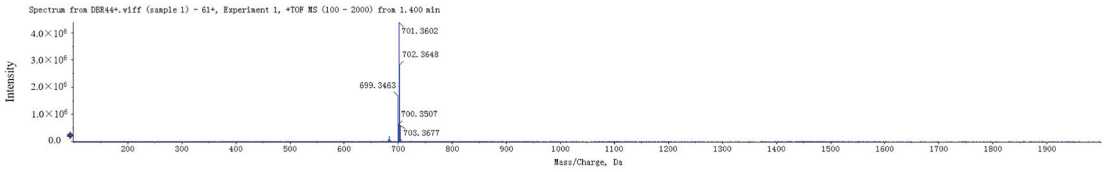

Figure S74. HR-ESI-MS spectrum of delavaine B (**15**).

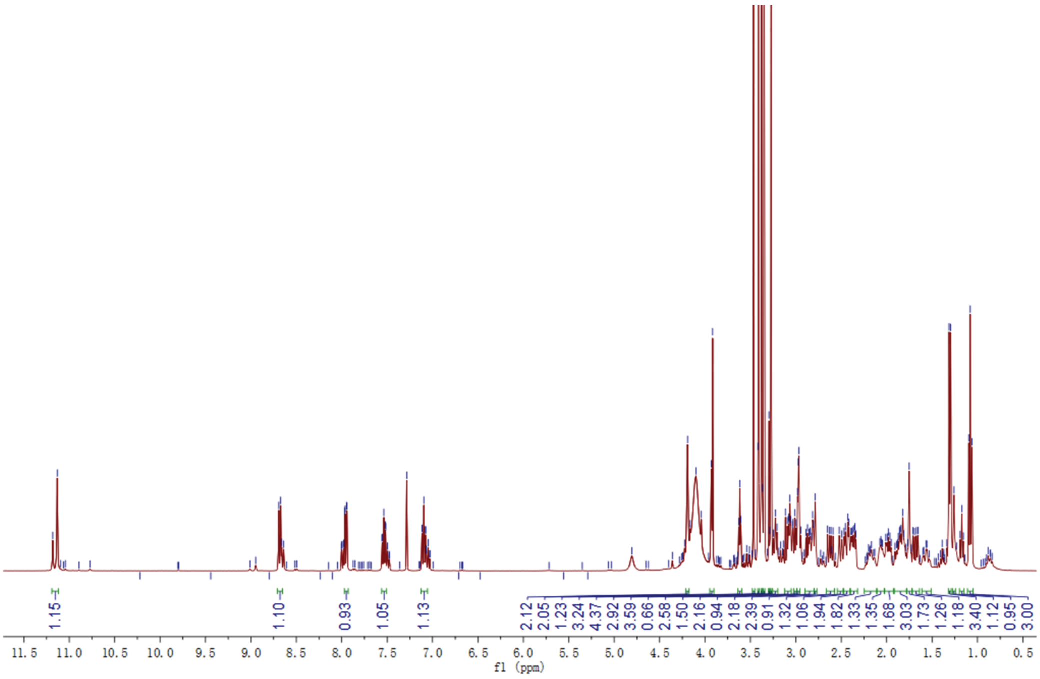

Figure S75. ^{1}H NMR spectrum of delavaine B (**15**).

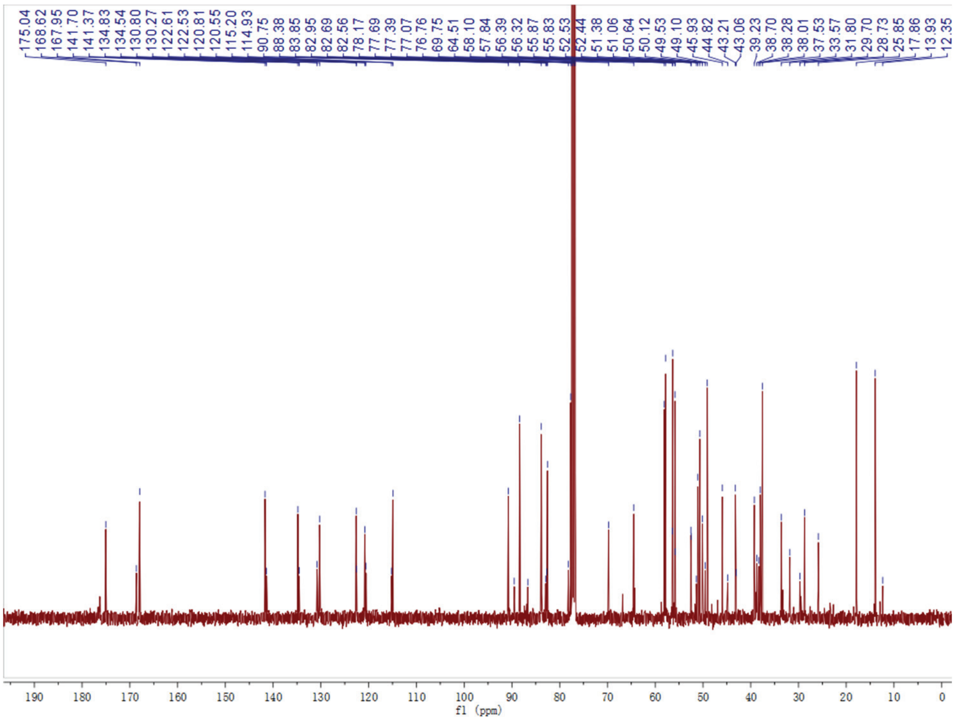

Figure S76. ^{1}C NMR spectrum of delavaine B (**15**).

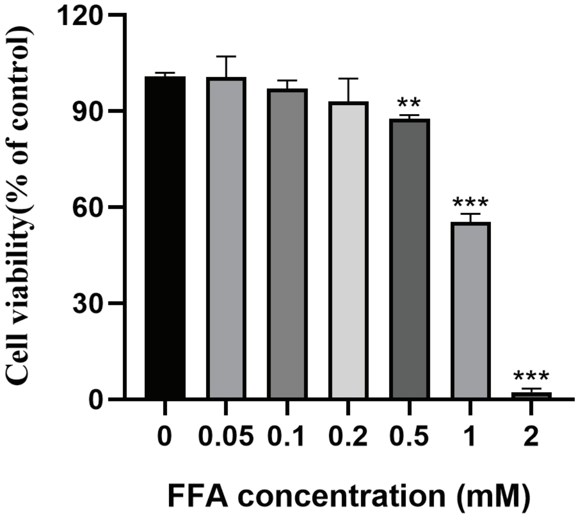

Figure S77. Cell viability at different FFA concentrations in BRL cells

The values are presented as mean ± SEM of six independent experiments. $^{**}p < 0.01$, $^{***}p < 0.001$, vs. the control group.

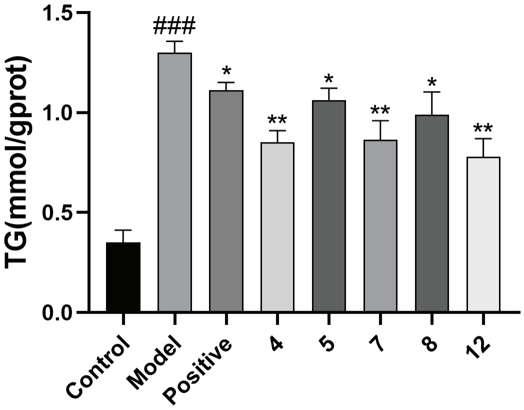

Figure S78. Effects of compounds (**4**, **5**, **7**, **8**, and **12**) on TG level in FFA-induced HepG2 cells. HepG2 cells were treated with a mixture of 1 mM FFA in the absence or presence of compounds (**4**, **5**, **7**, **8**, and **12**) at the concentration of 10 μM. The values are presented as mean ± SEM of six independent experiments. $^{###}p < 0.001$, vs. the control group; $^{*}p < 0.05$, $^{**}p < 0.01$, vs. the model group.

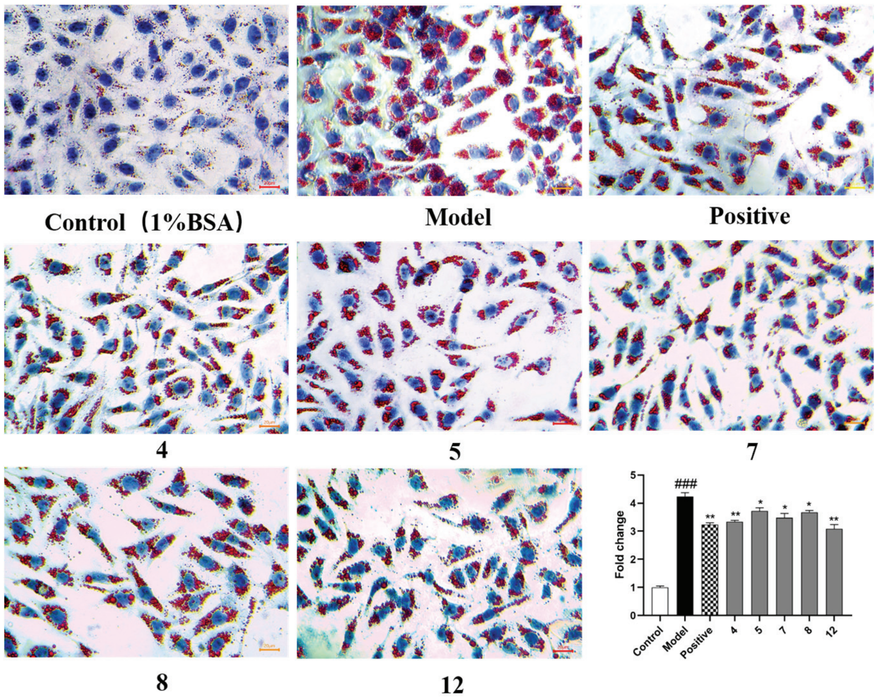

Figure S79. Oil red O staining of lipid droplets and quantitative analysis in HepG2 cells treated with or without 1 mM FFA mixed compounds **4**, **5**, **7**, **8**, and **12** (10 μM), 400 ×; The images shown about quantitative analysis s of red stained cells are representatives of 3 replicates of each. The values are presented as mean ± SEM. $^{###}p < 0.001$, vs. the control group; $^{*}p < 0.05$, $^{**}p < 0.01$, vs. the model group.
